# Quantifying the energetic costs and growth benefits of phagocytosis

**DOI:** 10.1016/j.celrep.2026.118105

**Published:** 2026-10-07

**Authors:** Paul E. Schavemaker, Kaitlyn S. Tam, Michael Lynch

**Affiliations:** 1 Biodesign Center for Mechanisms of Evolution, Arizona State University, Tempe, AZ 85287, USA; 2 School of Life Sciences, Arizona State University, Tempe, AZ 85287, USA; 3 Lead contact

## Abstract

Phagocytosis, the ingestion of cells by other cells, is ubiquitous among eukaryotic life. It is utilized for food uptake and in the immune response in many species. The origin of phagocytosis and its role in the evolution of endomembranes and the eukaryotic cell remains obscure. Drawing on a wealth of empirical data, we integrate prey capture, engulfment, and internal and external digestion into a mathematical model that quantifies the cellular performance of a primitive phagocytoser relative to a non-phagocytosing ancestor. The primitive phagocytoser can outperform the ancestor in a range of situations. The rate of engulfment delineates when phagocytosis is advantageous, providing clear benchmarks for interpreting the importance of results in genetic knockout studies and mechanical models. The phagocytoser even outperforms the ancestor when food vacuoles cannot be recycled. The model suggests plausible waypoints for the evolution of phagocytosis and the eukaryotic endomembrane system.

## In brief

Schavemaker et al. present a mathematical framework for calculating the contribution of phagocytosis, cells eating other cells, to cellular performance as a function of its molecular and cellular makeup. This provides insights into the macroevolutionary origin of phagocytosis and endomembranes, complex internal membrane structures, of eukaryotes.

## Graphical Abstract

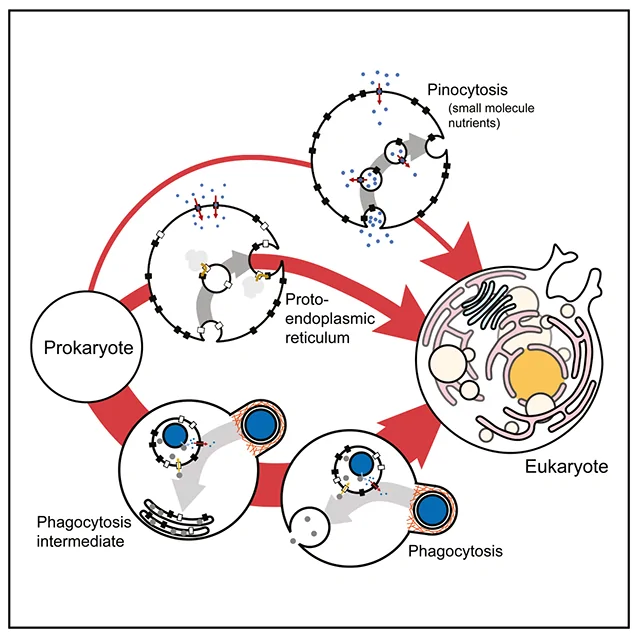

## INTRODUCTION

Cells ingesting other cells, or phagocytosis, is a horrifying spectacle on the microscale in which the life of an organism is snuffed out by being completely engulfed, bathed in acid and hydrolytic enzymes, and finally assimilated into the predator cell. It helps dispose of pathogens in the mammalian immune system^1,2^ and to obtain sustenance in many single-celled species. Phagocytic immune cells may have evolved from nutritionally important enteric phagocytes.^3^ Observed^4^ as far back as the year 1777, the deeper evolutionary roots of phagocytosis lie in the engulfment of prey cells by microbial predators, with representatives of this mode of sustenance extant across present-day unicellular eukaryotes.^5,6^ Phagocytosis occurs in close relatives of the animals, the unicellular choanoflagellates,^7^ as well as the more distantly related amoebae.^8–11^ Phagocytic representatives from other parts of the eukaryotic tree are present in the heliozoa,^12^ dinoflagellates,^13,14^ green algae,^15,16^ haptophytes,^17^ euglenids,^18^ and metamonads.^19,20^ The most spectacular forms of phagocytosis are found in the ciliates.^21–23^

Phagocytosis is ancient, probably having evolved on the order of a billion years ago,^24^ and may be the driving force behind eukaryogenesis.^25–29^ This view is contested, and some evidence suggests that phagocytosis only fully evolved after the last eukaryotic common ancestor (LECA) was already established^24,30,31^ and depended on mitochondria.^32^ The potential for phagocytosis may exist in Asgard archaea,^33,34^ but so far it has not been observed.^35^ The only known case of phagocytosis outside of the eukaryotes is in the planctomycete bacterium Candidatus *Uabimicrobium amorphum*.^36^

Despite great progress in understanding phagocytosis,^1,2,22,37^ the benefit that phagocytosis bestows upon its host has not been quantified, and in scenarios of the evolution of phagocytosis, its benefit is simply assumed.^25–29^ That phagocytosis provides cells with an advantage seems indisputable given its wide adoption among eukaryotes. How large this advantage is, and whether less effective intermediate forms of phagocytosis are still advantageous, is an open question. Equally unclear is the degree to which the advantage depends on the nature of the ancestor, the environment, and presence of traits such as endomembranes.

Quantifying the *net* gain in cellular performance associated with a cellular trait requires the examination of both the gross benefit and the cost of introducing this new trait. A convenient way of accounting for (some of) the negative effects on performance is the relative energetic cost of the newly introduced trait, focusing on opportunity costs.^38–40^ Such cost-benefit analyses have been carried out for flagella,^41,42^ vacuoles,^43^ mitochondria,^44^ and buoyancy.^45^ Here, we present a model for quantitating the gain in cellular performance of phagocytosis, built upon earlier work on pinocytosis and proto-endoplasmic reticulum,^46^ that encompasses prey capture, engulfment, and internal and external digestion. The model compares a derived, phagocytosing state to an ancestral state in which prey is also captured but digested externally.

## RESULTS

### Outline of the phagocytosis model

We consider a predator cell—the phagocytoser—that captures prey cells, engulfs them, and digests them internally. The cellular performance of this phagocytoser is compared, under the assumption of a stable niche, to an evolutionary precursor cell—the ancestor—that also captures prey but digests these externally.

The ancestor and phagocytoser are spherical cells of 1,000 μm^3^ in volume (typical for eukaryotes^44^) and are limited in their growth rate by nutrient flux (smaller cells are also considered). In the ancestor (Figure 1A), prey cells (1 μm^3^ in volume, typical for bacteria^44^) are captured (Figures 1C and 1D) and positioned on the cell surface. Enzymes are excreted by Sec translocases^47^ to convert prey biomass into nutrient molecules that are internalized by nutrient transporters. Both enzymes and nutrients can be lost to the environment. The prey capture mechanism used is that of the heliozoan, which offers some advantages for the modeling that other mechanisms lack (see STAR Methods).

The phagocytoser (Figure 1B) invests in engulfing the prey and digesting it internally to combat the loss of enzymes and nutrients. Prey are captured and subsequently engulfed (Figures 1E and 1F) by a phagocytic cup and internalized in a food vacuole. Sec translocases on the food vacuole import enzymes into the food vacuole lumen, and the nutrients produced by the enzymes are exported into the cytoplasm by nutrient transporters. Finally, the food vacuole fuses with the predator plasma membrane.

### The limited utility of external digestion

There may be environments, e.g., with fluid flow, in which the phagocytoser can exist, but the ancestor cannot because enzymes are lost (similar considerations hold for swimming lifestyles). To examine the feasibility of the ancestor, i.e., its ability to grow at all, we use (derived from Equation 6, see Supplemental Information, Equations S45, S46, and S47):

(Equation 1) $$t_{d,anc}=\frac{\delta_{1}+\delta_{2}+\varepsilon_{2}}{\delta_{3}-\varepsilon_{1}}$$

With quantities representing the base nutrient requirement (*δ*_1_), the prey capture cost (*δ*_2_), nutrient value discounting (ε_2_), the rate of nutrient uptake (*δ*_3_), and the enzyme cost (ε_1_). For *δ*_1_, *δ*_2_, and ε_2_, the units are nutrients (amino acids), and for *δ*_3_ and ε_1_, the units are nutrients per second.

From Equation 1, an immediate insight can be obtained into the feasibility of the ancestor. Enzymes need to be excreted to obtain nutrients (*δ*_3_), but each enzyme also costs nutrients to produce (ε_1_). The difference *δ*_3_–ε_1_ expresses the net nutrient uptake rate, with a negative value signifying lack of growth. In Figure 2A, *δ*_3_–ε_1_ is plotted as a function of *t_dig_*, the prey digestion time, and *f_mod_*, a multiplier that adjusts the base probabilities (*P_enz_* and *P_nut_*) that an enzyme reaches the prey and a nutrient reaches the predator (Equation S46). The base probabilities of enzyme reaching prey, *P_enz_*, and nutrient reaching predator, *P_nut_*, are dependent on the geometry of the predator-prey complex, are different in value, and, for *P_enz_*, depend on the number of prey on the predator surface (the values are obtained from simulations; Figure S1). A lower *f_mod_* means that the ancestor loses more enzymes and nutrients to the environment. Figure 2A reveals that losses of nutrients and enzymes of approximately 75%–99% can be tolerated depending on the digestion time. Confined spaces or proximate kin (to share resources) increase *f_mod_* and could allow the ancestor to persist. A longer digestion time, *t_dig_*, improves the prospects of the ancestor because each individual enzyme has more time to release amino acids from the prey, reducing enzyme requirement (and loss). Figure 2A suggests that certain niches and lifestyles are inaccessible to the ancestor but are accessible to the phagocytoser (because it does not squander as many enzymes).

### Cellular performance of the phagocytoser

The cellular performance of the phagocytoser (Equations 3 and 4) can be compared to that of the ancestor in the same niche. Cellular performance must be calculated in two steps because of the separation of the evolutionary timescale over which the parameter values are optimized by natural selection (first step, Figures 2B and 2C; second step, Figures 2F and 2G). Changing the prey digestion time (*t_dig_*) and the prey capture rate (*v_cap_*) involves simple, and therefore rapid, regulatory changes in the number of Sec translocases and axopods (e.g., caused by base substitutions that adjust promotor strength). The prey engulfment time (*t_eng_*), on the other hand, is decreased during the long process of phagocytosis evolution, in which many new proteins are recruited and optimized. The result is that the value of *t_dig_* is always close to optimal in each step along the evolution of phagocytosis (in our scenario), even if the ancestor and phagocytoser differ significantly in the optimal value of *t_dig_*. Comparing ancestor and phagocytoser with the same value of *t_dig_* is therefore meaningless. The base nutrient return probability (*P_nut_*) relates to the nature of the ancestor (and the situation in which it lives) and is therefore not an optimizable parameter.

The phagocytoser outperforms the ancestor for shorter engulfment times, *t_eng_*, and lower nutrient return probabilities, *P_nut_* (Figures 2F, 2G, and S2). This is true when the cellular performance is calculated from cell division times alone and even more so when the cellular performance accounts for surface area occupancy of the predator plasma membrane. The effect of surface area occupancy is caused by the low area occupancy of phagocytosis (Figures 2D and 2E). Area occupancy is important to consider for large cells because of small surface-to-volume ratios.

### Cellular performance of intermediate forms of the phagocytoser

For phagocytosis, the production of a food vacuole and its subsequent fusion are both complex processes that appear to be essential. To test this essentiality, the consequences of a lack of food vacuole fusion were examined in a modified variant of our model. In addition, intermediate forms of phagocytosis were examined that have (1) low selectivity for the food vacuole membrane proteins and (2) less effective prey engulfment.

For phagocytosis in which food vacuoles do not fuse with the plasma membrane (Figure 3A), the food vacuole abundance will reach a steady state. The food vacuoles will not accumulate indefinitely because growth dilutes the remnant food vacuoles. In fact, the steady state volume occupancy of food vacuoles in the cytoplasm is less than 8% (if food vacuoles collapse into a flat shape; Figure 3A). The cell division time is calculated from (Supplemental Information):

(Equation 2) $$t_{d,phago}=\frac{\alpha_{1}+\alpha_{2}+\alpha_{3}+\beta_{2}}{\alpha_{5}-\beta_{1}-\beta_{3}}$$

The cell division time depends on quantities representing the base nutrient requirement (*α*_1_), the engulfment cost (*α*_2_), the prey capture cost (*α*_3_), and nutrient value discounting (*β*_2_), all expressed in number of nutrients (amino acids). The net nutrient uptake rate, given by the denominator (*α*_5_–*β*_1_–*β*_3_), must be positive for the phagocytosing organism to grow. This means that the rate of nutrient uptake, *α*_5_, must be larger than the sum of the rate of enzyme production (enzymes are lost upon food vacuole fusion), *β*_1_, and the rate of food vacuole membrane production, *β*_3_ (with all three expressed in nutrient number per second). Strikingly, a quantitative examination of the denominator reveals that food vacuole fusion is not required for the phagocytoser to grow because the denominator is always positive (Figure 3B). A phagocytoser without food vacuole fusion can also outperform the ancestor if fractional surface occupancy is important for cellular performance (Figures 3C and 3D).

Low selectivity for Sec translocases and nutrient transporters into the food vacuole in a primitive phagocytoser would lead to the food vacuole being occupied largely by other proteins (Figure 3E), increasing the digestion time and lowering cellular performance (Figure 2C). Similar consequences result from a high requirement for receptors to bind the prey cell and recruit the actin cytoskeleton in the formation of the phagocytic cup. Figures 3F and 3G reveal that even with only 10% of the food vacuole membrane available for Sec translocases and nutrient transporters, the phagocytoser can outperform the ancestor.

Prey engulfment will likely be less effective early in the evolution of phagocytosis, which will present itself in longer engulfment time and larger, more expensive, phagocytic cups. Quantification of cellular performance can reveal whether a proposed mechanism of engulfment is sufficient to outperform the ancestor (Figure 2F, black square) or not (Figure 2F, white square), depending on the engulfment time. With subsequent evolution of the engulfment machinery, engulfment time was reduced and cellular performance improved (Figure 2F, arrow and black disc). Modern forms of phagocytosis have engulfment times of minutes,^48,49^ similar to that of small-vesicle endocytosis^50^ and prey engulfment by dinoflagellates.^51^

Coordination between actin filaments, a product of the many regulatory proteins associated with the actin cytoskeleton, will initially be lacking, potentially leading to large and expensive phagocytic cups (Figure 3H). Cellular performance calculations in which the thickness of the phagocytic cup is increased from 0.11 to 1 μm reveal that the phagocytoser can still outperform the ancestor but only in a reduced parameter range (Figures 3I and 3J).

For understanding gradual evolution of phagocytosis, it is also relevant to examine cells smaller than 1,000 μm^3^. This early phagocytoser may not have captured prey by axopods, so we examine a phagocytoser with an undefined prey capture mechanism with a capture rate that we set (Supplemental Information). Predator volumes in the range 10–1,000 μm^3^ show similar effectiveness of phagocytosis over the tested volume range (Figures 4A and 4B, and S3). Consequences of prey escape from the ancestor (due to lack of engulfment) and increased digestion efficiency in the phagocytoser (e.g., due to acidification of the food vacuole) were also examined and found improvements in the phagocytoser in roughly linear proportion to loss of prey and increased efficiency (Supplemental Information; Figure S4).

## DISCUSSION

A model of phagocytosis is established by which cellular performance is quantified relative to a non-phagocytic ancestor. This model integrates prey capture, engulfment, and external and internal digestion into a single framework, providing a method for judging the biological importance of experimental results that touch on key phagocytosis parameters such as prey engulfment time. The models suggest that phagocytosis can improve cellular performance in the absence of an endomembrane system and that it can do so even when food vacuoles do not fuse with the plasma membrane. Combined with increases in our knowledge of the phagocytosis mechanism, and with the application to additional intermediate states, the model presented here can contribute to our understanding of the macroevolutionary processes that originated phagocytosis and the eukaryotic endomembrane system.

### Gradual evolution of phagocytosis

The sequence of cellular transformations by which phagocytosis evolved is obscured by its considerable molecular complexity.^2,32^ It may have required an intermediate in which prey was partly engulfed.^28,52,53^ A sinking-type phagocytosis as seen in excavates,^54^ and perhaps present in the last eukaryotic common ancestor,^55,56^ may have been an intermediate to the modern diversified forms of phagocytosis across eukaryote diversity.^24,31^ Precursors required for phagocytosis evolution have been found in the Asgard relatives of eukaryotes, including actin cytoskeleton homologues,^57,58^ endomembrane regulators,^59–62^ and SNAREs,^63^ putting an early evolution of phagocytosis in the realm of possibility.

Various studies have revealed how alterations to the mechanism of phagocytosis can affect the engulfment time, a parameter that our theory reveals to be key to phagocytosis success (*t_eng_*, Figures 2F and 2G). Mechanistic models suggest that receptor-only phagocytosis, without actin, is possible.^37,64^ Knockout of the actin-capping protein CapG reduces the uptake rate of zymosan particles by phagocytosis by roughly 2-fold.^65^ Dynamin appears to affect phagocytic cup formation and closure.^66,67^ Integrating these results with our phagocytosis model will deepen our understanding of the contribution of the various molecular factors to phagocytosis.

Progress in understanding the evolution of complex traits faces the challenge of the whole trait seemingly having to come into existence all at once with all its components in place. Examples include eyes and wings on the macroscale^68^ and ATP synthase^69^ and flagella^70^ on the molecular and cellular scale. Nevertheless, convincing explanations for the gradual evolution of such complex traits have been proposed.^68,70^ A similar difficulty arises in trying to explain the evolution of phagocytosis, and it has been claimed that phagocytosis cannot evolve in the absence of the pre-adaptation of vesicle fusion.^25,28^ However, the work presented here suggests that a phagocytoser lacking vacuole fusion can grow, and outperform the ancestor, obviating the need for a vesicle fusion pre-adaptation and rendering a gradual transition more plausible.

### Phagocytosis and the endomembrane system

In modern eukaryotes, phagocytosis and other endomembranes always co-occur. The only prokaryote known to perform phagocytosis is a planctomycete that also contains internal membranes.^36^ This co-occurrence could mean that phagocytosis is not feasible in the absence of an endomembrane system. The phagocytosis model presented here does not explicitly include other components of the endomembrane system and suggests that phagocytosis could evolve in the absence of such components (e.g., enzyme transport to food vacuoles by vesicle fusion).

It is conceivable that some of the parameter values used in the phagocytosis model depend on the existence of the endomembrane system. Internal digestion in modern phagocytosing species depends on the acidification of the food vacuole lumen by the fusion of endosomes and lysosomes,^2^ and it is possible that the rates of prey protein digestion used in the model could only be accomplished in the presence of such acidification, although presumably a food vacuole could also be acidified by V-type ATPases directly.^71^ The production (or delivery) of membrane for phagocytosis may also require the endomembrane system.^2,72^

Phagocytosis could have been the starting point of the endomembrane system.^25–29^ The model presented here suggests that phagocytosis can exist in niches in which the ancestor cannot and suggests a considerable advantage in cellular performance where they can both survive. This is true for a range of parameter values and intermediate states, including an intermediate in which food vacuoles do not recycle. This suggests that phagocytosis is a more likely candidate for the initiation of endomembrane evolution than pinocytosis (of small nutrient molecules) and the proto-endoplasmic reticulum (Figure 4C).^46^ Comparison with other alternative hypotheses of endomembrane evolution^73–78^ should be carried out in the future and will depend on quantitative reformulation of those hypotheses.

### Limitations of the study

The cellular states examined here, the phagocytoser and its non-phagocytic ancestor, are separated by vast evolutionary time and many genetic alterations. As such, this cannot be considered a complete description of the evolutionary pathway to phagocytosis and endomembranes. Additional intermediates, alternative pathways, and changes in niche will have to be considered for a more complete picture. A further caveat with the present work is the absence of an explicit treatment of survival, for example during periods of starvation, and the lack of a more complete accounting of the ecological situation. The cellular states for which the comparisons in cellular performance were made in this work should be considered as signposts that help us understand the macroevolutionary processes that gave rise to phagocytosis and endomembranes, but do not yet give a satisfactory account of the complete process, lacking a description of how specific mutants can invade a population.

## RESOURCE AVAILABILITY

### Lead contact

Requests for further information and resources should be directed to and will be fulfilled by the lead contact, Paul E Schavemaker (pschavem@asu.edu).

### Materials availability

No materials were generated in this study.

## STAR★METHODS

### METHOD DETAILS

#### Calculation of the cellular performance

Cellular performance relates to the growth and survival of an organism and is here defined in relative terms, comparing two cellular states. The formulation of cellular performance used here mostly reflects cell division time but, in some instances, includes survival implicitly. The calculations also assume exponential growth without a stationary period, ignore the energetic cost of operating the cellular traits (only including construction cost, see below), and assume a relatively stable ecological niche. Invasion of specific mutations into a population is not examined as the timescales, and number of genetic alterations, between the examined cellular states are large. As such, the model doesn’t present evolutionary dynamics but simply examines the relative performance of a sequence of cellular states and determines the conditions under which these cellular states are open or closed to evolution. I.e. cellular states lower in performance than ancestral states are unlikely to be attained by evolution whereas cellular states higher in performance may be attained by evolution but only if the underlying evolutionary dynamics, of mutation and fixation, allows it.

Cellular performance is approximated by two methods (Supplemental Information) that abstract away the details of cellular functioning to serve as a framework for future, more detailed and comprehensive, examinations of the contribution of phagocytosis to cellular performance. For both methods, we examine growth rate, which is only one aspect of the cellular performance. The first method is a simple ratio of cell division times,^46^ in which we assume that other aspects of cellular performance, i.e., survival, are equal between phagocytoser and ancestor (Supplemental Information):

(Equation 3) $$Cellular\;performance=\frac{t_{d,anc}}{t_{d,phago}}$$

Where *t_d,anc_* and *t_d,phago_* are the cell division times of the ancestor and phagocytoser, respectively. Equation 3 can be applied when the cell surface area is not limiting for growth and survival. In large cells, which have small area-to-volume ratios, the surface area of the cell is a scarce resource and occupying it will come with a steep cellular performance penalty. To account for this effect (previously included in the cell division time^46^), a second method to calculate cellular performance is used:

(Equation 4) $$Cellular\;performance=\frac{t_{d,anc}}{t_{d,phago}}\frac{\left(1-f_{occ,phago}\right)}{\left(1-f_{occ,anc}\right)}$$

Here, *f_occ, phago_* is the fractional occupancy of the plasma membrane by the phagocytosis and prey capture machinery in the phagocytoser and *f_occ,anc_* is the equivalent for the ancestor. The surface of the cell is essential for its growth and survival, so a fractional decrease of available surface area is likely to have a proportional fractional effect on the cellular performance. Other relationships between surface area occupancy and the impact on cellular performance are conceivable and will have to be examined in the future. Unlike the first method, including surface area occupancy can include the effects of survival, for example if proteins important for survival (e.g., for osmoprotection or toxin expulsion) are displaced from the surface area. Note that the outputs of Equations 3 and 4 resemble fitness but that the invasion and fixation of individual mutants in a population is not examined, so the term cellular performance is used instead.

The cellular states that are examined here would never encounter one another in the same environment, because they are likely to exist millions of years apart. The cellular performance calculations compare how well each cellular state would do in a specific set of environments. This is relevant to an evolutionary scenario for the large-scale change of cellular features. If we were to find that there would be no environments in which phagocytosis could function better than the ancestor, this would constitute evidence against phagocytosis as an intermediate toward the endomembrane system. Finding that phagocytosis does improve the prospects of a cell in a specific set of environments also suggests that intermediates between the phagocytoser and ancestor have a cellular performance somewhere in between that of these states and would improve upon the ancestor as well, with the caveat that there may be bottlenecks along the way that need to be examined in the future and that the process of mutations invading a population is not examined here.

Cell division time is a key contributor to cellular performance, even in species that grow relatively slowly (like phagotrophic eukaryotes compared to some bacterial species like *Escherichia coli*). This is because growth rate is a consequence of a specific niche and, even when slow, could be a main contributor simply because it is difficult to grow fast in this environment and with this lifestyle. This may be true for phagocytosis, where the fact that nutrients first need to be processed, rather than used directly as soon as they are imported (as in some bacteria), and the requirement to be large, impose fundamental limitations on growth rate.

Under the assumption of nutrient limited growth, the cell division time is the ratio of the nutrient requirement for producing a new cell and the nutrient acquisition rate^46^:

(Equation 5) $$t_{d}=\frac{Nutrient\;requirement}{\;Nutrient\;acquisition\;rate}$$

For the ancestor, this becomes (see Supplemental Information):

(Equation 6) $$t_{d,anc}=\frac{N_{nutV}V_{pred}\left(1+C_{trait,anc}\right)}{N_{AA,prey}P_{nut}v_{capadj}N_{value,anc}}$$

Here, *N_nutV_* is the nutrient requirement per unit of predator volume, *V_pred_* is the predator volume, *C_trait,anc_* is the relative energetic cost of prey capture and external digestion in the ancestor, *N_AA,prey_* is the number of nutrients (amino acids) present in a prey cell, *P_nut_* is the base probability that a nutrient produced by external digestion reaches the predator, *v_capadj_* is the prey capture rate adjusted for cell growth (see Supplemental Information, calculated from the prey capture rate *v_cap_*), and *N_value,anc_* is the average nutrient value in the ancestor that accounts for a delay in nutrient return to the predator (see Supplemental Information). As an example of loss of nutrient value, consider a batch of nutrient molecules that takes a whole cell division period to return to the predator. It will lose half its value as it will need to supply two cells instead of one.

For the phagocytoser the cell division time becomes:

(Equation 7) $$t_{d,phago}=\frac{N_{nutV}V_{pred}\left(1+C_{trait,phago}\right)}{N_{AA,prey}v_{capadj}N_{value,phago}}$$

This does not include *P_nut_* because the prey is digested internally and no nutrients are lost. *C_trait, phago_* includes prey capture, engulfment, and internal digestion costs.

The cellular performance calculation involves two steps and overall, four parameters are varied: the prey digestion time (*t_dig_*), the prey capture rate (*v_cap_*), the prey engulfment time in the phagocytoser (*t_eng_*), and the nutrient return probability (*P_nut_*). The first step in the cellular performance calculation is a comparison of ancestor-to-ancestor, and phagocytoser-to-phagocytoser, in which the parameter values of *t_dig_* and *v_cap_* are varied (Figures 2B and 2C), and *t_eng_* and *P_nut_* are kept constant. One arbitrary combination of *t_dig_* and *v_cap_* is chosen as a reference point to which all others are compared. The digestion time optimum found for the phagocytoser, 35 min, is consistent with values reported for ciliates (20 min to 1 h).^22^ In the second step, the parameter values associated with the optima from the first step (Figures 2B and 2C, black squares) are used to calculate the cellular performance of the phagocytoser relative to the ancestor. This two-step cellular performance calculation is repeated for a range of engulfment times, *t_eng_*, and nutrient-return probabilities, *P_nut_* (Figures 2F and 2G).

#### Prey capture

For prey capture, the heliozoan mechanism was mainly used because it allows for the calculation of the relation between energetic cost, cell surface occupancy, and prey capture rate, which are all important for the cellular performance calculation. For other prey capture mechanisms, such as in ciliates and amoebae, these relationships require more sophisticated models not available at present. The heliozoan prey capture mechanism is mechanistically relatively simple and has evolved multiple times,^81^ suggesting that it is easy to evolve. Combined with the fact that the Asgard archaeal relatives of eukaryotes have membranous protrusion supported by cytoskeletal filaments,^58^ a feature also present in heliozoa, a heliozoan-like prey capture mechanism can’t be ruled out as the primitive prey capture mechanism, even though every presently known case has evolved after the diversification of eukaryotes.

Heliozoan prey capture and subsequent phagocystosis had been observed for *Actinophrys sol* by Albert von Koelliker^4^ in 1848. It involves long cellular protrusions called axopods that capture prey passively and transport it to the predator cell surface to be phagocytosed. Axopods consist of a lipid membrane that is extended out from the plasma membrane by a bundle of microtubules. Axopods can capture a variety of prey of different sizes, including bacteria.^82^ Axopod properties, used to calculate energy cost and area occupancy, were extracted from the literature (Figures S1A and S1B; Tables S1 and S2).

Prey capture in the model is based on prey cell diffusion and was quantitatively examined by simulations in the software Smoldyn^80^ (see Supplemental Information, Figure 1C), yielding the relation between the number of axopods, *N_axo_*, and the prey capture rate, *v_cap_* (Figure 1D):

(Equation 8) $$v_{cap}=\frac{Ac_{prey}N_{axo}}{B+N_{axo}}$$

This is an empirical fit to the data and has no theoretical justification, but it is similar to a Michaelis-Menten equation for enzyme kinetics. Here, *A* and *B* are fit parameters and *c_prey_* is the prey concentration. The number of axopods can be related to the axopod energy cost and axopod area occupancy (see Supplemental Information). As the prey concentration is not varied in this study, we haven’t examined saturation of prey uptake rate with prey concentration,^83^ and it is assumed that prey concentration is non-saturating.

#### Prey external digestion

In the ancestor, prey is digested externally by enzymes secreted, at a known rate,^84^ from plasma membrane-localized protein exporters, called Sec translocases,^47^ all over the predator cell surface. The base probability with which these enzymes find the prey by random diffusion, *P_enz_*, is determined by Smoldyn simulations^80^ as a function of the prey number that is present on the predator surface simultaneously (Supplemental Information). When only one prey cell is present *P_enz_* = 0.067 (Figure S1C), but this can go up to 1 for larger prey numbers (Figure S1E). Enzymes are assumed to have anchors keeping them attached to the prey, similar to anchoring of enzymes to the predator cell surface.^28^ At the prey, the enzymes produce nutrients (amino acids) that reach the predator with a base probability, *P_nut_*, of 0.88 (determined by Smoldyn simulation, Figure S1D, Supplemental Information). For simplicity, the prey is assumed to consist entirely of protein, with the rate of amino acid production estimated from digestion rates found in the BRENDA database^85^ (Figure S1F; Table S3). The energy cost of external digestion depends on the number of excreted enzymes, and the area occupancy depends on the number of Sec translocases and nutrient transporters (Supplemental Information). To investigate the limits under which the ancestor can survive, the base numbers for the enzyme to prey probability (*P_enz_*) and the nutrient return probability (*P_nut_*), as calculated above, are multiplied by a factor, *f_mod_* (Equation S46). The factor *f_mod_* is introduced to adjust *P_enz_* and *P_nut_* simultaneously, as both may depend similarly on fluid flow, confinement, and prey-predator distance. This can’t be accomplished by putting *P_enz_* equal to *P_nut_* because they have different base values, and because *P_enz_* depends on the number of prey cells being digested simultaneously while *P_nut_* doesn’t.

#### Prey engulfment

To facilitate estimating the size of the engulfment machinery, and thereby its energetic cost and area occupancy, only protrusion-based engulfment, in which a phagocytic cup emerges from the surface of the predator cell,^48,86^ is examined here (Supplemental Information). The energetic cost of engulfment is the sum of the membrane cost and the phagocytic cup plasm cost, with the plasm cost residing in actin filaments^87–91^ and actin accessory proteins (myosin, arp2/3, etc.).^50,92,93^ In the absence of a quantitative description of the actin cytoskeleton of the phagocytic cup, data were acquired from other membrane structures shaped by the actin cytoskeleton: filopodia,^87^ lamellipodia,^88^ and endocytic vesicles.^89^ This information is combined with energetic cost of membranes^42,94^ and with the geometry and size of phagocytic cups from mouse macrophage^48^ and the choanoflagellate *Codosiga*.^86^ In addition to phagocytosis, the energy cost of an endocytic vesicle from *Schizosaccharomyces pombe*^46,50^ was included in the analysis. The energetic costs calculated here contain only construction costs, operating costs are not accounted for and are expected to contribute only to a minor degree (but this needs to be examined in the future). The energy cost of engulfment is shown in Figure 1E. The cost per unit of engulfed volume (Figure 1F) reveals that larger cups are cheaper per unit of internalized volume. This is due to a smaller area-to-volume ratio and potentially makes larger cups easier to evolve than smaller cups and explaining why phagocytic cups are largely absent from small cells.

#### Prey internal digestion

The product of engulfment is a food vacuole, consisting of a membrane surrounding a prey cell. The food vacuole membrane houses Sec translocases, to import enzymes into the food vacuole lumen, and nutrient transporters, to transport amino acids from the vacuole lumen into the cytoplasm. After the prey is digested, the food vacuole membrane fuses back to the predator plasma membrane and the enzymes, present in the food vacuole lumen, are lost to the external medium. Internal digestion is treated similarly to external digestion except that all the nutrients are recovered and all enzymes make it to the prey (Supplemental Information).

#### Choice of parameter values

Where possible, the model is supported by parameter values extracted from experimental work, simulations, and theoretical analyses. Parameter values used to obtain the results are listed with their sources in Table S4.

### QUANTIFICATION AND STATISTICAL ANALYSIS

There are no quantification or statistical analyses to include in this study.

## Supplementary Material

1

2

Supplemental information can be found online at https://doi.org/10.1016/j.celrep.2026.118105

## Figures and Tables

**Figure 1.**
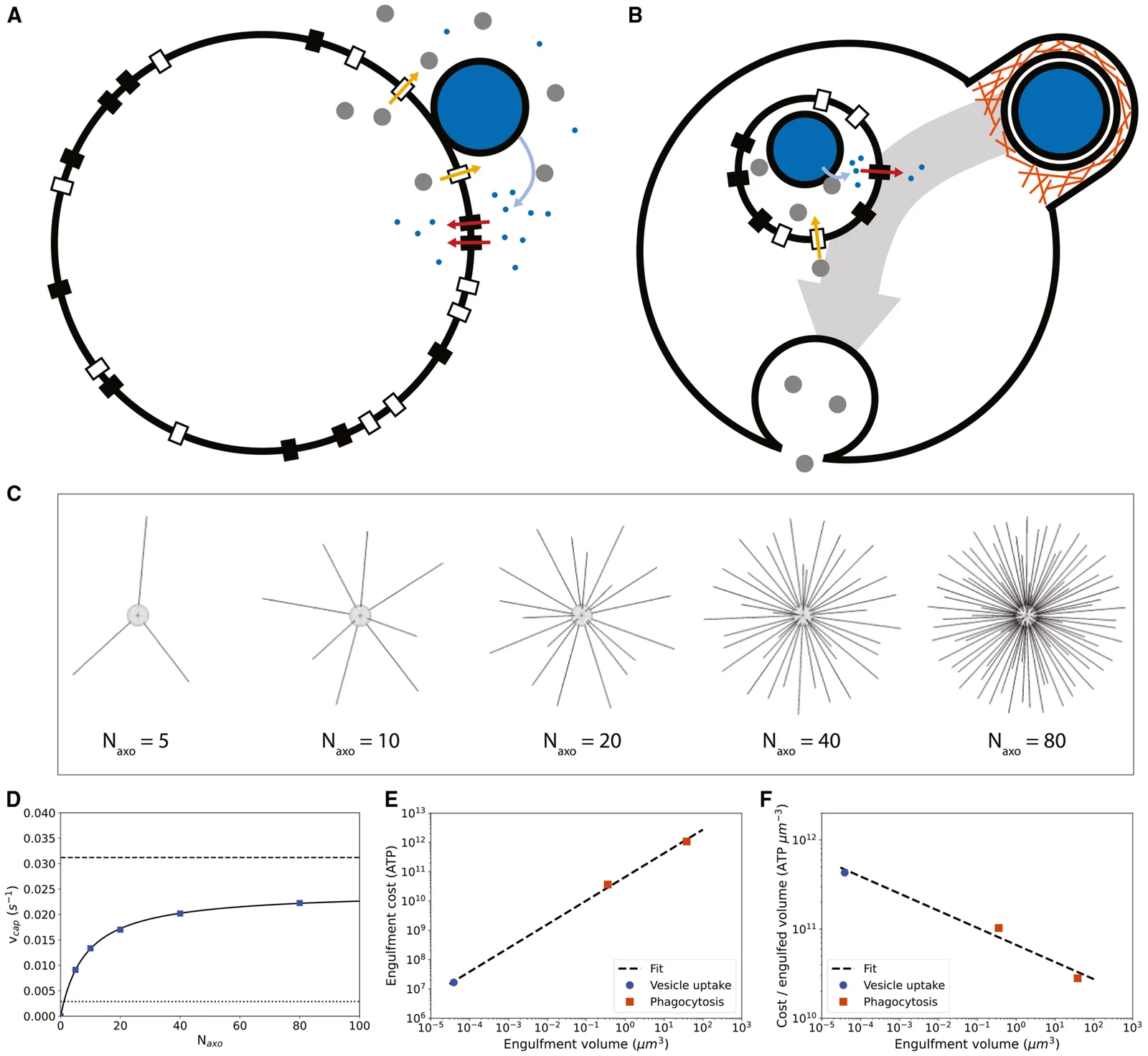
Phagocytosis model, prey capture, and prey engulfment (A) Schematic of the ancestor in the phagocytosis model. Prey capture by axopods is not shown. For visual simplicity, only Sec translocases and nutrient transporters close to the prey are shown as active. Large white circle: predator cell; large blue circle: prey cell; gray circles: enzymes; small blue circles: amino acids; black rectangles: amino acid transporters; white rectangles: Sec translocases. (B) Schematic of the phagocytoser in the phagocytosis model. Orange lines: actin filaments; small (internal) white circle: food vacuole. (C) Smoldyn model of heliozoan prey capture with varying axopod number, *N_axo_*. All axopods have equal lengths. (D) Prey capture rate, *v_cap_*, as a function of axopod number. (E) Energy cost of engulfment for phagocytosis and vesicle uptake. Regression: *y* = 6.65 × 10^10^x^0.81^. (F) Energy cost per engulfed volume for phagocytosis and vesicle uptake. Regression: *y* = 6.65 × 10^10^x^−0.19^.

**Figure 2.**
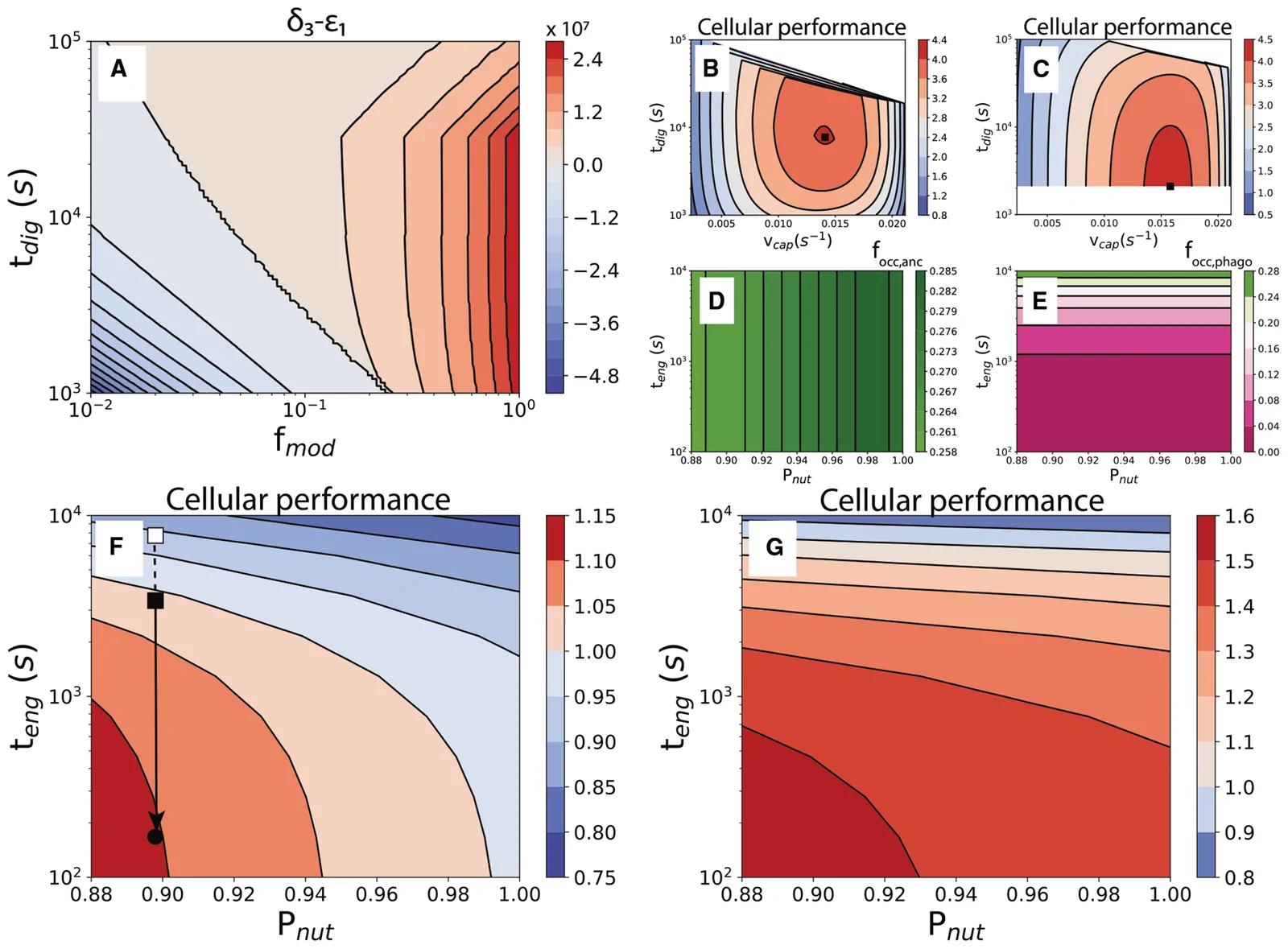
Phagocytosis improves cellular performance (A) Feasibility of the ancestor. Plot of the net nutrient uptake rate, *δ*_3_–*ε*_1_, as a function of the digestion time, *t_dig_*, and the modifier, *f_mod_*. *P_nut_* is 0.88, and *P_enz_* is 0.21–1.00, depending on *t_dig_*. The sudden reduction in *δ*_3_–*ε*_1_ as *t_dig_* increases is caused by the prey occupying the full surface of the predator. (B) Cellular performance of ancestral states with different digestion times, *t_dig_*, and prey capture rates, *v_cap_*, compared to one arbitrary ancestral state from the same set. Result for *t_eng_* = 167 s and *P_nut_* = 0.88. Black square: maximal cellular performance, used for the comparison between the ancestor and the phagocytoser in (G). Cellular performance calculated from Equation 4. White space covers datapoints removed by filters for food vacuole area occupancy (Supplemental Information). (C) Cellular performance of the phagocytosing states as in (B). (D) Fractional occupancy of the cell surface by external digestion and prey capture in the ancestor. (E) Fractional occupancy of the cell surface by engulfment and prey capture in the phagocytoser. (F) Cellular performance of the phagocytoser compared with the ancestor, as a function of the nutrient return probability, *P_nut_*, and the engulfment time, *t_eng_*. Calculated from cell division times only (Equation 3). White square: *t_eng_* insufficient for the evolution of phagocytosis, Black square: *t_eng_* sufficient for the evolution of phagocytosis, Black disc: present day engulfment time. (G) As in (F) but with cellular performance calculated from cell division times and surface area occupancy (Equation 4). Related to Figure S2.

**Figure 3.**
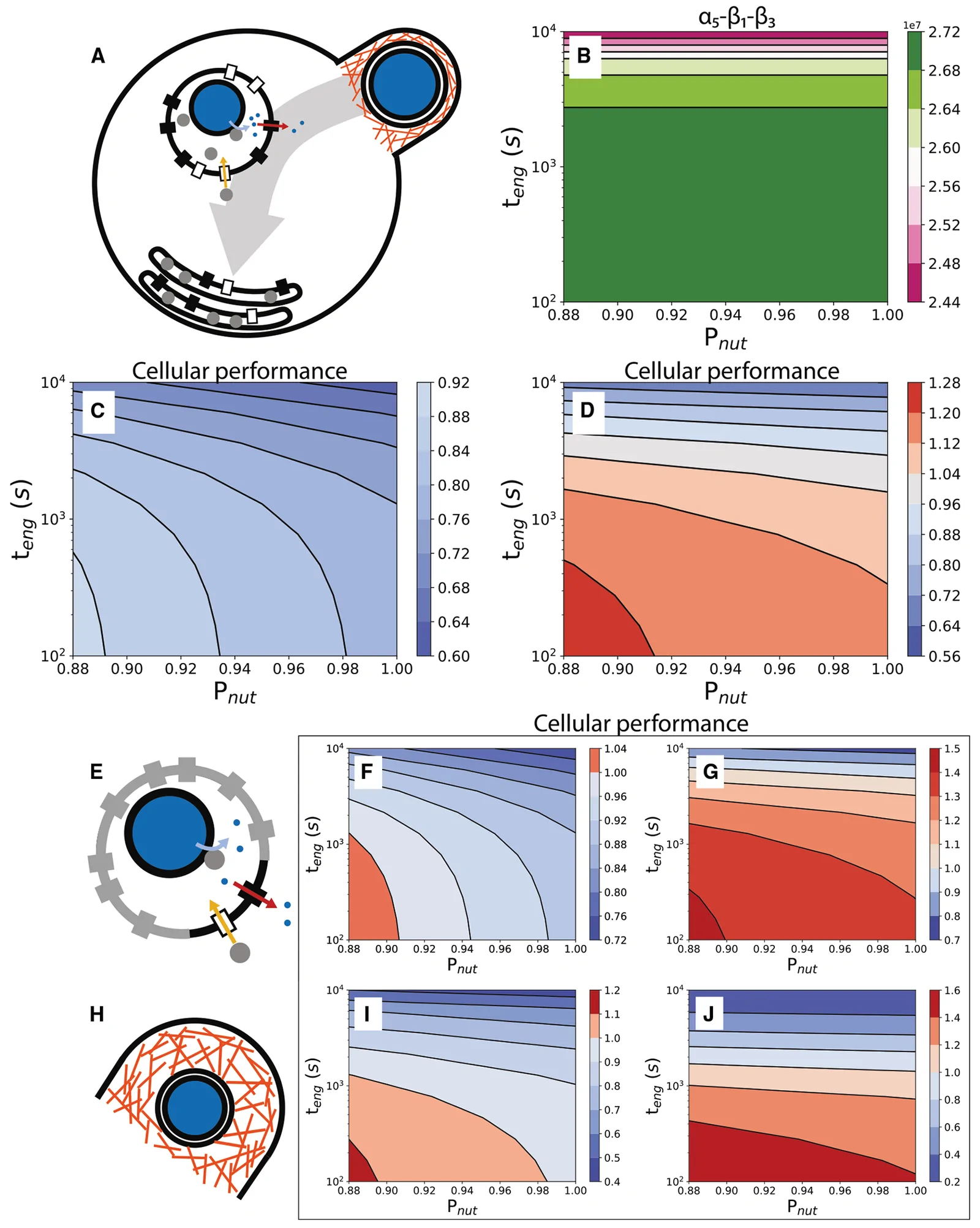
Intermediate forms of phagocytosis improve cellular performance (A) Schematic of a phagocytoser without food vacuole fusion. Food vacuoles accumulate to a steady state number as flattened membrane compartments. (B) Feasibility of phagocytosis without food vacuole fusion. Plot of the net nutrient uptake rate, *α*_5_–*β*_1_–*β*_3_, as a function of the engulfment time, *t_eng_*, and nutrient return probability, *P_nut_*. (C) Cellular performance of the phagocytoser compared with the ancestor in the absence of food vacuole fusion, calculated from cell division times only (Equation 3). (D) As in (C) but with cellular performance calculated from cell division times and surface area occupancy (Equation 4). (E–G) Cellular performance calculation in which 90% of the food vacuole surface is occupied by other membrane proteins. (E) Schematic of a food vacuole, with membrane proteins other than Sec translocases and nutrient transporters in gray. (F) Cellular performance calculated using cell division times only (Equation 3). (G) Cellular performance calculated using both cell division time and surface area occupation (Equation 4). (H–J) Cellular performance calculation for a phagocytoser with larger phagocytic cups. (H) Schematic of a larger phagocytic cup. (I and J) As in (F) and (G).

**Figure 4.**
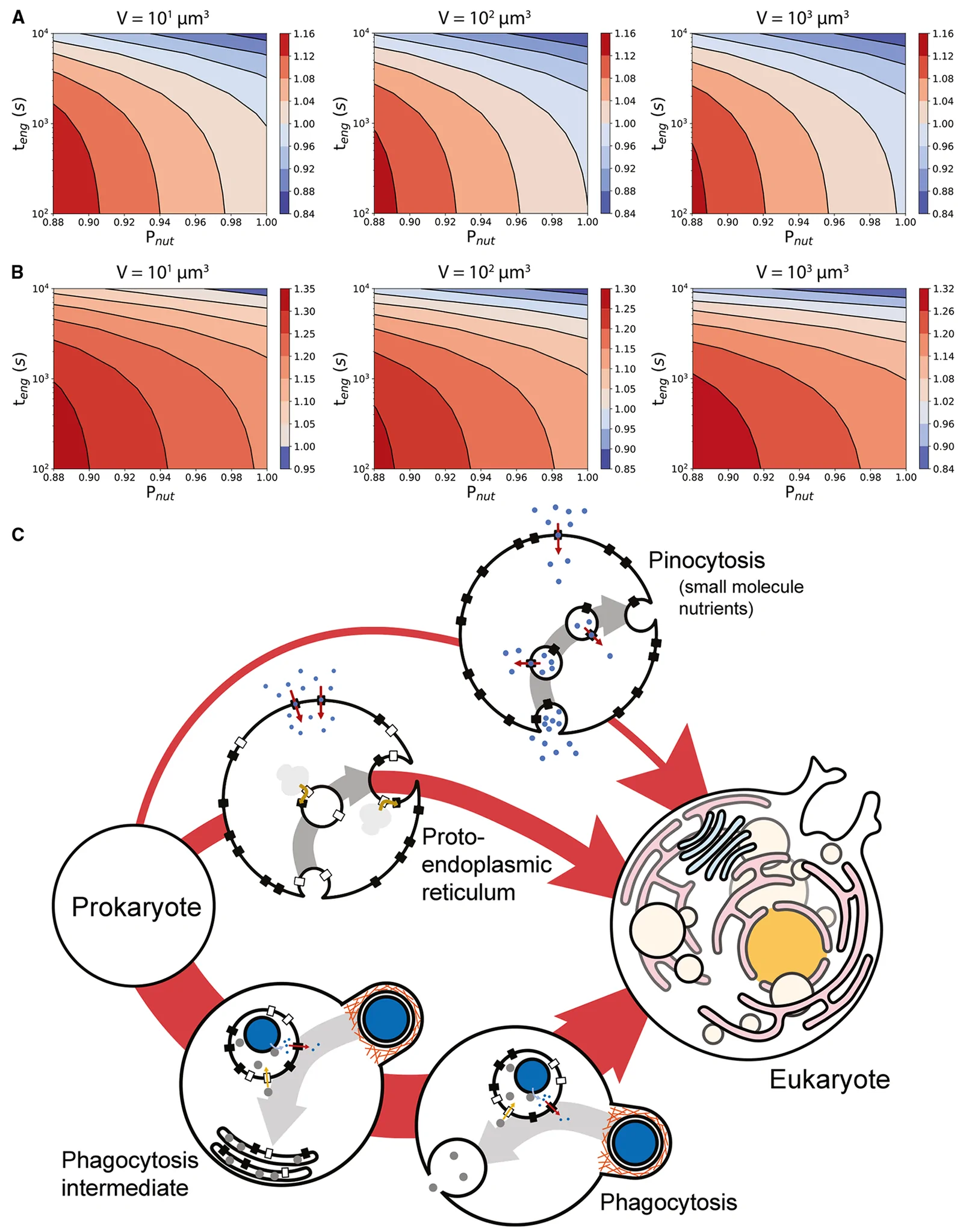
Phagocytosis and the evolution of the endomembrane system (A) The cellular performance contribution of phagocytosis, accounting only for cell division time (Equations 3, S67, and S68), as a function of predator volume. Prey capture uses a base rate of 0.007 prey per second but is lowered for the smaller predators (Supplemental Information). Related to Figure S3. (B) The contribution to cellular performance of phagocytosis, accounting for both cell division time and area occupancy (Equations 4 and S67–S70), as a function of predator volume. The prey capture rates are the same as in (A). Related to Figure S3. (C) Pathways from cells without complex endomembranes to cells with complex endomembranes for which cellular performance calculations have been performed.^46^ The likelihood of the evolutionary pathways is proportional to the thickness of the red arrows.

**Table:** KEY RESOURCES TABLE

REAGENT or RESOURCE	SOURCE	IDENTIFIER
Software and algorithms		
Wolfram Mathematica	Wolfram Research	https://www.wolfram.com/mathematica/
Python 3.8.2	Python Software Foundation	https://www.python.org/
Matplotlib	Hunter et al.^79^	https://matplotlib.org/
Smoldyn	Andrews et al.^80^	https://www.smoldyn.org/

## Data Availability

This paper analyzes existing, publicly available data, cited in the main text. This paper does not report original code. Any additional information required to reanalyze the results presented in this paper is available from the lead contact upon request.

## Highlights

- Phagocytosis costs and benefits can be quantified by a mathematical model
- Phagocytosis improves cellular performance even when food vacuoles are not recycled
- Phagocytosis appears to be effective even for smaller predator cell volumes
- Primitive phagocytosis is a potential pathway to complex eukaryotic endomembranes
