# Supplementary material for: Quantifying the energetic costs and growth benefits of phagocytosis
Source: Cell Rep. Author manuscript; Available in PMC 2026 Nov 16. (PMC13771945; doi:10.1016/j.celrep.2026.118105)
Supplement: Supplementary file 2 [file NIHMS2219875-supplement-2.pdf]

Cell Reports

Report

Quantifying the energetic costs and growth benefits of phagocytosis

Graphical abstract

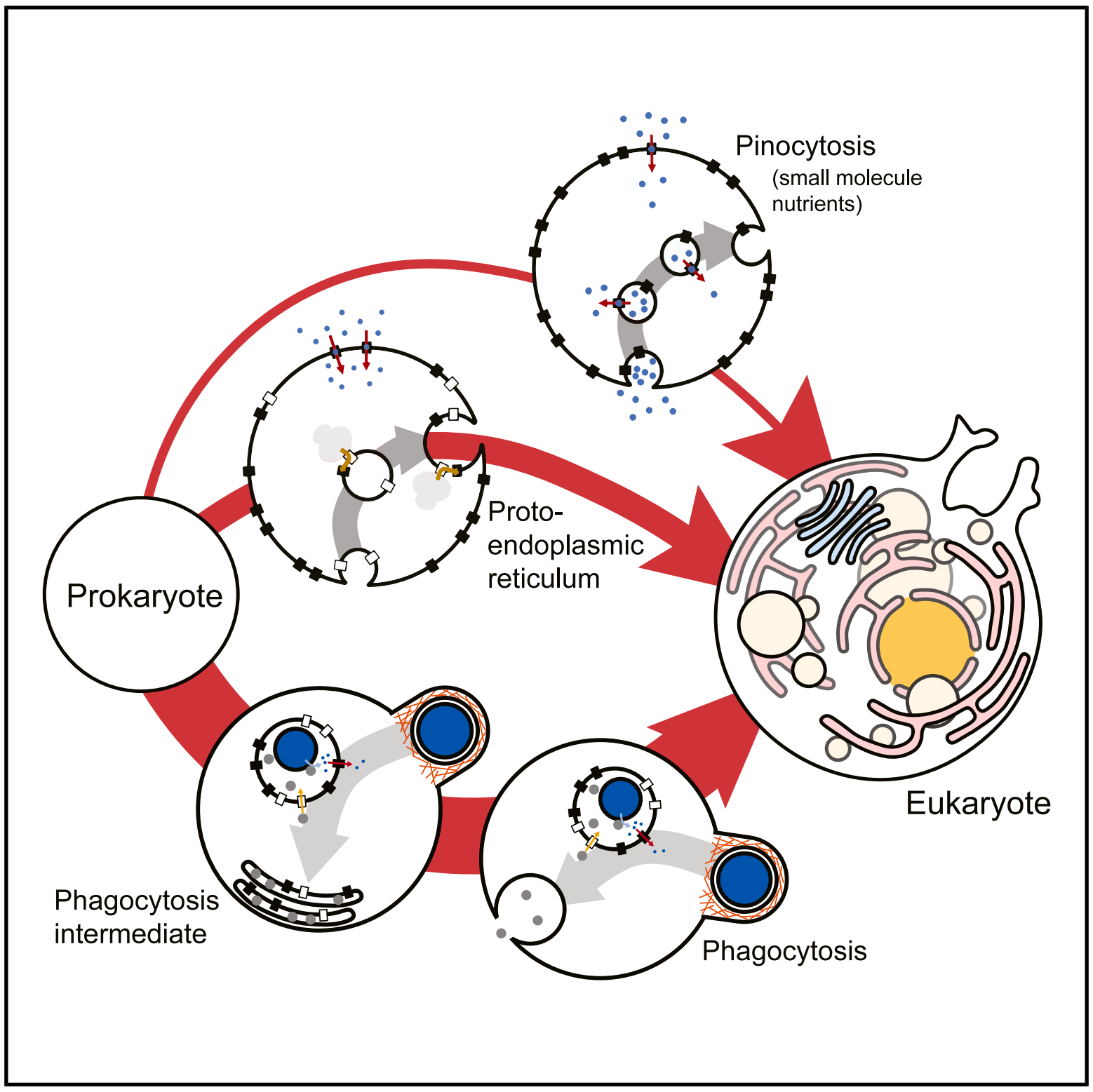

Authors

Paul E. Schavemaker, Kaitlyn S. Tam, Michael Lynch

Correspondence

pschavem@asu.edu

In brief

Schavemaker et al. present a mathematical framework for calculating the contribution of phagocytosis, cells eating other cells, to cellular performance as a function of its molecular and cellular makeup. This provides insights into the macroevolutionary origin of phagocytosis and endomembranes, complex internal membrane structures, of eukaryotes.

Highlights

- Phagocytosis costs and benefits can be quantified by a mathematical model
- Phagocytosis improves cellular performance even when food vacuoles are not recycled
- Phagocytosis appears to be effective even for smaller predator cell volumes
- Primitive phagocytosis is a potential pathway to complex eukaryotic endomembranes

Schavemaker et al., 2026, Cell Reports *45*, 118105
October 27, 2026 © 2026 The Authors. Published by Elsevier Inc.
https://doi.org/10.1016/j.celrep.2026.118105

Cell Reports

CellPress
OPEN ACCESS

Report

Quantifying the energetic costs and growth benefits of phagocytosis

Paul E. Schavemaker,[1,3,*] Kaitlyn S. Tam,[2] and Michael Lynch[1]
[1]Biodesign Center for Mechanisms of Evolution, Arizona State University, Tempe, AZ 85287, USA
[2]School of Life Sciences, Arizona State University, Tempe, AZ 85287, USA
[3]Lead contact
*Correspondence: pschavem@asu.edu
https://doi.org/10.1016/j.celrep.2026.118105

SUMMARY

Phagocytosis, the ingestion of cells by other cells, is ubiquitous among eukaryotic life. It is utilized for food uptake and in the immune response in many species. The origin of phagocytosis and its role in the evolution of endomembranes and the eukaryotic cell remains obscure. Drawing on a wealth of empirical data, we integrate prey capture, engulfment, and internal and external digestion into a mathematical model that quantifies the cellular performance of a primitive phagocytoser relative to a non-phagocytosing ancestor. The primitive phagocytoser can outperform the ancestor in a range of situations. The rate of engulfment delineates when phagocytosis is advantageous, providing clear benchmarks for interpreting the importance of results in genetic knockout studies and mechanical models. The phagocytoser even outperforms the ancestor when food vacuoles cannot be recycled. The model suggests plausible waypoints for the evolution of phagocytosis and the eukaryotic endomembrane system.

INTRODUCTION

Cells ingesting other cells, or phagocytosis, is a horrifying spectacle on the microscale in which the life of an organism is snuffed out by being completely engulfed, bathed in acid and hydrolytic enzymes, and finally assimilated into the predator cell. It helps dispose of pathogens in the mammalian immune system[1,2] and to obtain sustenance in many single-celled species. Phagocytic immune cells may have evolved from nutritionally important enteric phagocytes.[3] Observed[4] as far back as the year 1777, the deeper evolutionary roots of phagocytosis lie in the engulfment of prey cells by microbial predators, with representatives of this mode of sustenance extant across present-day unicellular eukaryotes.[5,6] Phagocytosis occurs in close relatives of the animals, the unicellular choanoflagellates,[7] as well as the more distantly related amoebae.[8–11] Phagocytic representatives from other parts of the eukaryotic tree are present in the heliozoa,[12] dinoflagellates,[13,14] green algae,[15,16] haptophytes,[17] euglenids,[18] and metamonads.[19,20] The most spectacular forms of phagocytosis are found in the ciliates.[21–23]

Phagocytosis is ancient, probably having evolved on the order of a billion years ago,[24] and may be the driving force behind eukaryogenesis.[25–29] This view is contested, and some evidence suggests that phagocytosis only fully evolved after the last eukaryotic common ancestor (LECA) was already established[24,30,31] and depended on mitochondria.[32] The potential for phagocytosis may exist in Asgard archaea,[33,34] but so far it has not been observed.[35] The only known case of phagocytosis outside of the eukaryotes is in the planctomycete bacterium Candidatus *Uabimicrobium amorphum.*[36]

Despite great progress in understanding phagocytosis,[1,2,22,37] the benefit that phagocytosis bestows upon its host has not been quantified, and in scenarios of the evolution of phagocytosis, its benefit is simply assumed.[25–29] That phagocytosis provides cells with an advantage seems indisputable given its wide adoption among eukaryotes. How large this advantage is, and whether less effective intermediate forms of phagocytosis are still advantageous, is an open question. Equally unclear is the degree to which the advantage depends on the nature of the ancestor, the environment, and presence of traits such as endomembranes.

Quantifying the *net* gain in cellular performance associated with a cellular trait requires the examination of both the gross benefit and the cost of introducing this new trait. A convenient way of accounting for (some of) the negative effects on performance is the relative energetic cost of the newly introduced trait, focusing on opportunity costs.[38–40] Such cost-benefit analyses have been carried out for flagella,[41,42] vacuoles,[43] mitochondria,[44] and buoyancy.[45] Here, we present a model for quantitating the gain in cellular performance of phagocytosis, built upon earlier work on pinocytosis and proto-endoplasmic reticulum,[46] that encompasses prey capture, engulfment, and internal and external digestion. The model compares a derived, phagocytosing state to an ancestral state in which prey is also captured but digested externally.

© 2026 The Authors. Published by Elsevier Inc.
This is an open access article under the CC BY license (http://creativecommons.org/licenses/by/4.0/).

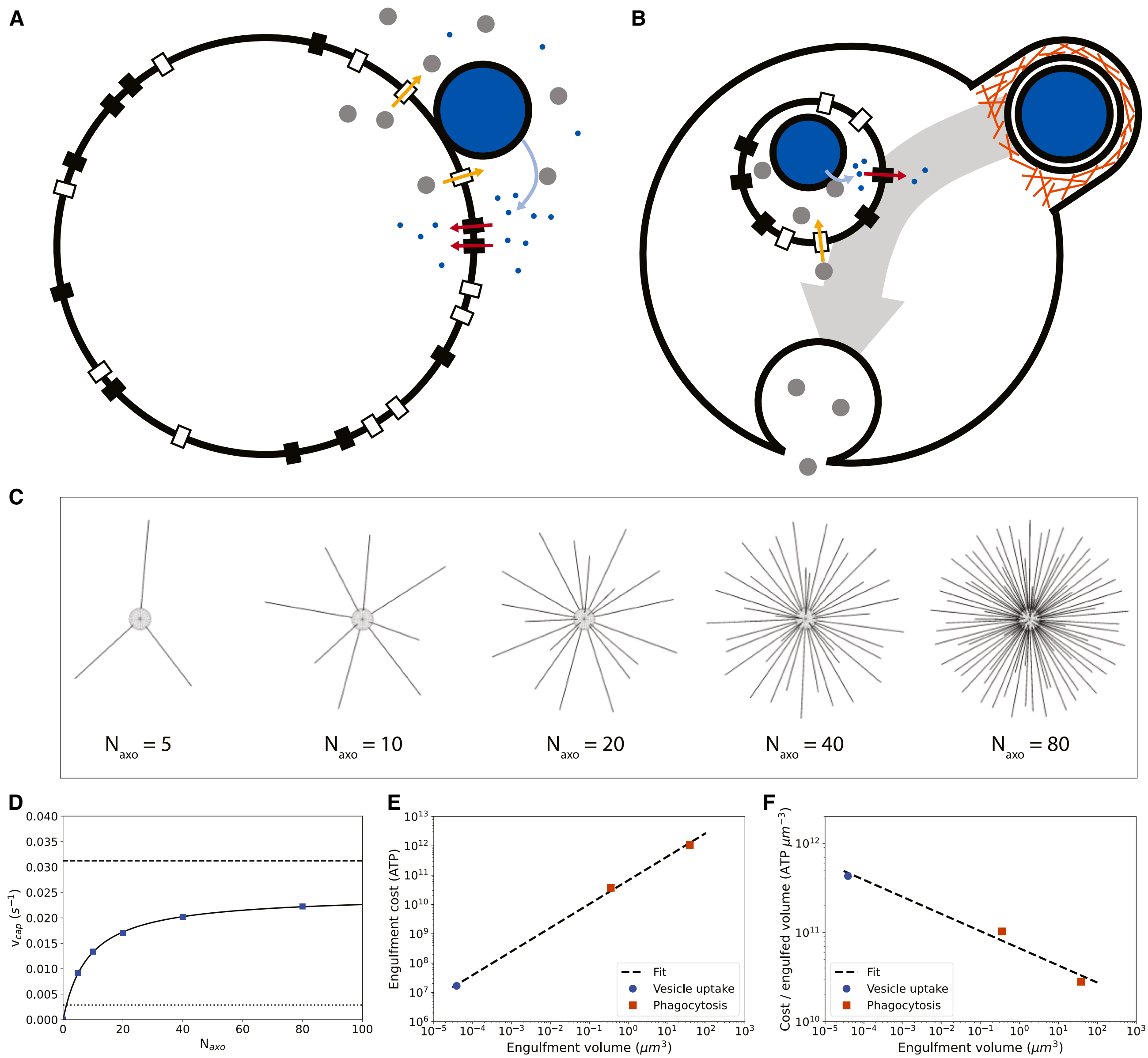

Figure 1. Phagocytosis model, prey capture, and prey engulfment

(A) Schematic of the ancestor in the phagocytosis model. Prey capture by axopods is not shown. For visual simplicity, only Sec translocases and nutrient transporters close to the prey are shown as active. Large white circle: predator cell; large blue circle: prey cell; gray circles: enzymes; small blue circles: amino acids; black rectangles: amino acid transporters; white rectangles: Sec translocases.

(B) Schematic of the phagocytoser in the phagocytosis model. Orange lines: actin filaments; small (internal) white circle: food vacuole.

(C) Smoldyn model of heliozoan prey capture with varying axopod number, N_{axo}. All axopods have equal lengths.

(D) Prey capture rate, v_{cap}, as a function of axopod number.

(E) Energy cost of engulfment for phagocytosis and vesicle uptake. Regression: $y = 6.65 \times 10^{10} x^{0.81}$.

(F) Energy cost per engulfed volume for phagocytosis and vesicle uptake. Regression: $y = 6.65 \times 10^{10} x^{-0.19}$.

RESULTS

Outline of the phagocytosis model

We consider a predator cell—the phagocytoser—that captures prey cells, engulfs them, and digests them internally. The cellular performance of this phagocytoser is compared, under the assumption of a stable niche, to an evolutionary precursor cell—the ancestor—that also captures prey but digests these externally.

The ancestor and phagocytoser are spherical cells of 1,000 μm^3 in volume (typical for eukaryotes[44]) and are limited in their growth rate by nutrient flux (smaller cells are also considered). In the ancestor (Figure 1A), prey cells (1 μm^3 in volume,

typical for bacteria[44]) are captured (Figures 1C and 1D) and positioned on the cell surface. Enzymes are excreted by Sec translocases[47] to convert prey biomass into nutrient molecules that are internalized by nutrient transporters. Both enzymes and nutrients can be lost to the environment. The prey capture mechanism used is that of the heliozoan, which offers some advantages for the modeling that other mechanisms lack (see STAR Methods).

The phagocytoser (Figure 1B) invests in engulfing the prey and digesting it internally to combat the loss of enzymes and nutrients. Prey are captured and subsequently engulfed (Figures 1E and 1F) by a phagocytic cup and internalized in a food vacuole. Sec translocases on the food vacuole import enzymes into the food vacuole lumen, and the nutrients produced by the enzymes are exported into the cytoplasm by nutrient transporters. Finally, the food vacuole fuses with the predator plasma membrane.

The limited utility of external digestion

There may be environments, e.g., with fluid flow, in which the phagocytoser can exist, but the ancestor cannot because enzymes are lost (similar considerations hold for swimming lifestyles). To examine the feasibility of the ancestor, i.e., its ability to grow at all, we use (derived from Equation 6, see Supplemental Information, Equations S45, S46, and S47):

$$t_{d,anc} = \frac{\delta_1 + \delta_2 + \varepsilon_2}{\delta_3 - \varepsilon_1} \qquad \text{(Equation 1)}$$

With quantities representing the base nutrient requirement (δ_1), the prey capture cost (δ_2), nutrient value discounting (ε_2), the rate of nutrient uptake (δ_3), and the enzyme cost (ε_1). For δ_1, δ_2, and ε_2, the units are nutrients (amino acids), and for δ_3 and ε_1, the units are nutrients per second.

From Equation 1, an immediate insight can be obtained into the feasibility of the ancestor. Enzymes need to be excreted to obtain nutrients (δ_3), but each enzyme also costs nutrients to produce (ε_1). The difference $\delta_3 - \varepsilon_1$ expresses the net nutrient uptake rate, with a negative value signifying lack of growth. In Figure 2A, $\delta_3 - \varepsilon_1$ is plotted as a function of t_{dig}, the prey digestion time, and f_{mod}, a multiplier that adjusts the base probabilities (P_{enz} and P_{nut}) that an enzyme reaches the prey and a nutrient reaches the predator (Equation S46). The base probabilities of enzyme reaching prey, P_{enz}, and nutrient reaching predator, P_{nut}, are dependent on the geometry of the predator-prey complex, are different in value, and, for P_{enz}, depend on the number of prey on the predator surface (the values are obtained from simulations; Figure S1). A lower f_{mod} means that the ancestor loses more enzymes and nutrients to the environment. Figure 2A reveals that losses of nutrients and enzymes of approximately 75%–99% can be tolerated depending on the digestion time. Confined spaces or proximate kin (to share resources) increase f_{mod} and could allow the ancestor to persist. A longer digestion time, t_{dig}, improves the prospects of the ancestor because each individual enzyme has more time to release amino acids from the prey, reducing enzyme requirement (and loss). Figure 2A suggests that certain niches and lifestyles are inaccessible to the ancestor but are accessible to the phagocytoser (because it does not squander as many enzymes).

Cellular performance of the phagocytoser

The cellular performance of the phagocytoser (Equations 3 and 4) can be compared to that of the ancestor in the same niche. Cellular performance must be calculated in two steps because of the separation of the evolutionary timescale over which the parameter values are optimized by natural selection (first step, Figures 2B and 2C; second step, Figures 2F and 2G). Changing the prey digestion time (t_{dig}) and the prey capture rate (v_{cap}) involves simple, and therefore rapid, regulatory changes in the number of Sec translocases and axopods (e.g., caused by base substitutions that adjust promotor strength). The prey engulfment time (t_{eng}), on the other hand, is decreased during the long process of phagocytosis evolution, in which many new proteins are recruited and optimized. The result is that the value of t_{dig} is always close to optimal in each step along the evolution of phagocytosis (in our scenario), even if the ancestor and phagocytoser differ significantly in the optimal value of t_{dig}. Comparing ancestor and phagocytoser with the same value of t_{dig} is therefore meaningless. The base nutrient return probability (P_{nut}) relates to the nature of the ancestor (and the situation in which it lives) and is therefore not an optimizable parameter.

The phagocytoser outperforms the ancestor for shorter engulfment times, t_{eng}, and lower nutrient return probabilities, P_{nut} (Figures 2F, 2G, and S2). This is true when the cellular performance is calculated from cell division times alone and even more so when the cellular performance accounts for surface area occupancy of the predator plasma membrane. The effect of surface area occupancy is caused by the low area occupancy of phagocytosis (Figures 2D and 2E). Area occupancy is important to consider for large cells because of small surface-to-volume ratios.

Cellular performance of intermediate forms of the phagocytoser

For phagocytosis, the production of a food vacuole and its subsequent fusion are both complex processes that appear to be essential. To test this essentiality, the consequences of a lack of food vacuole fusion were examined in a modified variant of our model. In addition, intermediate forms of phagocytosis were examined that have (1) low selectivity for the food vacuole membrane proteins and (2) less effective prey engulfment.

For phagocytosis in which food vacuoles do not fuse with the plasma membrane (Figure 3A), the food vacuole abundance will reach a steady state. The food vacuoles will not accumulate indefinitely because growth dilutes the remnant food vacuoles. In fact, the steady state volume occupancy of food vacuoles in the cytoplasm is less than 8% (if food vacuoles collapse into a flat shape; Figure 3A). The cell division time is calculated from (Supplemental Information):

$$t_{d,phago} = \frac{\alpha_1 + \alpha_2 + \alpha_3 + \beta_2}{\alpha_5 - \beta_1 - \beta_3} \qquad \text{(Equation 2)}$$

The cell division time depends on quantities representing the base nutrient requirement (α_1), the engulfment cost (α_2), the prey capture cost (α_3), and nutrient value discounting (β_2), all expressed in number of nutrients (amino acids). The net nutrient uptake rate, given by the denominator ($\alpha_5 - \beta_1 - \beta_3$), must be

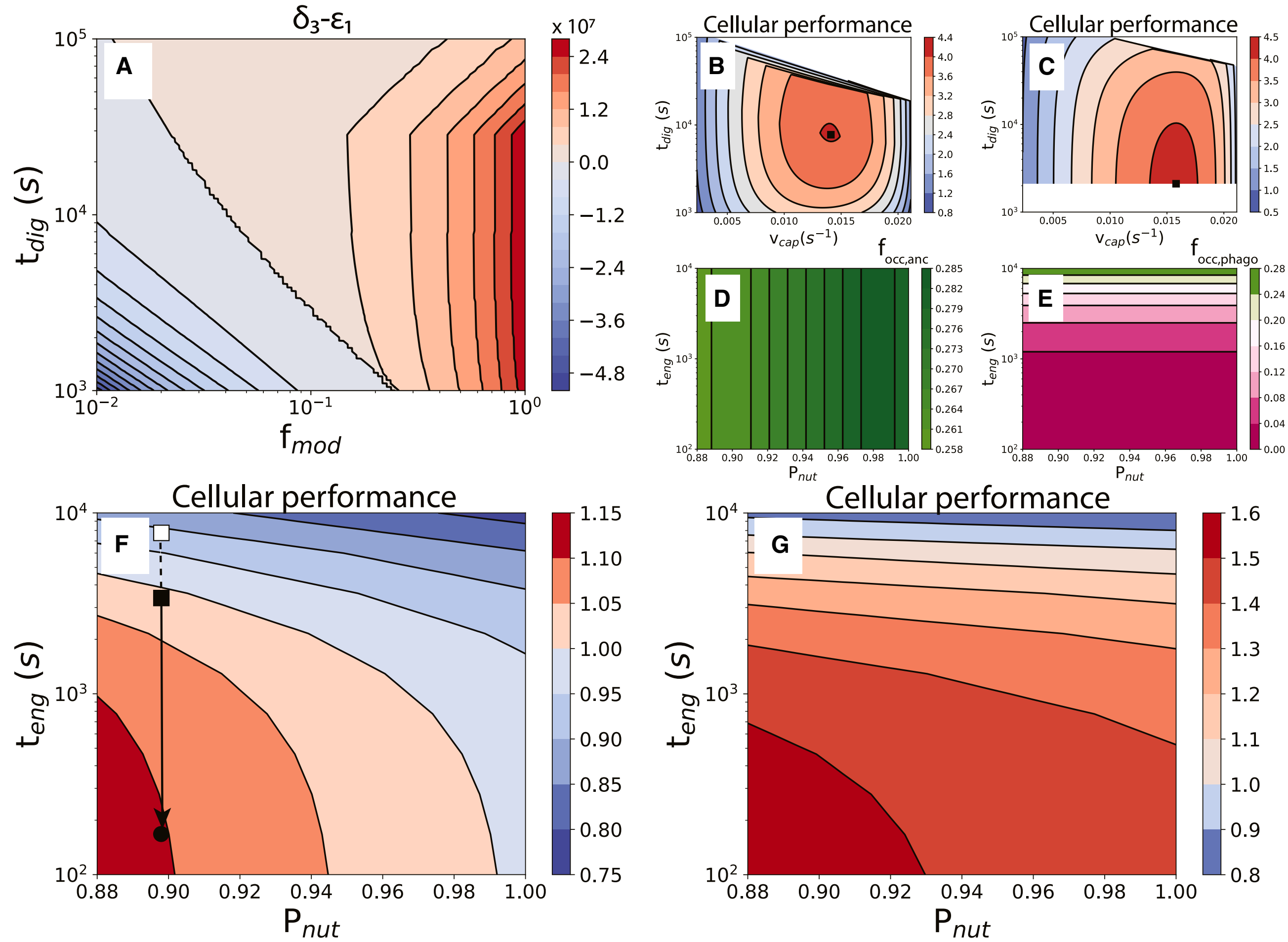

Figure 2. Phagocytosis improves cellular performance

(A) Feasibility of the ancestor. Plot of the net nutrient uptake rate, $\delta_3 - \varepsilon_1$, as a function of the digestion time, t_{dig}, and the modifier, f_{mod}. P_{nut} is 0.88, and P_{enz} is 0.21–1.00, depending on t_{dig}. The sudden reduction in δ_3–ε_1 as t_{dig} increases is caused by the prey occupying the full surface of the predator.

(B) Cellular performance of ancestral states with different digestion times, t_{dig}, and prey capture rates, v_{cap}, compared to one arbitrary ancestral state from the same set. Result for t_{eng} = 167 s and P_{nut} = 0.88. Black square: maximal cellular performance, used for the comparison between the ancestor and the phagocytoser in (G). Cellular performance calculated from Equation 4. White space covers datapoints removed by filters for food vacuole area occupancy (Supplemental Information).

(C) Cellular performance of the phagocytosing states as in (B).

(D) Fractional occupancy of the cell surface by external digestion and prey capture in the ancestor.

(E) Fractional occupancy of the cell surface by engulfment and prey capture in the phagocytoser.

(F) Cellular performance of the phagocytoser compared with the ancestor, as a function of the nutrient return probability, P_{nut}, and the engulfment time, t_{eng}. Calculated from cell division times only (Equation 3). White square: t_{eng} insufficient for the evolution of phagocytosis, Black square: t_{eng} sufficient for the evolution of phagocytosis, Black disc: present day engulfment time.

(G) As in (F) but with cellular performance calculated from cell division times and surface area occupancy (Equation 4). Related to Figure S2.

positive for the phagocytosing organism to grow. This means that the rate of nutrient uptake, α_5, must be larger than the sum of the rate of enzyme production (enzymes are lost upon food vacuole fusion), β_1, and the rate of food vacuole membrane production, β_3 (with all three expressed in nutrient number per second). Strikingly, a quantitative examination of the denominator reveals that food vacuole fusion is not required for the phagocytoser to grow because the denominator is always positive (Figure 3B). A phagocytoser without food vacuole fusion can also outperform the ancestor if fractional surface occupancy is important for cellular performance (Figures 3C and 3D).

Low selectivity for Sec translocases and nutrient transporters into the food vacuole in a primitive phagocytoser would lead to the food vacuole being occupied largely by other proteins (Figure 3E), increasing the digestion time and lowering cellular performance (Figure 2C). Similar consequences result from a high requirement for receptors to bind the prey cell and recruit the actin cytoskeleton in the formation of the phagocytic cup. Figures 3F and 3G reveal that even with only 10% of the food vacuole membrane available for Sec translocases and nutrient transporters, the phagocytoser can outperform the ancestor.

CellPress
OPEN ACCESS

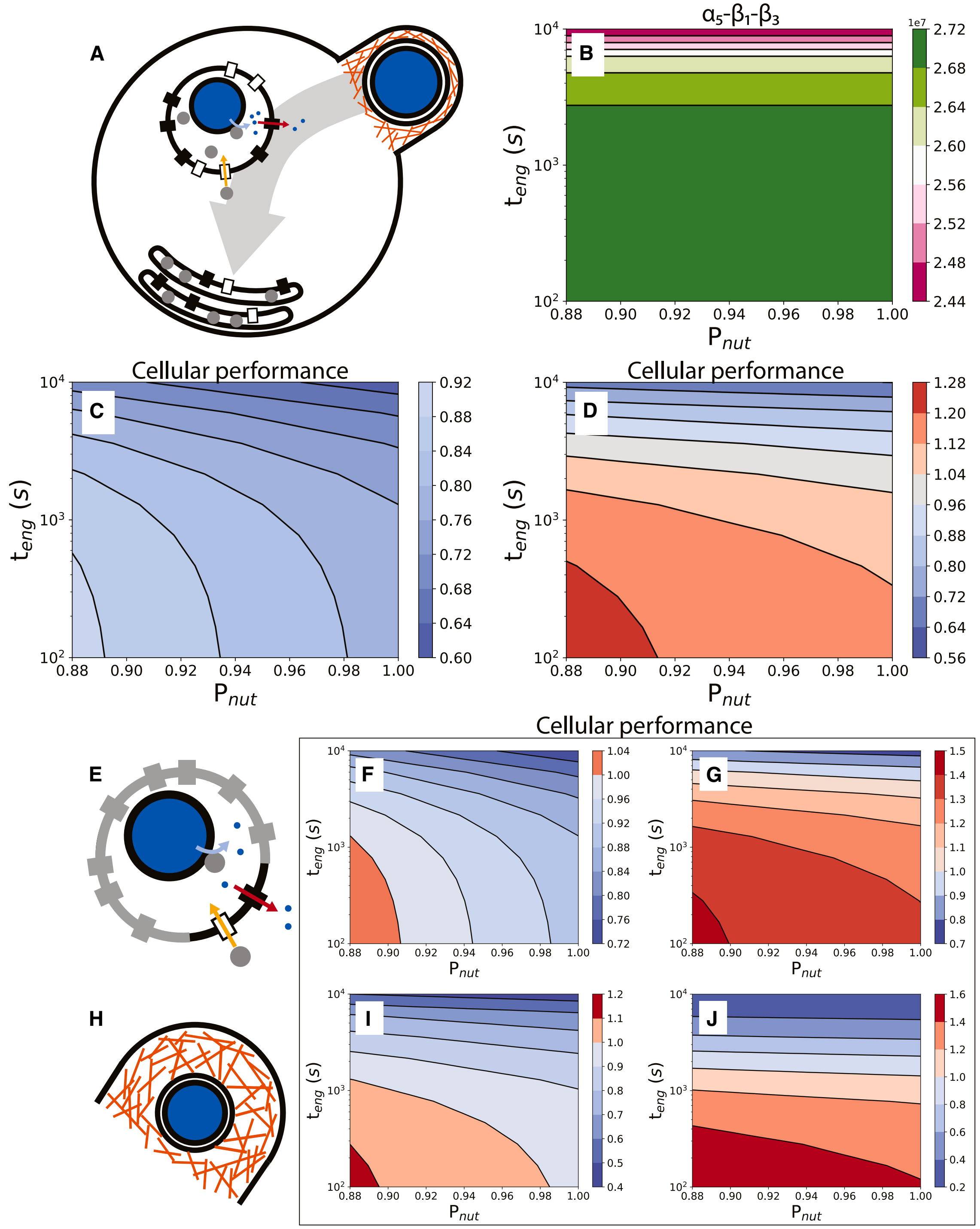

Figure 3. Intermediate forms of phagocytosis improve cellular performance

(A) Schematic of a phagocytoser without food vacuole fusion. Food vacuoles accumulate to a steady state number as flattened membrane compartments.

(B) Feasibility of phagocytosis without food vacuole fusion. Plot of the net nutrient uptake rate, $\alpha_5 - \beta_1 - \beta_3$, as a function of the engulfment time, t_{eng}, and nutrient return probability, P_{nut}.

(C) Cellular performance of the phagocytoser compared with the ancestor in the absence of food vacuole fusion, calculated from cell division times only (Equation 3).

(D) As in (C) but with cellular performance calculated from cell division times and surface area occupancy (Equation 4). (E–G) Cellular performance calculation in which 90% of the food vacuole surface is occupied by other membrane proteins.

(E) Schematic of a food vacuole, with membrane proteins other than Sec translocases and nutrient transporters in gray.

(legend continued on next page)

Prey engulfment will likely be less effective early in the evolution of phagocytosis, which will present itself in longer engulfment time and larger, more expensive, phagocytic cups. Quantification of cellular performance can reveal whether a proposed mechanism of engulfment is sufficient to outperform the ancestor (Figure 2F, black square) or not (Figure 2F, white square), depending on the engulfment time. With subsequent evolution of the engulfment machinery, engulfment time was reduced and cellular performance improved (Figure 2F, arrow and black disc). Modern forms of phagocytosis have engulfment times of minutes,[48,49] similar to that of small-vesicle endocytosis[50] and prey engulfment by dinoflagellates.[51]

Coordination between actin filaments, a product of the many regulatory proteins associated with the actin cytoskeleton, will initially be lacking, potentially leading to large and expensive phagocytic cups (Figure 3H). Cellular performance calculations in which the thickness of the phagocytic cup is increased from 0.11 to 1 μm reveal that the phagocytoser can still outperform the ancestor but only in a reduced parameter range (Figures 3I and 3J).

For understanding gradual evolution of phagocytosis, it is also relevant to examine cells smaller than 1,000 μm^3. This early phagocytoser may not have captured prey by axopods, so we examine a phagocytoser with an undefined prey capture mechanism with a capture rate that we set (Supplemental Information). Predator volumes in the range 10–1,000 μm^3 show similar effectiveness of phagocytosis over the tested volume range (Figures 4A and 4B, and S3). Consequences of prey escape from the ancestor (due to lack of engulfment) and increased digestion efficiency in the phagocytoser (e.g., due to acidification of the food vacuole) were also examined and found improvements in the phagocytoser in roughly linear proportion to loss of prey and increased efficiency (Supplemental Information; Figure S4).

DISCUSSION

A model of phagocytosis is established by which cellular performance is quantified relative to a non-phagocytic ancestor. This model integrates prey capture, engulfment, and external and internal digestion into a single framework, providing a method for judging the biological importance of experimental results that touch on key phagocytosis parameters such as prey engulfment time. The models suggest that phagocytosis can improve cellular performance in the absence of an endomembrane system and that it can do so even when food vacuoles do not fuse with the plasma membrane. Combined with increases in our knowledge of the phagocytosis mechanism, and with the application to additional intermediate states, the model presented here can contribute to our understanding of the macroevolutionary processes that originated phagocytosis and the eukaryotic endomembrane system.

Gradual evolution of phagocytosis

The sequence of cellular transformations by which phagocytosis evolved is obscured by its considerable molecular complexity.[2,32] It may have required an intermediate in which prey was partly engulfed.[28,52,53] A sinking-type phagocytosis as seen in excavates,[54] and perhaps present in the last eukaryotic common ancestor,[55,56] may have been an intermediate to the modern diversified forms of phagocytosis across eukaryote diversity.[24,31] Precursors required for phagocytosis evolution have been found in the Asgard relatives of eukaryotes, including actin cytoskeleton homologues,[57,58] endomembrane regulators,[59–62] and SNAREs,[63] putting an early evolution of phagocytosis in the realm of possibility.

Various studies have revealed how alterations to the mechanism of phagocytosis can affect the engulfment time, a parameter that our theory reveals to be key to phagocytosis success (t_{eng}, Figures 2F and 2G). Mechanistic models suggest that receptor-only phagocytosis, without actin, is possible.[37,64] Knockout of the actin-capping protein CapG reduces the uptake rate of zymosan particles by phagocytosis by roughly 2-fold.[65] Dynamin appears to affect phagocytic cup formation and closure.[66,67] Integrating these results with our phagocytosis model will deepen our understanding of the contribution of the various molecular factors to phagocytosis.

Progress in understanding the evolution of complex traits faces the challenge of the whole trait seemingly having to come into existence all at once with all its components in place. Examples include eyes and wings on the macroscale[68] and ATP synthase[69] and flagella[70] on the molecular and cellular scale. Nevertheless, convincing explanations for the gradual evolution of such complex traits have been proposed.[68,70] A similar difficulty arises in trying to explain the evolution of phagocytosis, and it has been claimed that phagocytosis cannot evolve in the absence of the pre-adaptation of vesicle fusion.[25,28] However, the work presented here suggests that a phagocytoser lacking vacuole fusion can grow, and outperform the ancestor, obviating the need for a vesicle fusion pre-adaptation and rendering a gradual transition more plausible.

Phagocytosis and the endomembrane system

In modern eukaryotes, phagocytosis and other endomembranes always co-occur. The only prokaryote known to perform phagocytosis is a planctomycete that also contains internal membranes.[36] This co-occurrence could mean that phagocytosis is not feasible in the absence of an endomembrane system. The phagocytosis model presented here does not explicitly include other components of the endomembrane system and suggests that phagocytosis could evolve in the absence of such components (e.g., enzyme transport to food vacuoles by vesicle fusion).

It is conceivable that some of the parameter values used in the phagocytosis model depend on the existence of the endomembrane system. Internal digestion in modern phagocytosing

(F) Cellular performance calculated using cell division times only (Equation 3).
(G) Cellular performance calculated using both cell division time and surface area occupation (Equation 4). (H–J) Cellular performance calculation for a phagocytoser with larger phagocytic cups.
(H) Schematic of a larger phagocytic cup.
(I and J) As in (F) and (G).

CellPress
OPEN ACCESS

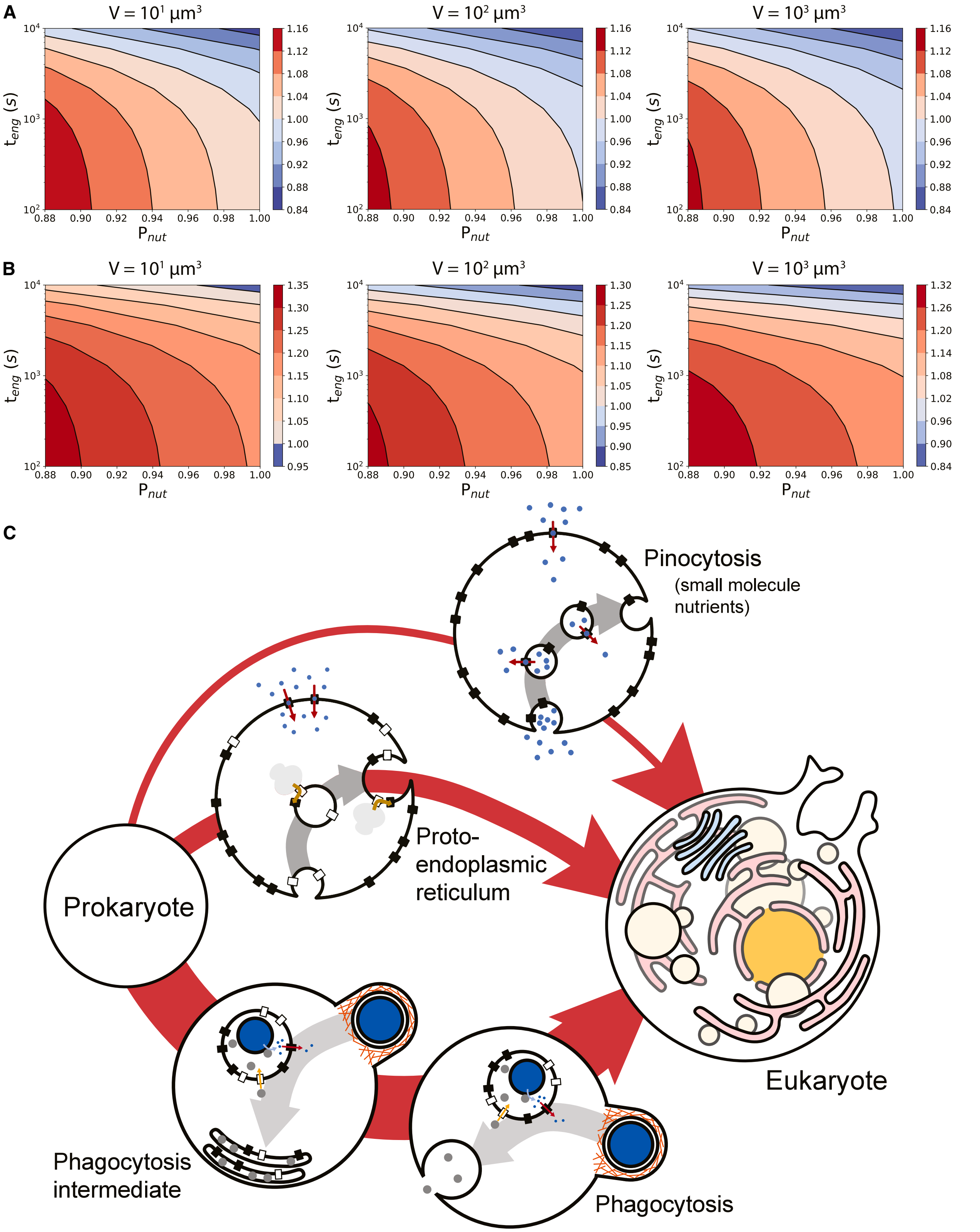

(legend on next page)

species depends on the acidification of the food vacuole lumen by the fusion of endosomes and lysosomes,[2] and it is possible that the rates of prey protein digestion used in the model could only be accomplished in the presence of such acidification, although presumably a food vacuole could also be acidified by V-type ATPases directly.[71] The production (or delivery) of membrane for phagocytosis may also require the endomembrane system.[2,72]

Phagocytosis could have been the starting point of the endomembrane system.[25–29] The model presented here suggests that phagocytosis can exist in niches in which the ancestor cannot and suggests a considerable advantage in cellular performance where they can both survive. This is true for a range of parameter values and intermediate states, including an intermediate in which food vacuoles do not recycle. This suggests that phagocytosis is a more likely candidate for the initiation of endomembrane evolution than pinocytosis (of small nutrient molecules) and the proto-endoplasmic reticulum (Figure 4C).[46] Comparison with other alternative hypotheses of endomembrane evolution[73–78] should be carried out in the future and will depend on quantitative reformulation of those hypotheses.

Limitations of the study

The cellular states examined here, the phagocytoser and its non-phagocytic ancestor, are separated by vast evolutionary time and many genetic alterations. As such, this cannot be considered a complete description of the evolutionary pathway to phagocytosis and endomembranes. Additional intermediates, alternative pathways, and changes in niche will have to be considered for a more complete picture. A further caveat with the present work is the absence of an explicit treatment of survival, for example during periods of starvation, and the lack of a more complete accounting of the ecological situation. The cellular states for which the comparisons in cellular performance were made in this work should be considered as signposts that help us understand the macroevolutionary processes that gave rise to phagocytosis and endomembranes, but do not yet give a satisfactory account of the complete process, lacking a description of how specific mutants can invade a population.

RESOURCE AVAILABILITY

Lead contact

Requests for further information and resources should be directed to and will be fulfilled by the lead contact, Paul E Schavemaker (pschavem@asu.edu).

Materials availability

No materials were generated in this study.

Data and code availability

- This paper analyzes existing, publicly available data, cited in the main text.
- This paper does not report original code.
- Any additional information required to reanalyze the results presented in this paper is available from the lead contact upon request.

ACKNOWLEDGMENTS

This work was funded by the Moore-Simons Project on the Origin of the Eukaryotic Cell, Simons Foundation 735927, the National Institutes of Health, R35-GM122566, and the National Science Foundation, DBI-2119963.

AUTHOR CONTRIBUTIONS

P.E.S., conceptualization, data curation, formal analysis, methodology, validation, visualization, writing – original draft, and writing – review and editing. K.S.T., data curation and formal analysis. M.L., conceptualization, funding acquisition, project administration, supervision, and writing – review and editing.

DECLARATION OF INTERESTS

The authors declare no competing interests.

STAR★METHODS

Detailed methods are provided in the online version of this paper and include the following:

- KEY RESOURCES TABLE
- METHOD DETAILS
  - Calculation of the cellular performance
  - Prey capture
  - Prey external digestion
  - Prey engulfment
  - Prey internal digestion
  - Choice of parameter values
- QUANTIFICATION AND STATISTICAL ANALYSIS

SUPPLEMENTAL INFORMATION

Supplemental information can be found online at https://doi.org/10.1016/j.celrep.2026.118105

Received: February 8, 2026
Revised: July 6, 2026
Accepted: September 21, 2026
Published: October 7, 2026

REFERENCES

1. Flannagan, R.S., Jaumouillé, V., and Grinstein, S. (2012). The cell biology of phagocytosis. Annu. Rev. Pathol. *7*, 61–98. https://doi.org/10.1146/annurev-pathol-011811-132445.
2. Mylvaganam, S., Freeman, S.A., and Grinstein, S. (2021). The cytoskeleton in phagocytosis and macropinocytosis. Curr. Biol. *31*, R619–R632. https://doi.org/10.1016/j.cub.2021.01.036.
3. Hartenstein, V., and Martinez, P. (2019). Phagocytosis in cellular defense and nutrition: a food-centered approach to the evolution of macrophages.

Figure 4. Phagocytosis and the evolution of the endomembrane system
(A) The cellular performance contribution of phagocytosis, accounting only for cell division time (Equations 3, S67, and S68), as a function of predator volume. Prey capture uses a base rate of 0.007 prey per second but is lowered for the smaller predators (Supplemental Information). Related to Figure S3.
(B) The contribution to cellular performance of phagocytosis, accounting for both cell division time and area occupancy (Equations 4 and S67–S70), as a function of predator volume. The prey capture rates are the same as in (A). Related to Figure S3.
(C) Pathways from cells without complex endomembranes to cells with complex endomembranes for which cellular performance calculations have been performed.[46] The likelihood of the evolutionary pathways is proportional to the thickness of the red arrows.

Cell Tissue Res. *377*, 527–547. https://doi.org/10.1007/s00441-019-03096-6.

4. Stossel, T.P. (1999). The early history of phagocytosis. Adv. Cell. Mol. Biol. Membr. Organelles. *5*, 3–18.
5. Leander, B.S. (2020). Predatory protists. Curr. Biol. *30*, R510–R516. https://doi.org/10.1016/j.cub.2020.03.052.
6. Geisen, S., Mitchell, E.A.D., Adl, S., Bonkowski, M., Dunthorn, M., Ekelund, F., Fernández, L.D., Jousset, A., Krashevska, V., Singer, D., et al. (2018). Soil protists: a fertile frontier in soil biology research. FEMS Microbiol. Rev. *42*, 293–323. https://doi.org/10.1093/femsre/fuy006.
7. Dayel, M.J., and King, N. (2014). Prey capture and phagocytosis in the choanoflagellate Salpingoeca rosetta. PLoS One *9*, e95577. https://doi.org/10.1371/journal.pone.0095577.
8. Chrisman, C.J., Alvarez, M., and Casadevall, A. (2010). Phagocytosis of Cryptococcus neoformans by, and nonlytic exocytosis from, Acanthamoeba castellanii. Appl. Environ. Microbiol. *76*, 6056–6062. https://doi.org/10.1128/AEM.00812-10.
9. Sobczak, M., Wasik, A., Kłopocka, W., and Rędowicz, M.J. (2008). Involvement of myosin VI immunoanalog in pinocytosis and phagocytosis in Amoeba proteus. Biochem. Cell. Biol. *86*, 509–519. https://doi.org/10.1139/O08-142.
10. Lewis, K.E., and O'Day, D.H. (1996). Phagocytosis in Dictyostelium: nibbling, eating and cannibalism. J. Eukaryot. Microbiol. *43*, 65–69. https://doi.org/10.1111/j.1550-7408.1996.tb02474.x.
11. Voigt, H., and Guillén, N. (1999). New insights into the role of the cytoskeleton in phagocytosis of Entamoeba histolytica. Cell. Microbiol. *1*, 195–203. https://doi.org/10.1046/j.1462-5822.1999.00021.x.
12. Sakaguchi, M., Murakami, H., and Suzaki, T. (2001). Involvement of a 40-kDa glycoprotein in food recognition, prey capture, and induction of phagocytosis in the protozoon Actinophrys sol. Protist *152*, 33–41. https://doi.org/10.1078/1434-4610-00041.
13. Jacobson, D.M., and Anderson, D.M. (1996). Widespread phagocytosis of ciliates and other protists by marine mixotrophic and heterotrophic thecate dinoflagellates. J. Phycol. *32*, 279–285. https://doi.org/10.1111/j.0022-3646.1996.00279.x.
14. Höhfeld, I., and Melkonian, M. (1998). Lifting the curtain? The microtubular cytoskeleton of Oxyrrhis marina (Dinophyceae) and its rearrangement during phagocytosis. Protist *149*, 75–88. https://doi.org/10.1016/s1434-4610(98)70011-2.
15. McKie-Krisberg, Z.M., and Sanders, R.W. (2014). Phagotrophy by the picoeukaryotic green alga Micromonas: implications for Arctic Oceans. ISME J. *8*, 1953–1961. https://doi.org/10.1038/ismej.2014.16.
16. Maruyama, S., and Kim, E. (2013). A modern descendant of early green algal phagotrophs. Curr. Biol. *23*, 1081–1084. https://doi.org/10.1016/j.cub.2013.04.063.
17. Koester, J.A., Fox, O., Smith, E., Cox, M.B., and Taylor, A.R. (2025). A multifunctional organelle coordinates phagocytosis and chlorophagy in a marine eukaryote phytoplankton Scyphosphaera apsteinii. New Phytol. *246*, 1096–1112. https://doi.org/10.1111/nph.20388.
18. Cavalier-Smith, T., Chao, E.E., and Vickerman, K. (2016). New phagotrophic euglenoid species (new genus Decastava; Scytomonas saepesedens; Entosiphon oblongum), Hsp90 introns, and putative euglenoid Hsp90 pre-mRNA insertional editing. Eur. J. Protistol. *56*, 147–170. https://doi.org/10.1016/j.ejop.2016.08.002.
19. Kusdian, G., Woehle, C., Martin, W.F., and Gould, S.B. (2013). The actin-based machinery of T richomonas vaginalis mediates flagellate-amoeboid transition and migration across host tissue. Cell. Microbiol. *15*, 1707–1721. https://doi.org/10.1111/cmi.12144.
20. Pereira-Neves, A., and Benchimol, M. (2007). Phagocytosis by Trichomonas vaginalis: new insights. Biol. Cell *99*, 87–101. https://doi.org/10.1042/BC20060084.
21. Foissner, W. (1999). Description of two new, mycophagous soil ciliates (Ciliophora, Colpodea): Fungiphrya strobli ng, n. sp. and Grossglockneria ovata n. sp. J. Eukaryot. Microbiol. *46*, 34–42. https://doi.org/10.1111/j.1550-7408.1999.tb04581.x.
22. Verni, F., and Gualtieri, P. (1997). Feeding behaviour in ciliated protists. Micron *28*, 487–504. https://doi.org/10.1016/s0968-4328(97)00028-0.
23. Hausmann, K., and Peck, R.K. (1979). The mode of function of the cytopharyngeal basket of the ciliate Pseudomicrothorax dubius. Differentiation *14*, 147–158. https://doi.org/10.1111/j.1432-0436.1979.tb01023.x.
24. Mills, D.B. (2020). The origin of phagocytosis in Earth history. Interface Focus *10*, 20200019. https://doi.org/10.1098/rsfs.2020.0019.
25. Cavalier-Smith, T. (1975). The origin of nuclei and of eukaryotic cells. Nature *256*, 463–468. https://doi.org/10.1038/256463a0.
26. Cavalier-Smith, T. (1987). The origin of eukaryote and archaebacterial cells. Ann. N. Y. Acad. Sci. *503*, 17–54. https://doi.org/10.1111/j.1749-6632.1987.tb40596.x.
27. Cavalier-Smith, T. (2002). The phagotrophic origin of eukaryotes and phylogenetic classification of Protozoa. Int. J. Syst. Evol. Microbiol. *52*, 297–354. https://doi.org/10.1099/00207713-52-2-297.
28. Cavalier-Smith, T. (2009). Predation and eukaryote cell origins: a coevolutionary perspective. Int. J. Biochem. Cell Biol. *41*, 307–322. https://doi.org/10.1016/j.biocel.2008.10.002.
29. Cavalier-Smith, T. (2014). The neomuran revolution and phagotrophic origin of eukaryotes and cilia in the light of intracellular coevolution and a revised tree of life. Cold Spring Harb. Perspect. Biol. *6*, a016006. https://doi.org/10.1101/cshperspect.a016006.
30. Bremer, N., Tria, F.D.K., Skejo, J., Garg, S.G., and Martin, W.F. (2022). Ancestral state reconstructions trace mitochondria but not phagocytosis to the last eukaryotic common ancestor. Genome Biol. Evol. *14*, evac079. https://doi.org/10.1093/gbe/evac079.
31. Yutin, N., Wolf, M.Y., Wolf, Y.I., and Koonin, E.V. (2009). The origins of phagocytosis and eukaryogenesis. Biol. Direct *4*, 9. https://doi.org/10.1186/1745-6150-4-9.
32. Martin, W.F., Tielens, A.G.M., Mentel, M., Garg, S.G., and Gould, S.B. (2017). The physiology of phagocytosis in the context of mitochondrial origin. Microbiol. Mol. Biol. Rev. *81*, e00008-17–1128. https://doi.org/10.1128/MMBR.00008-17.
33. Koonin, E.V. (2015). Origin of eukaryotes from within archaea, archaeal eukaryome and bursts of gene gain: eukaryogenesis just made easier? Philos. Trans. R. Soc. Lond. B Biol. Sci. *370*, 20140333. https://doi.org/10.1098/rstb.2014.0333.
34. Spang, A., Saw, J.H., Jørgensen, S.L., Zaremba-Niedzwiedzka, K., Martijn, J., Lind, A.E., van Eijk, R., Schleper, C., Guy, L., and Ettema, T.J.G. (2015). Complex archaea that bridge the gap between prokaryotes and eukaryotes. Nature *521*, 173–179. https://doi.org/10.1038/nature14447.
35. Burns, J.A., Pittis, A.A., and Kim, E. (2018). Gene-based predictive models of trophic modes suggest Asgard archaea are not phagocytotic. Nat. Ecol. Evol. *2*, 697–704. https://doi.org/10.1038/s41559-018-0477-7.
36. Shiratori, T., Suzuki, S., Kakizawa, Y., and Ishida, K.I. (2019). Phagocytosis-like cell engulfment by a planctomycete bacterium. Nat. Commun. *10*, 5529. https://doi.org/10.1038/s41467-019-13499-2.
37. Richards, D.M., and Endres, R.G. (2017). How cells engulf: a review of theoretical approaches to phagocytosis. Rep. Prog. Phys. *80*, 126601. https://doi.org/10.1088/1361-6633/aa8730.
38. Lynch, M., and Marinov, G.K. (2015). The bioenergetic costs of a gene. Proc. Natl. Acad. Sci. USA *112*, 15690–15695. https://doi.org/10.1073/pnas.1514974112.
39. Schavemaker, P.E., and Lynch, M. (2025). Bioenergetics and the Evolution of Cellular Traits. Annu. Rev. Biophys. *54*, 81–99. https://doi.org/10.1146/annurev-biophys-070524-090334.
40. Lynch, M., Schavemaker, P.E., Licknack, T.J., Hao, Y., and Pezzano, A. (2022). Evolutionary bioenergetics of ciliates. J. Eukaryot. Microbiol. *69*, e12934. https://doi.org/10.1111/jeu.12934.

41. Raven, J.A., and Richardson, K. (1984). Dinophyte flagella: a cost-benefit analysis. New Phytol. *98*, 259–276. https://doi.org/10.1111/j.1469-8137.1984.tb02736.x.

42. Schavemaker, P.E., and Lynch, M. (2022). Flagellar energy costs across the tree of life. Elife *11*, e77266. https://doi.org/10.7554/eLife.77266.

43. Raven, J.A. (1997). The vacuole: a cost-benefit analysis. Adv. Bot. Res.*25* (Academic Press), pp. 59–86. https://doi.org/10.1016/s0065-2296(08)60148-2.

44. Schavemaker, P.E., and Muñoz-Gómez, S.A. (2022). The role of mitochondrial energetics in the origin and diversification of eukaryotes. Nat. Ecol. Evol. *6*, 1307–1317. https://doi.org/10.1038/s41559-022-01833-9.

45. Lavoie, M., Raven, J.A., and Levasseur, M. (2016). Energy cost and putative benefits of cellular mechanisms modulating buoyancy in aflagellate marine phytoplankton. J. Phycol. *52*, 239–251. https://doi.org/10.1111/jpy.12390.

46. Schavemaker, P.E., and Lynch, M. (2025). Quantifying the evolutionary paths to endomembranes. Cell Rep. *44*, 115533. https://doi.org/10.1016/j.celrep.2025.115533.

47. Driessen, A.J.M., and Nouwen, N. (2008). Protein translocation across the bacterial cytoplasmic membrane. Annu. Rev. Biochem. *77*, 643–667. https://doi.org/10.1146/annurev.biochem.77.061606.160747.

48. Vorselen, D., Barger, S.R., Wang, Y., Cai, W., Theriot, J.A., Gauthier, N.C., and Krendel, M. (2021). Phagocytic 'teeth'and myosin-II 'jaw'power target constriction during phagocytosis. Elife *10*, e68627. https://doi.org/10.7554/eLife.68627.

49. Paul, D., Achouri, S., Yoon, Y.Z., Herre, J., Bryant, C.E., and Cicuta, P. (2013). Phagocytosis dynamics depends on target shape. Biophys. J. *105*, 1143–1150. https://doi.org/10.1016/j.bpj.2013.07.036.

50. Sirotkin, V., Berro, J., Macmillan, K., Zhao, L., and Pollard, T.D. (2010). Quantitative analysis of the mechanism of endocytic actin patch assembly and disassembly in fission yeast. Mol. Biol. Cell *21*, 2894–2904. https://doi.org/10.1091/mbc.E10-02-0157.

51. Hansen, P.J., and Calado, A.J. (1999). Phagotrophic Mechanisms and Prey Selection in Free-living Dinoflagellates. J. Eukaryot. Microbiol. *46*, 382–389. https://doi.org/10.1111/j.1550-7408.1999.tb04617.x.

52. Kamennaya, N.A., Kennaway, G., Fuchs, B.M., and Zubkov, M.V. (2018). "Pomacytosis"—Semi-extracellular phagocytosis of cyanobacteria by the smallest marine algae. PLoS Biol. *16*, e2003502. https://doi.org/10.1371/journal.pbio.2003502.

53. Maxfield, F.R., Barbosa-Lorenzi, V.C., and Singh, R.K. (2020). Digestive exophagy: Phagocyte digestion of objects too large for phagocytosis. Traffic *21*, 6–12. https://doi.org/10.1111/tra.12712.

54. Yubuki, N., Simpson, A.G.B., and Leander, B.S. (2013). Comprehensive ultrastructure of Kipferlia bialata provides evidence for character evolution within the Fornicata (Excavata). Protist *164*, 423–439. https://doi.org/10.1016/j.protis.2013.02.002.

55. Williamson, K., Eme, L., Baños, H., McCarthy, C.G.P., Susko, E., Kamikawa, R., Orr, R.J.S., Muñoz-Gómez, S.A., Minh, B.Q., Simpson, A.G.B., et al. (2025). A robustly rooted tree of eukaryotes reveals their excavate ancestry. Nature *640*, 974–981. https://doi.org/10.1038/s41586-025-08709-5.

56. Suzuki-Tellier, S., Miano, F., Asadzadeh, S.S., Simpson, A.G.B., and Kiørboe, T. (2024). Foraging mechanisms in excavate flagellates shed light on the functional ecology of early eukaryotes. Proc. Natl. Acad. Sci. USA *121*, e2317264121. https://doi.org/10.1073/pnas.2317264121.

57. Akıl, C., and Robinson, R.C. (2018). Genomes of Asgard archaea encode profilins that regulate actin. Nature *562*, 439–443. https://doi.org/10.1038/s41586-018-0548-6.

58. Rodrigues-Oliveira, T., Wollweber, F., Ponce-Toledo, R.I., Xu, J., Rittmann, S.K.M.R., Klingl, A., Pilhofer, M., and Schleper, C. (2023). Actin cytoskeleton and complex cell architecture in an Asgard archaeon. Nature *613*, 332–339. https://doi.org/10.1038/s41586-022-05550-y.

59. Tran, L.T., Akl, C., Senju, Y., and Robinson, R.C. (2024). The eukaryotic-like characteristics of small GTPase, roadblock and TRAPPC3 proteins from Asgard archaea. Commun. Biol. *7*, 273.

60. Vargová, R., Chevreau, R., Alves, M., Courbin, C., Terry, K., Legrand, P., Eliáš, M., Ménétrey, J., Dacks, J.B., and Jackson, C.L. (2025). The Asgard archaeal origins of Arf family GTPases involved in eukaryotic organelle dynamics. Nat. Microbiol. *10*, 495–508. https://doi.org/10.1038/s41564-024-01904-6.

61. Klinger, C.M., Spang, A., Dacks, J.B., and Ettema, T.J.G. (2016). Tracing the archaeal origins of eukaryotic membrane-trafficking system building blocks. Mol. Biol. Evol. *33*, 1528–1541. https://doi.org/10.1093/molbev/msw034.

62. Novák Vanclová, A.M.G., Jackson, C.L., and Dacks, J.B. (2025). Eukaryote-wide distribution of a family of longin domain-containing GAP complexes for small GTPases. Traffic *26*, e70016. https://doi.org/10.1111/tra.70016.

63. Neveu, E., Khalifeh, D., Salamin, N., and Fasshauer, D. (2020). Prototypic SNARE proteins are encoded in the genomes of Heimdallarchaeota, potentially bridging the gap between the prokaryotes and eukaryotes. Curr. Biol. *30*, 2468–2480.e5. https://doi.org/10.1016/j.cub.2020.04.060.

64. Tollis, S., Dart, A.E., Tzircotis, G., and Endres, R.G. (2010). The zipper mechanism in phagocytosis: energetic requirements and variability in phagocytic cup shape. BMC Syst. Biol. *4*, 149. https://doi.org/10.1186/1752-0509-4-149.

65. Witke, W., Li, W., Kwiatkowski, D.J., and Southwick, F.S. (2001). Comparisons of CapG and gelsolin-null macrophages: demonstration of a unique role for CapG in receptor-mediated ruffling, phagocytosis, and vesicle rocketing. J. Cell Biol. *154*, 775–784. https://doi.org/10.1083/jcb.200101113.

66. Marie-Anaïs, F., Mazzolini, J., Herit, F., and Niedergang, F. (2016). Dynamin-actin cross talk contributes to phagosome formation and closure. Traffic *17*, 487–499. https://doi.org/10.1111/tra.12386.

67. Gold, E.S., Underhill, D.M., Morrissette, N.S., Guo, J., McNiven, M.A., and Aderem, A. (1999). Dynamin 2 is required for phagocytosis in macrophages. J. Exp. Med. *190*, 1849–1856. https://doi.org/10.1084/jem.190.12.1849.

68. Dawkins, R. (1996). Climbing Mount Improbable (WW Norton & Company).

69. Walker, J.E. (2013). The ATP synthase: the understood, the uncertain and the unknown. Biochem. Soc. Trans. *41*, 1–16. https://doi.org/10.1042/BST20110773.

70. Beeby, M., Ferreira, J.L., Tripp, P., Albers, S.-V., and Mitchell, D.R. (2020). Propulsive nanomachines: the convergent evolution of archaella, flagella and cilia. FEMS Microbiol. Rev. *44*, 253–304. https://doi.org/10.1093/femsre/fuaa006.

71. Kane, P.M. (2006). The where, when, and how of organelle acidification by the yeast vacuolar H+-ATPase. Microbiol. Mol. Biol. Rev. *70*, 177–191. https://doi.org/10.1128/MMBR.70.1.177-191.2006.

72. Suzaki, E., Kobayashi, H., Kodama, Y., Masujima, T., and Terakawa, S. (1997). Video-rate dynamics of exocytotic events associated with phagocytosis in neutrophils. Cell Motil Cytoskeleton *38*, 215–228. https://doi.org/10.1002/(SICI)1097-0169(1997)38:3<215::AID-CM1>3.0.CO;2-4.

73. Jékely, G. (2003). Small GTPases and the evolution of the eukaryotic cell. Bioessays *25*, 1129–1138. https://doi.org/10.1002/bies.10353.

74. Jekely, G. (2007). Origin of eukaryotic endomembranes: a critical evaluation of different model scenarios. Eukaryotic membranes and Cytoskeleton: Origins and Evolution, 38–51. https://doi.org/10.1007/978-0-387-74021-8_3.

75. Gould, S.B., Garg, S.G., and Martin, W.F. (2016). Bacterial vesicle secretion and the evolutionary origin of the eukaryotic endomembrane system. Trends Microbiol. *24*, 525–534. https://doi.org/10.1016/j.tim.2016.03.005.

76. Baum, D.A., and Baum, B. (2014). An inside-out origin for the eukaryotic cell. BMC Biol. *12*, 76. https://doi.org/10.1186/s12915-014-0076-2.

77. De Duve, C. (2007). The origin of eukaryotes: a reappraisal. Nat. Rev. Genet. *8*, 395–403. https://doi.org/10.1038/nrg2071.

78. Blobel, G. (1980). Intracellular protein topogenesis. Proc. Natl. Acad. Sci. USA *77*, 1496–1500. https://doi.org/10.1073/pnas.77.3.1496.

79. Hunter, J.D. (2007). Matplotlib: A 2D graphics environment. Comput. Sci. Eng. *9*, 90–95. https://doi.org/10.1109/mcse.2007.55.

80. Andrews, S.S., Addy, N.J., Brent, R., and Arkin, A.P. (2010). Detailed simulations of cell biology with Smoldyn 2.1. PLoS Comput. Biol. *6*, e1000705. https://doi.org/10.1371/journal.pcbi.1000705.

81. Gast, R.J. (2017). Centrohelida and other heliozoan-like protists. Handbook of the Protists *2*, 955–971.

82. Yabuki, A., Chao, E.E., Ishida, K.-I., and Cavalier-Smith, T. (2012). Microheliella maris (Microhelida ord. n.), an ultrastructurally highly distinctive new axopodial protist species and genus, and the unity of phylum Heliozoa. Protist *163*, 356–388. https://doi.org/10.1016/j.protis.2011.10.001.

83. Jakobsen, H.H., and Hansen, P.J. (1997). Prey size selection, grazing and growth response of the small heterotrophic dinoflagellate Gymnodinium sp. and the ciliate Balanion comatum–a comparative study. Mar. Ecol. Prog. Ser. *158*, 75–86. https://doi.org/10.3354/meps158075.

84. Schavemaker, P.E., and Poolman, B. (2018). (Membrane) Protein Production in Context. Trends Biochem. Sci. *43*, 858–868. https://doi.org/10.1016/j.tibs.2018.08.009.

85. Chang, A., Jeske, L., Ulbrich, S., Hofmann, J., Koblitz, J., Schomburg, I., Neumann-Schaal, M., Jahn, D., and Schomburg, D. (2021). BRENDA, the ELIXIR core data resource in 2021: new developments and updates. Nucleic Acids Res. *49*, D498–D508. https://doi.org/10.1093/nar/gkaa1025.

86. Leadbeater, B.S.C., and Morton, C. (1974). A microscopical study of a marine species of Codosiga James-Clark (Choanoflagellata) with special reference to the ingestion of bacteria. Biol. J. Linn. Soc. Lond. *6*, 337–347. https://doi.org/10.1111/j.1095-8312.1974.tb00728.x.

87. Medalia, O., Beck, M., Ecke, M., Weber, I., Neujahr, R., Baumeister, W., and Gerisch, G. (2007). Organization of actin networks in intact filopodia. Curr. Biol. *17*, 79–84. https://doi.org/10.1016/j.cub.2006.11.022.

88. Abraham, V.C., Krishnamurthi, V., Taylor, D.L., and Lanni, F. (1999). The actin-based nanomachine at the leading edge of migrating cells. Biophys. J. *77*, 1721–1732. https://doi.org/10.1016/S0006-3495(99)77018-9.

89. Mund, M., van der Beek, J.A., Deschamps, J., Dmitrieff, S., Hoess, P., Monster, J.L., Picco, A., Nédélec, F., Kaksonen, M., and Ries, J. (2018). Systematic nanoscale analysis of endocytosis links efficient vesicle formation to patterned actin nucleation. Cell *174*, 884–896.e17. https://doi.org/10.1016/j.cell.2018.06.032.

90. Kasza, K.E., Broedersz, C.P., Koenderink, G.H., Lin, Y.C., Messner, W., Millman, E.A., Nakamura, F., Stossel, T.P., Mackintosh, F.C., and Weitz, D.A. (2010). Actin filament length tunes elasticity of flexibly cross-linked actin networks. Biophys. J. *99*, 1091–1100. https://doi.org/10.1016/j.bpj.2010.06.025.

91. Hanson, J., and Lowy, J. (1963). The structure of F-actin and of actin filaments isolated from muscle. J. Mol. Biol. *6*, 46. https://doi.org/10.1016/s0022-2836(63)80081-9.

92. Müller, J.B., Geyer, P.E., Colaço, A.R., Treit, P.V., Strauss, M.T., Oroshi, M., Doll, S., Virreira Winter, S., Bader, J.M., Köhler, N., et al. (2020). The proteome landscape of the kingdoms of life. Nature *582*, 592–596. https://doi.org/10.1038/s41586-020-2402-x.

93. Eichinger, L., Pachebat, J.A., Glöckner, G., Rajandream, M.A., Sucgang, R., Berriman, M., Song, J., Olsen, R., Szafranski, K., Xu, Q., et al. (2005). The genome of the social amoeba Dictyostelium discoideum. Nature *435*, 43–57. https://doi.org/10.1038/nature03481.

94. Lynch, M., and Marinov, G.K. (2017). Membranes, energetics, and evolution across the prokaryote-eukaryote divide. Elife *6*, e20437. https://doi.org/10.7554/eLife.20437.

STAR★METHODS

KEY RESOURCES TABLE

REAGENT or RESOURCE	SOURCE	IDENTIFIER
Software and algorithms		
Wolfram Mathematica	Wolfram Research	https://www.wolfram.com/mathematica/
Python 3.8.2	Python Software Foundation	https://www.python.org/
Matplotlib	Hunter et al.[79]	https://matplotlib.org/
Smoldyn	Andrews et al.[80]	https://www.smoldyn.org/

METHOD DETAILS

Calculation of the cellular performance

Cellular performance relates to the growth and survival of an organism and is here defined in relative terms, comparing two cellular states. The formulation of cellular performance used here mostly reflects cell division time but, in some instances, includes survival implicitly. The calculations also assume exponential growth without a stationary period, ignore the energetic cost of operating the cellular traits (only including construction cost, see below), and assume a relatively stable ecological niche. Invasion of specific mutations into a population is not examined as the timescales, and number of genetic alterations, between the examined cellular states are large. As such, the model doesn't present evolutionary dynamics but simply examines the relative performance of a sequence of cellular states and determines the conditions under which these cellular states are open or closed to evolution. I.e. cellular states lower in performance than ancestral states are unlikely to be attained by evolution whereas cellular states higher in performance may be attained by evolution but only if the underlying evolutionary dynamics, of mutation and fixation, allows it.

Cellular performance is approximated by two methods (Supplemental Information) that abstract away the details of cellular functioning to serve as a framework for future, more detailed and comprehensive, examinations of the contribution of phagocytosis to cellular performance. For both methods, we examine growth rate, which is only one aspect of the cellular performance. The first method is a simple ratio of cell division times,[46] in which we assume that other aspects of cellular performance, i.e., survival, are equal between phagocytoser and ancestor (Supplemental Information):

$$\textit{Cellular performance} = \frac{t_{d,anc}}{t_{d,phago}} \quad \text{(Equation 3)}$$

Where $t_{d,anc}$ and $t_{d,phago}$ are the cell division times of the ancestor and phagocytoser, respectively. Equation 3 can be applied when the cell surface area is not limiting for growth and survival. In large cells, which have small area-to-volume ratios, the surface area of the cell is a scarce resource and occupying it will come with a steep cellular performance penalty. To account for this effect (previously included in the cell division time[46]), a second method to calculate cellular performance is used:

$$\textit{Cellular performance} = \frac{t_{d,anc}}{t_{d,phago}} \frac{\left(1 - f_{occ,phago}\right)}{\left(1 - f_{occ,anc}\right)} \quad \text{(Equation 4)}$$

Here, $f_{occ,\,phago}$ is the fractional occupancy of the plasma membrane by the phagocytosis and prey capture machinery in the phagocytoser and $f_{occ,anc}$ is the equivalent for the ancestor. The surface of the cell is essential for its growth and survival, so a fractional decrease of available surface area is likely to have a proportional fractional effect on the cellular performance. Other relationships between surface area occupancy and the impact on cellular performance are conceivable and will have to be examined in the future. Unlike the first method, including surface area occupancy can include the effects of survival, for example if proteins important for survival (e.g., for osmoprotection or toxin expulsion) are displaced from the surface area. Note that the outputs of Equations 3 and 4 resemble fitness but that the invasion and fixation of individual mutants in a population is not examined, so the term cellular performance is used instead.

The cellular states that are examined here would never encounter one another in the same environment, because they are likely to exist millions of years apart. The cellular performance calculations compare how well each cellular state would do in a specific set of environments. This is relevant to an evolutionary scenario for the large-scale change of cellular features. If we were to find that there would be no environments in which phagocytosis could function better than the ancestor, this would constitute evidence against phagocytosis as an intermediate toward the endomembrane system. Finding that phagocytosis does improve the prospects of a cell in a specific set of environments also suggests that intermediates between the phagocytoser and ancestor have a cellular performance somewhere in between that of these states and would improve upon the ancestor as well, with the caveat that there may be bottlenecks along the way that need to be examined in the future and that the process of mutations invading a population is not examined here.

Cell division time is a key contributor to cellular performance, even in species that grow relatively slowly (like phagotrophic eukaryotes compared to some bacterial species like *Escherichia coli*). This is because growth rate is a consequence of a specific niche and, even when slow, could be a main contributor simply because it is difficult to grow fast in this environment and with this lifestyle. This may be true for phagocytosis, where the fact that nutrients first need to be processed, rather than used directly as soon as they are imported (as in some bacteria), and the requirement to be large, impose fundamental limitations on growth rate.

Under the assumption of nutrient limited growth, the cell division time is the ratio of the nutrient requirement for producing a new cell and the nutrient acquisition rate[46]:

$$t_d = \frac{Nutrient\ requirement}{Nutrient\ acquisition\ rate} \quad \text{(Equation 5)}$$

For the ancestor, this becomes (see Supplemental Information):

$$t_{d,anc} = \frac{N_{nutV} V_{pred} \left(1+C_{trait,anc}\right)}{N_{AA,prey} P_{nut} v_{capadj} N_{value,anc}} \quad \text{(Equation 6)}$$

Here, N_{nutV} is the nutrient requirement per unit of predator volume, V_{pred} is the predator volume, $C_{trait,anc}$ is the relative energetic cost of prey capture and external digestion in the ancestor, $N_{AA,prey}$ is the number of nutrients (amino acids) present in a prey cell, P_{nut} is the base probability that a nutrient produced by external digestion reaches the predator, v_{capadj} is the prey capture rate adjusted for cell growth (see Supplemental Information, calculated from the prey capture rate v_{cap}), and $N_{value,anc}$ is the average nutrient value in the ancestor that accounts for a delay in nutrient return to the predator (see Supplemental Information). As an example of loss of nutrient value, consider a batch of nutrient molecules that takes a whole cell division period to return to the predator. It will lose half its value as it will need to supply two cells instead of one.

For the phagocytoser the cell division time becomes:

$$t_{d,phago} = \frac{N_{nutV} V_{pred} \left(1+C_{trait,phago}\right)}{N_{AA,prey} v_{capadj} N_{value,phago}} \quad \text{(Equation 7)}$$

This does not include P_{nut} because the prey is digested internally and no nutrients are lost. $C_{trait,\ phago}$ includes prey capture, engulfment, and internal digestion costs.

The cellular performance calculation involves two steps and overall, four parameters are varied: the prey digestion time (t_{dig}), the prey capture rate (v_{cap}), the prey engulfment time in the phagocytoser (t_{eng}), and the nutrient return probability (P_{nut}). The first step in the cellular performance calculation is a comparison of ancestor-to-ancestor, and phagocytoser-to-phagocytoser, in which the parameter values of t_{dig} and v_{cap} are varied (Figures 2B and 2C), and t_{eng} and P_{nut} are kept constant. One arbitrary combination of t_{dig} and v_{cap} is chosen as a reference point to which all others are compared. The digestion time optimum found for the phagocytoser, 35 min, is consistent with values reported for ciliates (20 min to 1 h).[22] In the second step, the parameter values associated with the optima from the first step (Figures 2B and 2C, black squares) are used to calculate the cellular performance of the phagocytoser relative to the ancestor. This two-step cellular performance calculation is repeated for a range of engulfment times, t_{eng}, and nutrient-return probabilities, P_{nut} (Figures 2F and 2G).

Prey capture

For prey capture, the heliozoan mechanism was mainly used because it allows for the calculation of the relation between energetic cost, cell surface occupancy, and prey capture rate, which are all important for the cellular performance calculation. For other prey capture mechanisms, such as in ciliates and amoebae, these relationships require more sophisticated models not available at present. The heliozoan prey capture mechanism is mechanistically relatively simple and has evolved multiple times,[81] suggesting that it is easy to evolve. Combined with the fact that the Asgard archaeal relatives of eukaryotes have membranous protrusion supported by cytoskeletal filaments,[58] a feature also present in heliozoa, a heliozoan-like prey capture mechanism can't be ruled out as the primitive prey capture mechanism, even though every presently known case has evolved after the diversification of eukaryotes.

Heliozoan prey capture and subsequent phagocystosis had been observed for *Actinophrys sol* by Albert von Koelliker[4] in 1848. It involves long cellular protrusions called axopods that capture prey passively and transport it to the predator cell surface to be phagocytosed. Axopods consist of a lipid membrane that is extended out from the plasma membrane by a bundle of microtubules. Axopods can capture a variety of prey of different sizes, including bacteria.[82] Axopod properties, used to calculate energy cost and area occupancy, were extracted from the literature (Figures S1A and S1B; Tables S1 and S2).

Prey capture in the model is based on prey cell diffusion and was quantitatively examined by simulations in the software Smoldyn[80] (see Supplemental Information, Figure 1C), yielding the relation between the number of axopods, N_{axo}, and the prey capture rate, v_{cap} (Figure 1D):

$$v_{cap} = \frac{A c_{prey} N_{axo}}{B+N_{axo}} \quad \text{(Equation 8)}$$

This is an empirical fit to the data and has no theoretical justification, but it is similar to a Michaelis-Menten equation for enzyme kinetics. Here, A and B are fit parameters and c_{prey} is the prey concentration. The number of axopods can be related to the axopod energy cost and axopod area occupancy (see Supplemental Information). As the prey concentration is not varied in this study, we haven't examined saturation of prey uptake rate with prey concentration,[83] and it is assumed that prey concentration is non-saturating.

Prey external digestion

In the ancestor, prey is digested externally by enzymes secreted, at a known rate,[84] from plasma membrane-localized protein exporters, called Sec translocases,[47] all over the predator cell surface. The base probability with which these enzymes find the prey by random diffusion, P_{enz}, is determined by Smoldyn simulations[80] as a function of the prey number that is present on the predator surface simultaneously (Supplemental Information). When only one prey cell is present $P_{enz} = 0.067$ (Figure S1C), but this can go up to 1 for larger prey numbers (Figure S1E). Enzymes are assumed to have anchors keeping them attached to the prey, similar to anchoring of enzymes to the predator cell surface.[28] At the prey, the enzymes produce nutrients (amino acids) that reach the predator with a base probability, P_{nut}, of 0.88 (determined by Smoldyn simulation, Figure S1D, Supplemental Information). For simplicity, the prey is assumed to consist entirely of protein, with the rate of amino acid production estimated from digestion rates found in the BRENDA database[85] (Figure S1F; Table S3). The energy cost of external digestion depends on the number of excreted enzymes, and the area occupancy depends on the number of Sec translocases and nutrient transporters (Supplemental Information). To investigate the limits under which the ancestor can survive, the base numbers for the enzyme to prey probability (P_{enz}) and the nutrient return probability (P_{nut}), as calculated above, are multiplied by a factor, f_{mod} (Equation S46). The factor f_{mod} is introduced to adjust P_{enz} and P_{nut} simultaneously, as both may depend similarly on fluid flow, confinement, and prey-predator distance. This can't be accomplished by putting P_{enz} equal to P_{nut} because they have different base values, and because P_{enz} depends on the number of prey cells being digested simultaneously while P_{nut} doesn't.

Prey engulfment

To facilitate estimating the size of the engulfment machinery, and thereby its energetic cost and area occupancy, only protrusion-based engulfment, in which a phagocytic cup emerges from the surface of the predator cell,[48,86] is examined here (Supplemental Information). The energetic cost of engulfment is the sum of the membrane cost and the phagocytic cup plasm cost, with the plasm cost residing in actin filaments[87–91] and actin accessory proteins (myosin, arp2/3, etc.).[50,92,93] In the absence of a quantitative description of the actin cytoskeleton of the phagocytic cup, data were acquired from other membrane structures shaped by the actin cytoskeleton: filopodia,[87] lamellipodia,[88] and endocytic vesicles.[89] This information is combined with energetic cost of membranes[42,94] and with the geometry and size of phagocytic cups from mouse macrophage[48] and the choanoflagellate *Codosiga*.[86] In addition to phagocytosis, the energy cost of an endocytic vesicle from *Schizosaccharomyces pombe*[46,50] was included in the analysis. The energetic costs calculated here contain only construction costs, operating costs are not accounted for and are expected to contribute only to a minor degree (but this needs to be examined in the future). The energy cost of engulfment is shown in Figure 1E. The cost per unit of engulfed volume (Figure 1F) reveals that larger cups are cheaper per unit of internalized volume. This is due to a smaller area-to-volume ratio and potentially makes larger cups easier to evolve than smaller cups and explaining why phagocytic cups are largely absent from small cells.

Prey internal digestion

The product of engulfment is a food vacuole, consisting of a membrane surrounding a prey cell. The food vacuole membrane houses Sec translocases, to import enzymes into the food vacuole lumen, and nutrient transporters, to transport amino acids from the vacuole lumen into the cytoplasm. After the prey is digested, the food vacuole membrane fuses back to the predator plasma membrane and the enzymes, present in the food vacuole lumen, are lost to the external medium. Internal digestion is treated similarly to external digestion except that all the nutrients are recovered and all enzymes make it to the prey (Supplemental Information).

Choice of parameter values

Where possible, the model is supported by parameter values extracted from experimental work, simulations, and theoretical analyses. Parameter values used to obtain the results are listed with their sources in Table S4.

QUANTIFICATION AND STATISTICAL ANALYSIS

There are no quantification or statistical analyses to include in this study.

Cell Reports, Volume *45*

Supplemental information

Quantifying the energetic costs and growth benefits of phagocytosis

Paul E. Schavemaker, Kaitlyn S. Tam, and Michael Lynch

Methods S1: Calculating cellular performance of the phagocytosing state, related to Figure 2

The cellular performance of a primitive phagocytoser that processes prey internally, is compared with an ancestor that processes prey externally. Building on previous work on pinocytosis and proto-endoplasmic reticulum[1] the cellular performance of a phagocytoser is defined as:

$$Cellular\ performance = \frac{t_{d,anc}}{t_{d,phago}} \qquad (S1)$$

Here, $t_{d,anc}$ is the cell division time of the ancestor and $t_{d,phago}$ the cell division time of the phagocytoser. Here it is assumed that survival ability remains the same between ancestor and phagocytoser. It is also assumed that the main contributor to cellular performance is the growth rate in the exponential phase. Both survival and growth rate (and efficiency) during nutrient depletion need to be accounted for in the future for a full analysis of the cellular performance changes caused by phagocytosis.

In previous work it was possible to include the effect of area occupancy on cellular performance into the cell division time calculation[1]. Here, the area effect is accounted for heuristically by modifying the ratio of cell division times by a comparison of fractional occupancies of the predator surface area in the phagocytoser, $f_{occ,phago}$, and the ancestor, $f_{occ,anc}$:

$$Fitness = \frac{t_{d,anc}}{t_{d,phago}} \frac{\left(1 - f_{occ,phago}\right)}{\left(1 - f_{occ,anc}\right)} \qquad (S2)$$

The logic behind this modification is that the membrane is an essential bottleneck to the functioning of cells, especially when the predator cells are large, and it is expected that (at least for small fractional occupancies) the negative effect on cellular performance is linearly proportional to the fractional occupancy. More sophisticated interactions between the fractional occupancies, cell division times, and cellular performance, are possible but are left to future work.

The cell division times, $t_{d,anc}$ and $t_{d,phago}$, are calculated as a ratio of nutrient requirement and the nutrient acquisition rate, under the assumption that the nutrient acquisition rate is limiting for the growth rate:

$$t_d = \frac{Nutrient\ requirement}{Nutrient\ acquisition\ rate} \qquad (S3)$$

The main driver of predator growth rate is prey capture rate. The difference between the phagocytoser and its externally digesting ancestor is the result of a second-tier effect: the efficiency of processing the prey after it has been captured. This efficiency has three components: (1) loss of nutrients and enzymes, (2) energy cost of processing (excluding enzyme loss), and (3) surface area occupancy of prey processing. The loss of nutrients and enzymes affects nutrient acquisition rate, the energy cost of processing affects the nutrient requirement, and the surface area occupancy (in the present model) affects cellular performance outside of the cell division time.

Methods S2: Prey capture (ancestor and phagocytoser), related to Figure 1 and 2

One of the key parameters that sets the cell division time is the rate of prey capture. In principle we can simply vary the prey capture time and calculate the cellular performance of the phagocytoser relative to the ancestor purely on differences in prey processing. The reason for including prey capture explicitly is that there is a potential interaction between prey capture and prey processing via the predator cell surface area. A larger area occupancy for prey processing by external digestion compared to by engulfment and internal digestion can affect how much area is available for prey capture. It may also be of interest in the future for comparisons to other ancestors that differ in prey capture mechanism or that don't obtain nutrition from prey at all.

Modeling the prey capture rate gives the relation between prey capture rate, energetic cost of the prey capture mechanism, and the area occupancy of the prey capture mechanism. In practice, we vary the prey capture rate within a biologically plausible range and calculate the energetic cost and area occupancy form the prey capture rate.

Prey capture mechanisms for single-celled organisms are extremely varied and presumably would have different relationships between capture rate, energetic cost, and area occupancy. The relationship between these quantities can be modeled relatively easily for the heliozoan prey capture mechanism.

Heliozoan prey capture simulation

Heliozoa are spherical cells covered in radially extending stiff appendages called axopods. Prey cells swim or diffuse into these axopods and get stuck. Subsequently they are transported to the base of the axopods and are phagocytosed. Here, a heliozoan geometry is set up in the reaction-diffusion simulation software Smoldyn[2] and prey cells are produced at a fixed rate per area on a bounding sphere (concentric with the heliozoan cell) with a radius of 200 μm. These prey cells are allowed to diffuse with a diffusion coefficient of 0.396 $\mu m^2/s$, calculated with the Stokes-Einstein equation:

$$D = \frac{k_B T}{6\pi\eta r_{prey}} \qquad (S4)$$

Using a prey radius r_{prey} of 0.62035 μm (which gives a prey volume of 1 μm^3, a temperature T of 298 K, and a viscosity η of 8.891×10^{-4} Pa·s.

The number of axopods is varied and the positions of the axopods are assigned automatically using the Fibonacci method giving the coordinates for the base of axopod i as:

$$x_i = \mathrm{r}_{pred}\cos\left(\cos^{-1}\left(2\frac{i}{N} - 1\right) - \frac{\pi}{2}\right)\cos\left(2\pi\frac{i}{\phi}\right) \qquad (S5)$$

$$y_i = \mathrm{r}_{pred}\cos\left(\cos^{-1}\left(2\frac{i}{N} - 1\right) - \frac{\pi}{2}\right)\sin\left(2\pi\frac{i}{\phi}\right) \qquad (S6)$$

$$z_i = r_{pred} \sin\left(\cos^{-1}\left(2\frac{i}{N} - 1\right) - \frac{\pi}{2}\right) \qquad (S7)$$

Here, r_{pred} is the predator cell radius (excluding axopods). To obtain the coordinates for the tips of the axopods, the axopod length is added to the predator cell radius. N is the total number of axopods, i is the axopod index and ϕ is the golden ratio, $\frac{1+\sqrt{5}}{2}$.

The length of the axopods is 50 µm and the width 0.28 µm. In the simulation the width of the axopods is extended to include the radius of the prey cells and is 0.28 + 2 × 0.62 = 1.52 µm. The reason for this is that the prey cell particles in the Smoldyn simulation don't have a size, only a position. This position is treated as the center point of the prey cell by including the radius of the prey cells in the simulated axopod width. Axopod dimensions are determined from a survey of the literature and are listed in Table S1 and plotted in Figure S1A and S1B.

Table S1: Cell volumes and axopod dimensions.

Species	Cell volume (µm³)	Axopod width (µm)[a]	Axopod length (µm)	References
**Microheliella maris**	34	0.092	8	[3]
**Ciliophrys marina**	1767	0.10	50	[4]
**Raphidiophrys contractilis**	3618	0.29	113	[5]
**Clathrulina elegans**	9099	0.19	43	[6]
**Actinophrys sol**	41441	0.49	160	[7]
**Actinophrys salsuginosa**	118847	1.64	175	[8]
**Actinophrys tauryanini**	268083	4.20	292	[8]
**Gymnosphaera albida**	543480	1.25	127	[9]
**Actinosphaerium eichhornii**	8181231	0.73	148	[8]

[a] Axopod width is obtained from electron micrographs where it isn't always clear where along the length of the axopod the image was recorded. As axopods can taper their width accidentally extracting the axopod width from a cross-section closer to the tip could reduce the axopod width estimates.

As soon as a prey cell hits an axopod it is removed from the simulation and counted. Prey cells that diffuse back and hit the bounding sphere are permanently removed from the simulation. This together with constant production of new prey cells at the bounding sphere simulates diffusion through the boundary sphere. The rate at which prey cells are hitting the axopods is the capture rate. Five simulations are performed with different numbers of axopods: 5, 10, 20, 40, and 80. Two additional simulations are performed that don't include axopods. One is a spherical cell in which the surface of the cell is capturing the prey (i.e. a collision of a prey cell with the predator surface counts as a capture). The other is essentially the same but with the surface of the sphere where the tips of the axopods would have been (radius = $r_{pred}+L_{axo}$, with L_{axo} being the axopod length). The simulation is allowed to run for 10^5 s. The capture rate is obtained over the second half of this period when a stable prey concentration gradient has been established.

Capture rate as a function of axopod number

To incorporate the capture rate results into the phagocytosis model, the number of axopods is plotted against the capture rate and is fitted with:

$$v_{cap} = \frac{Ac_{prey}N_{axo}}{B + N_{axo}} \qquad (S8)$$

Here, A is a fit parameter that together with the prey concentration (at infinite distance), c_{prey}, gives the maximum capture rate and B is the equivalent of the Michaelis constant in the Michaelis-Menten equation. Equation S8 is an empirical fit and has no theoretical justification at present aside from its similarity to the Michaelis-Menten formalism. To obtain the value of A from the fit, the prey concentration at infinite distance, c_{prey}, is required from the simulation. The simulation itself doesn't include prey concentration at infinite distance *per se* but this can be calculated from the flux through the surface of a sphere that is concentric with the cell. This is obtained from the simulation without axopods and with a sphere radius of $r_{pred}+L_{axo}$, (with L_{axo} being the axopod length in the accompanying simulation that does feature axopods) using the flux through a bounding sphere[10] at distance r:

$$J(r) = -\frac{Dc_{prey}a}{r^2} \qquad (S9)$$

D is the diffusion coefficient of the prey cell, a is the radius of the absorbing boundary, and r is the radius of the sphere at which the flux is examined. Setting r=a and rewriting yields:

$$c_{prey} = \frac{J(a)a}{D} \qquad (S10)$$

Here, the minus sign has been omitted because it is only relevant for the direction of the flux. $J(a)$ is the flux (per unit surface area) over the surface of the absorbing sphere (i.e. the number of prey cells hitting the surface per square micron per second). This is obtained from the simulation (taking the absolute value). Once the c_{prey} is obtained from the simulations the parameter A can be determined from equation S8. Once A is found it can be combined with B and any desired value of c_{prey} and N_{axo} to determine the capture rate. In practice, the phagocytosis model varies the capture rate, v_{cap}, and the number of axopods, N_{axo}, is determined from:

$$N_{axo} = \frac{B}{\frac{Ac_{prey}}{v_{cap}} - 1} \qquad (S11)$$

This can be multiplied with the cost, C_{axo}, or surface area occupancy, A_{axo}, of a single axopod to determine the total cost and area occupancy of the prey capture mechanism.

Energy cost of prey capture

The cost of a single axopod includes the membrane surrounding the axopod, the microtubules in the axopod, and the microtubules extending from the middle of the cell to the base of the axopod:

$$C_{axo} = C_{mem} + C_{MT} \qquad (S12)$$

Here, C_{mem} is the cost of the membrane and C_{MT} the cost of the microtubules. The membrane cost can be determined from the per unit area cost of a membrane, C_{area}, which includes lipids and proteins[11] and by assuming a cylindrical axopod shape:

$$C_{mem} = L_{axo} 2\pi r_{axo} C_{area} \qquad (S13)$$

Here, L_{axo} is the length of the axopod and r_{axo} is the radius of the axopod. The membrane area at the axopod tip is assumed to be equivalent to the membrane area that is removed from the plasma membrane at the base so that only the sides of a cylinder contribute to the axopod membrane cost.

The cost of the microtubules is assumed to be equivalent to the sum of the construction cost of all amino acids (and their assembly into proteins) that are present in the microtubule bundle that supports the axopod and is calculated from the axopod cross-sectional area and the microtubule density (the number of microtubules per unit cross-sectional area). The microtubule density is extracted from electron micrographs (Table S2). The microtubule cost includes the segments that extend from the cell surface to the cell center and is given by:

$$C_{MT} = \frac{N_{MT} L_{MT} N_{pf} N_{AA,tub} C_{AA}}{d_{monomer}} \qquad (S14)$$

Here, N_{MT} is the number of microtubules in an axopod (calculated from the microtubule density and axopod cross-sectional area), L_{MT} is the length of the microtubules (equal to $L_{axo} + r_{predator}$), $N_{pf} = 13$ is the number of protofilaments in a microtubule[11], $N_{AA,tub} = 447$ is the average number of amino acids in a tubulin monomer (averaged over α- and β-tubulin)[11], C_{AA} is the energy cost per average amino acid, and $d_{monomer} = 0.004\ \mu m$ is the distance a single monomer spans along the length of the microtubule[11]. C_{mem} and C_{MT} can be combined to calculate the cost of an individual axopod, C_{axo}. Multiplying the cost of an individual axopod by the number of axopods gives the total cost of prey capture (in units of ATP):

$$C_{cap} = C_{axo} N_{axo} \qquad (S15)$$

Area occupancy of prey capture

The area occupancy of the axopods on the surface of the predator cell, $A_{axo,all}$, is calculated by combining the cross-sectional area of an individual axopod, A_{axo}, with the axopod number, N_{axo}:

$$A_{axo,all} = N_{axo} A_{axo} = \frac{B}{\frac{Ac_{prey}}{v_{cap}} - 1} \pi {r_{axo}}^2 \qquad (S16)$$

With r_{axo} being the axopod radius and N_{axo} being obtained from Eq. S11. Giving the fractional occupancy of prey capture as:

$$f_{occ,cap} = \frac{A_{axo,all}}{A_{pm}} \qquad (S17)$$

A_{pm} is the area of the predator plasma membrane at the start of the cell division period.

Table S2: Microtubule (MT) density in axopod cross-sections.

Species	MT density (μm^{-2})	References
**Actinosphaerium nucleofilum**	655	[12]
**Raphidiophrys elegans**	796	[13]
**Dimorpha mutans**	1006	[13]
**Average**	**819**	

Methods S3: External digestion (ancestor), related to Figure 1 and 2.

In the model, both the ancestor and the phagocytoser have the same heliozoan prey capture mechanism. The difference is in how the captured prey is processed. In the ancestor, the prey is held at the cell surface and enzymes are excreted all over the surface of the predator cell. Some of these enzymes make it to the prey, with probability P_{enz}, and the remainder are lost to the environment. The enzymes that make it to the prey stay on the prey until the prey is completely digested. The nutrients produced by the enzymes diffuse to the predator cell with probability P_{nut}, with the remainder being lost to the environment.

Calculation of P_{enz} and P_{nut}

P_{enz} and P_{nut} are determined using Smoldyn simulations[2]. For P_{enz} there is a large sphere that represents the predator cell and touching it is a small sphere representing the prey cell (Figure S1C). The axopods are not included in these simulations. A thousand particles representing the enzymes are positioned at random over the entire surface of the predator cell. These are released and diffuse with a diffusion coefficient of 85 μm^2/s.[14] As an enzyme particle hits the surface of the prey cell it is removed from the simulation. The number of remaining particles is tracked in time and reaches a plateau (Figure S1C). P_{enz} is calculated by comparing the plateau to the starting enzyme number: $P_{enz} = \frac{N_{start} - N_{plateau}}{N_{start}}$. To determine P_{nut}, an equivalent simulation is performed but now the particles start on the prey sphere and diffuse with a diffusion coefficient of 750 μm^2/s (Figure S1D). Simulations were performed three times for both P_{enz} and P_{nut} with the average value being used in the phagocytosis model.

When many prey are caught in a short time window and/or prey processing takes a long time, there are multiple prey present at the predator surface at any given moment, N_{prey} (Figure S1E). This raises P_{enz} because there is a larger target for the enzymes. The relation between P_{enz} and the number of prey is determined by repeating the simulations as described but with the number of prey cells varying between 1 and 128 (Figure S1E), positioning prey equidistantly on the surface of the predator cell using the Fibonacci method already described for the Heliozoan prey capture mechanism. This simulation data was fitted with:

$$P_{enz} = \frac{A_{penz} N_{prey}}{B_{penz} + N_{prey}} \qquad (S18)$$

Here, $A_{penz} = 1.112$ and $B_{penz} = 13.71$ are fit parameters. This equation is used in the phagocytosis model with the additional rule that if the P_{enz} given by this equation is higher than 1, a value of exactly 1 is used. This equation has no theoretical justification other than fitting the data well and allowing us to adjust the number of prey present on the predator cell surface in the cellular performance calculations without having to redo the simulations.

Calculating the digestion time, t_{dig}

Enzymes accumulate on the prey, and turn the prey biomass, which is considered to consist of pure protein, into amino acids. This digestion takes a time t_{dig} to complete. The digestion time depends on the number of amino acids to be released from the prey, $N_{AA,prey}$, and the number of enzymes accumulating over time. The $N_{AA,prey}$ is given by the integral:

$$N_{AA,prey} = \int_0^{t_{dig}} N_{enz}(t) P_{enz} k_{prot} dt = \frac{N_{Sec} k_{prot} P_{enz}}{t_{ins}} \int_0^{t_{dig}} t dt \qquad (S19)$$

Here, $N_{enz}(t)$ is the number of enzymes excreted by the predator cell for this particular prey after time t. It is the product of the number of Sec translocases, N_{Sec}, available for each prey cell on the predator cell surface and the time it takes to transport an enzyme across the cell surface, t_{ins}. The rate amino acid production, from prey biomass, by a single enzyme is given by k_{prot}. Solving the integral yields:

$$N_{AA,prey} = \frac{N_{Sec} k_{prot} P_{enz} {t_{dig}}^2}{2t_{ins}} \qquad (S20)$$

Rewriting gives the digestion time:

$$t_{dig} = \sqrt{\frac{2t_{ins} N_{AA,prey}}{N_{Sec} k_{prot} P_{enz}}} \qquad (S21)$$

In the phagocytosis model, t_{dig} is a variable and this relation is used to find the number of Sec translocases N_{Sec}.

Calculating the energy cost of external digestion

The enzymes used to degrade prey cells come with an energy cost. The total energy expenditure on enzymes depends on how many enzymes are excreted by the predator. Enzymes are excreted by the predator throughout the digestion time and digestion events happen throughout the cell division period. Each individual enzyme comes with a cost of $N_{AA,enz} C_{AA}$, i.e. the number of amino acids in an enzyme, $N_{AA,enz}$, multiplied by the energetic cost of an (average) amino acid, C_{AA}. The number of enzymes on the prey depends linearly on time (due to a fixed excretion capacity) and is adjusted by the fraction of enzymes that actually make it to the prey, P_{enz}. The excretion of enzymes, or a particular prey cell, stops when the prey has been digested and therefore the total number of enzymes also depends on the number of amino acids present in the biomass of the prey, $N_{AA,prey}$, and the time it requires to digest the prey cell, t_{dig}. Combining these considerations yields the

following integral that can be used to determine the total number of enzymes produced as a function of the digestion time:

$$N_{AA,prey} = \int_0^{t_{dig}} N_{enz}(t) P_{enz} k_{prot} dt = k_{enz} P_{enz} k_{prot} \int_0^{t_{dig}} t dt \qquad (S22)$$

With k_{enz} being the rate of enzyme excretion (for each individual prey cell) and $N_{enz}(t) = k_{enz}t$. Solving the integral and rewriting yields:

$$k_{enz} = \frac{2N_{AA,prey}}{P_{enz} k_{prot} {t_{dig}}^2} \qquad (S23)$$

The number of enzymes produced over the whole digestion period, $N_{enz,prey}$, is obtained by multiplying k_{enz} by t_{dig}:

$$N_{enz,prey} = \frac{2N_{AA,prey}}{P_{enz} k_{prot} t_{dig}} \qquad (S24)$$

The cost of digesting a single prey cell is equal to the product of the number of enzymes required to digest a single prey cell, $N_{enz,prey}$, and the cost per enzyme, $N_{AA,enz}C_{AA}$. The total number of prey capture events can be obtained from the prey capture rate and the cell division time, $v_{capadj}t_{d,anc}$, where v_{capadj} is the prey capture rate adjusted for predator growth (see "Correcting v_{cap} for growth" section) and $t_{d,anc}$ is the cell division time of the ancestor. The absolute energetic cost of external digestion, expressed in units of ATP, is given by:

$$C_{dig,ext} = \frac{2N_{AA,prey}}{P_{enz} k_{prot} t_{dig}} N_{AA,enz} C_{AA} v_{capadj} t_{d,anc} \qquad (S25)$$

Calculating the area occupancy of Sec translocases and nutrient transporters

The amino acids freed from prey biomass by the enzymes are imported by nutrient transporters. Energetic costs of both Sec translocases and nutrient transporters are not accounted for explicitly because they are embedded in the membrane, displacing what was already there and therefore not contributing additional energetic costs. Although Sec translocases and nutrient transporters don't contribute to the energetic cost of external digestion, they do impact the cellular performance of the ancestor by occupying the predator plasma membrane.

The total area occupied by Sec translocases and nutrient transporters depends on their copy numbers in the membrane. The required number of Sec translocases in the predator cell membrane (per prey) is the product of the enzyme production rate, k_{enz} (Eq. S23), and the time it takes a Sec translocon to excrete an enzyme, t_{ins}. The number of Sec translocases for a single prey cell is thus given by:

$$N_{Sec} = k_{enz} t_{ins} = \frac{2N_{AA,prey} t_{ins}}{P_{enz} k_{prot} {t_{dig}}^2} \qquad (S26)$$

The number of nutrient transporters depends on how many nutrients are produced per unit time by the enzymes. The number of enzymes is a function of the number of Sec translocases. The required number of nutrient transporters will vary over time, as enzymes accumulate on the prey, but in the model the nutrient transporter number is constant at the maximum requirement (achieved at t_{dig}). The number of amino acids being produced by the enzymes at time t_{dig} is $\frac{N_{Sec}t_{dig}k_{prot}}{t_{ins}}$. This follows from the fact that the number of enzymes present on the prey is a product of the number of Sec translocases, N_{Sec}, that export the enzymes and the number of enzymes each of these translocases exports over the time t_{dig}, $\frac{t_{dig}}{t_{ins}}$. Each of these enzymes produces nutrients at a rate k_{prot}. Not all amino acids make it back to the cell, giving $\frac{N_{Sec}t_{dig}k_{prot}P_{nut}}{t_{ins}}$ for the number of amino acids making it to the predator. It is assumed that these nutrients are transported by the nutrient transporters at perfect efficiency which gives a transport rate of $N_{trans}k_{trans}$ per prey cell. Where N_{trans} is the number of nutrient transporters per prey cell and k_{trans} is the transport rate per nutrient transporter. Nutrient production (adjusted for nutrient loss, using P_{nut}) must be equal to the nutrient uptake so that:

$$\frac{N_{Sec}t_{dig}k_{prot}P_{nut}}{t_{ins}} = N_{trans}k_{trans} \qquad (S27)$$

This allows us to calculate the number of nutrient transporters, N_{trans}, as a function of the number of Sec translocases, N_{Sec} (obtained from Eq. S26):

$$N_{trans} = \frac{N_{Sec}t_{dig}k_{prot}P_{nut}}{t_{ins}k_{trans}} = \frac{2N_{AA,prey}P_{nut}}{P_{enz}t_{dig}k_{trans}} \qquad (S28)$$

The number of prey present on the predator surface at any given moment is given by $t_{dig}v_{cap}$. Here, v_{cap} is used instead of v_{capadj} because the area occupied by Sec translocases and nutrient transporters is compared to the predator plasma membrane area at the start of the cell cycle. The area occupied by a single Sec translocase or nutrient transporter is A_{mp}. The lipids surrounding the Sec translocases and Nutrient transporters are included in the required area by using the fraction of membrane area that is membrane protein, $f_{memprot}$. Following these considerations, the fractional occupancy of the ancestor plasma membrane by Sec translocases and nutrient transporters, $f_{occ,ext}$, can be calculated as:

$$f_{occ,ext} = \frac{1}{A_{pm}}\frac{A_{mp}}{f_{memprot}}(N_{trans} + N_{Sec})t_{dig}v_{cap} \qquad (S29)$$

A_{pm} is the area of the predator plasma-membrane at the start of the cell division period. Finally, the fractional occupancy of ancestor plasma-membrane, $f_{occ,anc}$, is obtained by including prey capture:

$$f_{occ,anc} = f_{occ,ext} + f_{occ,cap} \qquad (S30)$$

For the fractional occupancy of the prey capture mechanism, $f_{occ,cap}$, see Eq S17.

Methods S4: Engulfment (phagocytoser), related to Figure 1 and 2.

The phagocytoser differs from the ancestor in how the captured prey is processed. After the prey is captured, it is positioned on the surface of the predator plasma membrane where it is engulfed by a phagocytic cup. From the phagocytic cup a food vacuole forms in which the prey is digested (see section on internal digestion). The phagocytic cup comes with an energy cost and an area occupancy.

Energy cost of engulfment

The phagocytic cup starts a circular protrusion of the predator cell membrane which forms around the prey cell. This grows until it completely envelops the prey cell. The phagocytic cup comes together at the apex of the prey where it fuses to produce a new patch of plasma membrane from its external facing membrane and a food vacuole membrane from the prey-facing membrane. The phagocytic cup is composed of membrane, actin, and actin accessory proteins.

The membrane cost is determined by multiplying the total membrane area by the per unit area energy cost of a membrane, 1.45×10^{9} ATP μm^{-2} (including both lipids and membrane proteins)[11].

Actin abundances per unit volume are obtained from structures that are similar to phagocytic cups, i.e. actin-based structures that manipulate membranes. Estimates of actin abundance and structure dimensions were obtained for a filopodium[15], lamellipodium[16], and for endocytic vesicle construction[17]. For the lamellipodium calculation monomer number per unit actin filament length was obtained from[18,19]. From the actin abundance, densities of actin monomers were calculated of 1.33×10^{5} μm^{-3} for the filopodium, 5.85×10^{5} μm^{-3} for the lamellipodium, and 1.22×10^{6} μm^{-3} for the endocytic vesicle. The cost of actin per cubic micron is given by:

$$C_{actin} = d_{actin} N_{AA,actin} C_{AA} \qquad (S31)$$

Here, d_{actin} is the density of actin monomers, $N_{AA,actin}$ is the number of amino acids per actin monomer (376 amino acids), and C_{AA} is the energy cost per average amino acid. This leads to a geometric mean actin energy cost of 4.97×10^{9} ATP μm^{-3}.

The abundance of actin accessory proteins is estimated from three different sources. The first is the abundance[20] of actin associated proteins compared to actin[21] in whole cells of *Dictyostelium discoideum*. If the elongation factors are excluded from the list of actin associated proteins the abundance of actin + actin associated proteins is 4-fold that of actin alone. The second and third estimates of actin accessory protein cost, which accounts for differences in protein amino acid length, come from endocytic vesicle construction in *Schizosaccharomyces pombe*[22]. The second estimate uses the total cytoplasmic abundance of actin compared to that of other proteins involved in endocytic vesicle construction and results in an energy cost multiplier of 3.4-fold. The third estimate derives from the abundance of actin and accessory proteins at the site of endocytic vesicle construction and results in an energy cost multiplier of 2.4. This gives an average multiplier of 3.3-fold and an energetic cost of actin plus accessory proteins of 1.6×10^{10} ATP μm^{-3}.

The membrane and phagocytic cup plasm costs (actin plus accessory proteins) can be combined with information about the size and shape of phagocytic cups to obtain an estimate of the energy cost of engulfment. The calculation is performed for two phagocytic cups of differing size and shape, one from the choanoflagellate *Codosiga*[23] and the other from a mouse macrophage[24]. For the choanoflagellate this yields a phagocytic cup cost of 3.7x10^{10} ATP for an enclosed volume (or prey volume) of 0.36 μm^3, giving an energy cost per unit enclosed volume of 10.0x10^{10} ATP μm^{-3}. For the mouse macrophage these numbers are 1.1x10^{12} ATP, 38 μm^3, and 2.8x10^{10} ATP μm^{-3}.

These estimates are combined with the energy cost of producing an endocytic vesicle[1]. For the endocytic vesicle we have a vesicle construction cost of 1.7x10^7 ATP, an enclosed volume of 3.9x10^{-5}, and an energy cost per unit enclosed volume of 4.3x10^{11} ATP μm^{-3}. A regression is performed on these three datapoints yielding the energy cost of engulfing a volume as:

$$C_{eng,single} = 6.65 \times 10^{10} {V_{eng}}^{0.808} \qquad (S32)$$

The engulfment cost, $C_{eng,single}$, has units of ATP and the engulfment volume, V_{eng}, has units of μm^3. To obtain the total cost of engulfment the cost of a single engulfment is multiplied by the number of simultaneous engulfment events, $t_{eng} v_{capadj}$:

$$C_{eng} = C_{eng,single} t_{eng} v_{capadj} \qquad (S33)$$

With t_{eng} being the time required to engulf the prey and v_{capadj} being the growth adjusted prey capture rate (see Eq. S40 and S41).

Area of engulfment

The area occupancy of the phagocytic cup is obtained by assuming a spherical prey with a volume of 1 μm^3 and a phagocytic cup thickness of 0.11 μm obtained from the choanoflagellate[23]. This yields an area occupancy, A_{phago}, of 1.67 μm^2, which is divided by two to account for growth and decline. It is assumed that the outer surface of the phagocytic cup can't be used by any other cellular process.

The fractional area occupancy for the phagocytoser includes prey capture, the sum of the cross-sectional areas of all the axopods, and engulfment, the sum of the average area occupancy of all the phagocytic cups being present at the same time, and is given by:

$$f_{occ,phago} = \frac{\frac{1}{2} A_{phago} t_{eng} v_{capadj} + A_{axo,all}}{A_{pm}} \qquad (S34)$$

A_{phago} is the area occupancy of a single phagocytic cup at maximum extent, t_{eng} is the time of engulfment, v_{capadj} is the growth adjusted prey capture rate, $A_{axo,all}$ is the area occupancy of prey capture, and A_{pm} is the plasma membrane area of the predator. It is assumed that the area occupancy of a single phagocytic cup increases linearly with time from a starting point of 0, so that the average phagocytic cup area occupancy is $\frac{1}{2} A_{phago}$.

Difference in energetic cost per engulfment volume

The choanoflagellate phagocytic cup is 3.6-fold as expensive, per engulfment volume, as the mouse macrophage. This is due to a 1.8-fold increase in phagocytic cup plasm cost and a 12.5-fold increase in membrane cost. Given the engulfment volumes involved, the change in membrane cost expected from a difference in surface area to volume alone, assuming spherical prey, is 6.8-fold, with the remaining 1.8-fold being due to differences in phagocytic cup shape. Thus, the choanoflagellate cup is more expensive mostly because of its small size.

Methods S5: Internal digestion in a food vacuole (phagocytoser), related to Figure 1 and 2

Upon completion of prey engulfment, a food vacuole is formed in the predator cytoplasm. The food vacuole membrane contains Sec translocases that specifically transport enzymes to convert the prey biomass into amino acids (as with external digestion, it is assumed that prey is composed entirely of protein). These amino acids are transported from the food vacuole lumen and into the cytoplasm by nutrient transporters. The key parameter controlling the internal digestion process is the digestion time, t_{dig}. This sets the requirement for Sec translocases and nutrient transporters, it determines the efficiency of digestion (by setting the time that individual enzymes spend on the prey), and it determines the contribution of the food vacuole membrane energy cost to the predator whole cell cost.

Digestion time, t_{dig}

The digestion time is calculated similar to that of the ancestor (Eq. S21), except that all enzymes make it to the prey (i.e. $P_{enz} = 1$), yielding:

$$t_{dig} = \sqrt{\frac{2t_{ins}N_{AA,prey}}{N_{Sec}k_{prot}}} \qquad (S35)$$

As with the ancestor, t_{dig} is treated as a variable in the phagocytosis model, but this equation is used to obtain the number of Sec translocases, N_{Sec}.

Cost of food vacuole membrane

The energy cost of the food vacuole membrane is a product of the food vacuole membrane area, a constant related to the prey volume, V_{prey}, and the energy cost per unit membrane area, C_{area}. For simplicity, the food vacuole is assumed to be equal in size to the prey. The area of such a food vacuole is then given by $4\pi\left(\frac{3V_{prey}}{4\pi}\right)^{\frac{2}{3}}$. Since food vacuole membranes are recycled, we only need to account for the costs of the food vacuoles that are present simultaneously. To account for the number of food vacuoles being present at any given moment, the cost per food vacuole is multiplied by the number of food vacuoles. This number is the product of the digestion time and the adjusted prey capture rate, $t_{dig}v_{capadj}$. Thus, we obtain:

$$C_{fv} = 4\pi\left(\frac{3V_{prey}}{4\pi}\right)^{\frac{2}{3}} C_{area}t_{dig}v_{capadj} \qquad (S36)$$

Cost of enzymes

The energy cost of the enzymes that digest the prey inside of the food vacuoles needs to be included in the total cost of internal digestion. This differs from external digestion in that no enzymes are lost before they are used, but it is similar in that the enzymes are ultimately lost. Enzymes are lost because in the primitive phagocytosis examined here, there is no mechanism for recycling the enzymes. They are lost to the external medium upon food vacuole fusion with the plasma membrane after the prey has been digested. The total energy cost of the enzymes used for internal digestion can be calculated similarly to that for external digestion (Eq. S25) except that $P_{enz} = 1$, yielding:

$$C_{enz,int} = \frac{2N_{AA,prey}}{k_{prot}t_{dig}} N_{AA,enz}C_{AA}v_{capadj}t_{d,phago} \qquad (S37)$$

Like for external digestion, the total enzyme cost depends on the cell division time, $t_{d,phago}$.

Methods S6: Calculating relative cost and corrections for cell growth, related to Figure 2

Predator energy budget

The energetic costs calculated so far are absolute costs of constructing the various cellular traits, expressed in units of ATP. Only by normalizing these costs to the cellular budget can these absolute costs be made relevant for the calculation of cellular performance. Here, we use the cell budget before any of the cellular traits discussed here (prey capture, engulfment, etc.) are added, C_{budget}, to calculate a relative energetic cost. C_{budget} is obtained from the predator volume, V_{pred}, by a simplified empirical relation[25]:

$$C_{budget} = 2.7 \times 10^{10} {V_{pred}}^{0.97} \qquad (S38)$$

As the cell cycle progresses the predator cell grows as does the cell budget. To account for this an average cell budget can be calculated. The average value depends on the pattern of growth, exponential, linear, etc. Here, the pattern of growth is set by the prey capture mechanism. It can be assumed that the number of axopods doubles over the cell division period, but the rate at which axopods are added depends on how the capture rate changes with the axopod number. At optimal axopod numbers, the doubling of axopod number during cell division changes the prey capture rate only marginally. For this reason, and for the sake of simplicity, the growth is assumed to be linear. This means that the average cellular energy budget, given by $C_{budget}f_{budget}$, can be calculated by setting $f_{budget} = 1.5$.

Correcting v_{cap} for growth

As the predator grows, its capacity to capture prey, and accumulate biomass, increases with the rising number of axopods. This effect can be incorporated into the phagocytosis model by assuming a linear rate of increase in the number of axopods, N_{axo}, and then taking the average over the prey capture rate (see Eq. S11):

$$v_{capadj} = \frac{1}{N_{axo}{}^{*}} \int_{N_{axo}{}^{*}}^{2N_{axo}{}^{*}} \frac{Ac_{prey}N_{axo}}{B + N_{axo}} dN_{axo} \qquad (S39)$$

The fraction $\frac{1}{N_{axo}{}^{*}}$ normalizes the integral so that we obtain the average capture rate over the cell cycle period, as the integral is taken over an interval with length $N_{axo}{}^{*}$. $N_{axo}{}^{*}$ is the number of axopods at the start of the cell cycle and is given by:

$$N_{axo}{}^{*} = \frac{v_{cap}B}{Ac_{prey} - v_{cap}} \qquad (S40)$$

Here, c_{prey} is the prey concentration far from the predator cell. Solving the integral yields:

$$v_{capadj} = \frac{Ac_{prey}}{N_{axo}{}^{*}} \left(N_{axo}{}^{*} + Bln(N_{axo}{}^{*} + B) - Bln(2N_{axo}{}^{*} + B)\right) \qquad (S41)$$

This is used to calculate v_{capadj} from v_{cap}. Note that we distinguish between $N_{axo}{}^{*}$, the number of axopods at the start of the cell cycle, and N_{axo}, the number of axopods at some time during the cell cycle. This distinction has no relevance outside of this section and therefore Eq. S11 and S40 are identical.

Delayed nutrient return penalty

For both the ancestor and the phagocytoser the digestion time is varied. The longer the digestion time, t_{dig}, the more efficient the production of amino acids by enzymes, i.e. fewer enzymes convert the same amount of biomass into amino acids but over a longer period. However, if an amino acid is produced and returned to the cell late, this reduces its value because the cell has increased in volume in the meantime. Thus, a larger t_{dig} also reduces the value of an amino acid. This effect is accounted for by a delayed return penalty. The degree to which the nutrient value is reduced depends on the time between the production of the enzyme and the delivery of the nutrient, t_{α}. The value of t_{α} can range between 0 and t_{dig}. And for each specific enzyme it varies between 0 and $t_{dig} - t_{\beta}$, with t_{β} being the time between the start of the digestion period for a specific prey cell and the production of the enzyme. Assuming a linear increase in volume over the cell division time (approximately true when t_{α} is small compared to t_d), the nutrient value is given by:

$$Nutrient\ value = 1 - \frac{1}{2}\frac{t_{\alpha}}{t_d} \qquad (S42)$$

The delayed return penalty, or average nutrient value, N_{value}, is calculated by integrating over all enzymes (produced over a time interval equal to t_{dig}) and all nutrients released from each enzyme (produced over a time interval equal to $t_{dig} - t_{\beta}$):

$$N_{value} = \frac{\int_0^{t_{dig}} \int_0^{t_{dig}-t_\beta} \left(1 - \frac{1}{2}\frac{t_\alpha}{t_d}\right) dt_\alpha dt_\beta}{\int_0^{t_{dig}} \int_0^{t_{dig}-t_\beta} 1\, dt_\alpha dt_\beta} \qquad (S43)$$

Here, $\int_0^{t_{dig}} \int_0^{t_{dig}-t_\beta} 1\, dt_\alpha dt_\beta$ is a normalization constant. Solving the integrals yields:

$$N_{value} = 1 - \frac{t_{dig}}{6t_d} \qquad (S44)$$

This value is different for the ancestor and phagocytoser, giving us $N_{value,anc}$ and $N_{value,phago}$.

Methods S7: Calculation of $t_{d,anc}$ and $t_{d,phago}$, related to Figure 2

The cell division time of the ancestor can be calculated by adding all the costs together to get a nutrient requirement and modifying the nutrient uptake rate by the delayed nutrient return penalty. This yields an ancestral division time of:

$$t_{d,anc} = \frac{N_{nutV} V_{pred} \left(1 + C_{trait,anc}\right)}{N_{AA,prey} P_{nut} v_{capadj} N_{value,anc}} \qquad (S45)$$

N_{nutV} is the nutrient (or amino acid) requirement per unit predator volume, V_{pred} is the predator volume, $C_{trait,anc}$ is the cost of prey capture and external digestion, and $N_{AA,prey} P_{nut} v_{capadj}$ is the number of nutrient molecules arriving per unit time. Substituting the trait costs (Eq. S15 for prey capture and S25 for external digestion) and $N_{value,anc}$ (N_{value} in Eq. S44) yields:

$$t_{d,anc} = \frac{\delta_1 + \delta_2 + \varepsilon_1 t_{d,anc}}{\delta_3 \left(1 - \frac{t_{dig}}{6t_{d,anc}}\right)} = \frac{\delta_1 + \delta_2 + \varepsilon_1 t_{d,anc}}{\delta_3 - \frac{\delta_3 t_{dig}}{6} \frac{1}{t_{d,anc}}} = \frac{\delta_1 + \delta_2 + \varepsilon_1 t_{d,anc}}{\delta_3 - \frac{\varepsilon_2}{t_{d,anc}}} \qquad (S46)$$

$$\delta_1 = N_{nutV} V_{pred} \qquad (S46a)$$

$$\delta_2 = N_{nutV} V_{pred} C_{cap} \frac{1}{C_{budget}} \qquad (S46b)$$

$$\delta_3 = N_{AA,prey} f_{mod} P_{nut} v_{capadj} \qquad (S46c)$$

$$\varepsilon_1 = N_{nutV} V_{pred} \left(\frac{2N_{AA,prey}}{f_{mod} P_{enz} k_{prot} t_{dig}} N_{AA,enz} C_{AA} v_{capadj} \right) \frac{1}{C_{budget} f_{budget}} \qquad (S46d)$$

$$\varepsilon_2 = N_{AA,prey} f_{mod} P_{nut} v_{capadj} \frac{t_{dig}}{6} \qquad (S46e)$$

The multiplier f_{mod} adjusts the base enzyme to prey, P_{enz}, and nutrient to predator, P_{nut}, probabilities to account for e.g. flow of the surrounding media. The value of f_{mod} is set to 1, except for the analysis presented in Figure 2A. The ancestral cell division time can be obtained from the deltas and epsilons by rewriting the equation:

$$t_{d,anc} = \frac{\delta_1 + \delta_2 + \varepsilon_2}{\delta_3 - \varepsilon_1} \qquad (S47)$$

Note that because of these rearrangements the cost of the enzymes (accounted for by ε_1) are now in the denominator. This is a consequence of the enzyme cost being dependent on the cell division time, as explained in the enzyme cost section above. The cell division time of the phagocytoser is obtained similarly but with $P_{nut} = 1$ and the cost of the trait being due to prey capture (Eq. S15), engulfment (Eq. S33), and internal digestion (Eq. S36 and S37):

$$t_{d,phago} = \frac{N_{nutV} V_{pred} \left(1 + C_{trait,phago}\right)}{N_{AA,prey} v_{capadj} N_{value,phago}} \qquad (S48)$$

Substituting the trait costs and N_{value} yields:

$$t_{d,phago} = \frac{\alpha_1 + \alpha_2 + \alpha_3 + \alpha_4 + \beta_1 t_{d,anc}}{\alpha_5 \left(1 - \frac{t_{dig}}{6 t_{d,phago}}\right)} = \frac{\alpha_1 + \alpha_2 + \alpha_3 + \alpha_4 + \beta_1 t_{d,phago}}{\alpha_5 - \frac{\beta_2}{t_{d,phago}}} \qquad (S49)$$

$$\alpha_1 = N_{nutV} V_{pred} \qquad (S49a)$$

$$\alpha_2 = N_{nutV} V_{pred} C_{eng} \frac{1}{C_{budget} f_{budget}} \qquad (S49b)$$

$$\alpha_3 = N_{nutV} V_{pred} C_{cap} \frac{1}{C_{budget}} \qquad (S49c)$$

$$\alpha_4 = N_{nutV} V_{pred} C_{fv} \frac{1}{C_{budget} f_{budget}} \qquad (S49d)$$

$$\alpha_5 = N_{AA,prey} v_{capadj} \qquad (S49e)$$

$$\beta_1 = N_{nutV} V_{pred} \left(\frac{2 N_{AA,prey}}{k_{prot} t_{dig}} N_{AA,enz} C_{AA} v_{capadj}\right) \frac{1}{C_{budget} f_{budget}} \qquad (S49f)$$

$$\beta_2 = N_{AA,prey} v_{capadj} \frac{t_{dig}}{6} \qquad (S49g)$$

The cell division time of the phagocytoser can be obtained from the alphas and betas by rewriting the equation:

$$t_{d,phago} = \frac{\alpha_1 + \alpha_2 + \alpha_3 + \alpha_4 + \beta_2}{\alpha_5 - \beta_1} \qquad (S50)$$

Like the ancestor the cost of the enzymes (accounted for by β_1) are now in the denominator because of the rearrangement of terms.

Methods S8: Filters for data, related to Figure 2

The cells considered here suffer from several internal constraints that are not directly accounted for by the phagocytosis model itself. To make sure that the presented data don't include transgressions of these constraints, several filters are applied. These concern (1) the area limit imposed by the size of the food vacuole on the number of Sec translocases and nutrient transporters, (2) the limit imposed by the predator cell volume on the number of food vacuoles present simultaneously, and (3) the area limit imposed by the surface of the ancestral predator on the number of prey that are digested externally at the same time. In addition, the data is screened for fractional occupancies in the ancestor, $f_{occ,anc}$, and phagocytoser, $f_{occ,phago}$, that exceed 1.

Food vacuole area limit

The area occupied by Sec translocases and nutrient transporters on the food vacuole membrane is a function of their numbers. The requirement for Sec translocases, N_{Sec}, is set by the number of enzymes to be imported into the food vacuole, $N_{enz}(t) = N_{Sec}\frac{t}{t_{ins}}$ (with t_{ins} being the time required to import a single enzyme). This in turn depends on the number of reactions that need to be catalyzed (equal to the number of amino acids in the prey, $N_{AA,prey}$), the rate of catalysis of the enzymes (k_{prot}), and the digestion time (t_{dig}). From these considerations we obtain the following integral:

$$N_{AA,prey} = \int_0^{t_{dig}} N_{enz}(t)k_{prot}dt = \int_0^{t_{dig}} N_{Sec}\frac{t}{t_{ins}}k_{prot}dt \qquad (S51)$$

Solving the integral yields:

$$N_{AA,prey} = \frac{N_{Sec}k_{prot}{t_{dig}}^2}{2t_{ins}} \qquad (S52)$$

Finally, rewriting gives the number of Sec translocases as:

$$N_{Sec} = \frac{2N_{AA,prey}t_{ins}}{k_{prot}{t_{dig}}^2} \qquad (S53)$$

Next, we obtain the number of nutrient transporters, N_{trans}. The maximum nutrient production rate for enzymes is $N_{enz}\frac{t_{dig}}{t_{ins}}k_{prot}$, this needs to matched by the nutrient transport rate, $N_{trans}k_{trans}$, yielding the number of nutrient transporters as:

$$N_{trans} = \frac{N_{enz}t_{dig}k_{prot}}{t_{ins}k_{trans}} \qquad (S54)$$

The area occupancy of the Sec translocases and nutrient transporters is:

$$A_{SecTrans} = \frac{A_{mp}}{f_{memprot}}(N_{Sec} + N_{trans}) \qquad (S55)$$

With A_{mp} being the area occupancy of single membrane protein and $f_{memprot}$ being the fraction of the membrane being proteins (the remainder being lipids). The fractional occupancy on the food vacuole is:

$$f_{occ,fv} = \frac{A_{SecTrans}}{A_{fv}} = \frac{A_{SecTrans}}{4\pi\left(\frac{3V_{prey}}{4\pi}\right)^{\frac{2}{3}}} \qquad (S56)$$

Here, V_{prey} is the prey volume and A_{fv} is the surface area of the food vacuole. Datapoints for which $f_{occ,fv} > 1$ are removed from the analysis.

Predator cell volume limit

The number of food vacuoles that is present in the phagocytosing predator simultaneously is calculated from the digestion time and the prey capture rate:

$$N_{fv} = t_{dig}v_{cap} \qquad (S57)$$

The volume of the food vacuole is (approximately) equal to that of the prey giving a total food vacuole volume of $N_{fv}V_{prey}$, which leads to a fractional volume occupancy of:

$$f_{occ,Vol} = \frac{t_{dig}v_{cap}V_{prey}}{V_{pred}} \qquad (S58)$$

Datapoints for which $f_{occ,Vol} > 1$ are removed from the analysis.

Surface area limit for external digestion

The number of prey cells being present simultaneously on the cell surface of the ancestral predator is calculated from the digestion time and the prey capture rate:

$$N_{prey} = t_{dig}v_{cap} \qquad (S59)$$

The area occupied by a single prey cell is $\pi {r_{prey}}^2$ (the cross-sectional area in the middle of the cell), yielding a fractional occupancy of prey cells on the predator cell surface of:

$$f_{occ,prey} = \frac{\pi {r_{prey}}^2 t_{dig}v_{cap}}{A_{pm}} \qquad (S60)$$

Datapoints for which $f_{occ,prey} > 1$ are removed from the analysis.

Methods S9: Comparing cellular performance of the phagocytoser to that of thre ancestor, related to Figure 2

The cellular performance of the phagocytoser is calculated relative to that of the ancestor. This is done by a two-step method. First, the digestion time, t_{dig}, and the prey capture rate, v_{cap}, are varied and the cell division time and area occupancies are calculated for the ancestor and phagocytoser separately. Taking just the ancestor cell division times and area occupancies, cellular performance

is calculated by comparing all parameter combinations to a single selected parameter combination. The same is performed for the phagocytoser. For both ancestor and phagocytoser the maximal cellular performance is found, and the cell division time and area occupancy associated with that maximal cellular performance are retrieved. In the second step, the cellular performance of the optimal phagocytoser is compared to the optimal ancestor for ranges of nutrient return probability, P_{nut}, and engulfment time, t_{eng}. For each combination of P_{nut} and t_{eng}, step one is repeated and the cellular performance of the phagocytoser is calculated relative to the ancestor.

Methods S10: Phagocytosis intermediates, related to Figure 3

No food vacuole fusion

Phagocytosis in which food vacuoles don't fuse with the plasma membrane can be modeled by modifying the cell division equation for the phagocytoser (Eq. S50). When food vacuoles are no longer being recycled, they are functionally behaving in the same way as the enzymes (which are disposed of after each prey digestion). When the food vacuoles no longer fuse the enzymes and food vacuole membrane remain physically within the cell (and its progeny) but as far as energy costs are concerned they are lost after each prey is digested. The account for this , the effect of the energy cost of the steady state food vacuole number, α_4, must be removed from the cell division time calculation and replaced by an effect similar to that of enzymes (Eq. S49f), yielding:

$$t_{d,phago} = \frac{\alpha_1 + \alpha_2 + \alpha_3 + \beta_2}{\alpha_5 - \beta_1 - \beta_3} \qquad (S61)$$

with

$$\beta_3 = N_{nutV} V_{pred} \left(A_{fv} C_{area} v_{capadj} \right) \frac{1}{C_{budget} f_{budget}} \qquad (S62)$$

A_{fv} is the surface area of a single food vacuole and C_{area} is the energy cost per unit membrane area (composed of lipids and membrane proteins). An amount of energy equal to $A_{fv}C_{area}$ is removed at a rate v_{capadj}. Because the total amount of energy lost depends on the cell division time, β_3 appears in the denominator with the enzyme cost (represented by β_1).

Low area availability on the food vacuole

A food vacuole with low selectivity for Sec translocons and nutrient transporters can be modeled by adjusting the data selection criterion (see "*Food vacuole area limit*" section) for the surface area occupancy of Sec translocases and nutrient transporters, already included in the model, from 1 to 0.1.

Large phagocytic cups

Larger phagocytic cups are modeled by recalculating the energy cost and area occupancy for a phagocytic cup with thicker walls. The cost of the cup is given by a sum of the contributions of the membrane and the cup plasm:

$$C_{cup} = A_{cup}C_{area} + V_{cup}C_{plasm} \qquad (S63)$$

C_{area} is the energy cost per unknit membrane, C_{plasm} is the energy cost per unit cup plasm (see "*Energy cost of engulfment*" section), A_{cup} is the membrane area of the phagocytic cup, and V_{cup} is the volume of the cup plasm. The area of the cup is given by:

$$A_{cup} = A_{prey} + 2\pi(r_{prey} + d_{cup})r_{prey} + 2\pi(r_{prey} + d_{cup})^2 - \pi(r_{prey} + d_{cup})^2 \qquad (S64)$$

A_{prey} is the surface area of the prey, r_{prey} is the radius of the prey, and d_{cup} is the thickness of the phagocytic cup. Here, $2\pi(r_{prey} + d_{cup})r_{prey}$ is the area of a cylinder that represents the bottom part of the cup, $2\pi(r_{prey} + d_{cup})^2$ is the hemisphere that represents the top of the cup. In the presence of a phagocytic cup a part of the plasma membrane, $\pi(r_{prey} + d_{cup})^2$, isn't required so that is removed from the costs. The volume of the phagocytic cup is approximated by a combination of the volume of a cylinder and a hemisphere with the volume of the prey being subtracted from that sum, yielding:

$$V_{cup} = \frac{1}{2}\frac{4\pi}{3}(r_{prey} + d_{cup})^3 + \pi(r_{prey} + d_{cup})^2 r_{prey} - V_{prey} \qquad (S65)$$

V_{prey} is the prey volume. The surface area occupancy of the phagocytic cup is given by:

$$A_{occ,cup} = \pi(r_{prey} + d_{cup})^2 \qquad (S66)$$

To determine the consequences of large phagocytic cups for the cellular performance, d_{cup} is set to 1 μm and the area occupancy and energy cost so calculated are substituted into the cellular performance model.

Methods S11: Smaller predator cell volumes, related to Figure 4

The cell volume of the prokaryote that gave rise to the first phagocytic organism may not have started at 1000 μm^3, the typical eukaryotic cell volume examined in the bulk of this work. To account for smaller cell volumes (10 and 100 μm^3), the model was modified to exclude an explicit treatment of the heliozoan prey capture mechanism (because this mechanism may not have been around when phagocytosis evolved) and replace this with a simple prey capture rate. We also remove prey capture cost and surface area occupancy from the calculations. For the cell division time of the ancestor, we obtain (see Eq. S46 and S47):

$$t_{d,anc} = \frac{\delta_1 + \varepsilon_2}{\delta_3 - \varepsilon_1} \qquad (S67)$$

For the phagocytoser, we obtain (see Eq. S49 and S50):

$$t_{d,phago} = \frac{\alpha_1 + \alpha_2 + \alpha_4 + \beta_2}{\alpha_5 - \beta_1} \qquad (S68)$$

For the surface area occupancy of the ancestor, we obtain (see Eq. S29 and S30):

$$f_{occ,anc} = f_{occ,ext} = \frac{1}{A_{pm}} \frac{A_{mp}}{f_{memprot}} (N_{trans} + N_{Sec}) t_{dig} v_{cap} \qquad (S69)$$

For the surface area occupancy of the phagocytoser, we obtain (see Eq. S34):

$$f_{occ,phago} = \frac{\frac{1}{2} A_{phago} t_{eng} v_{capadj}}{A_{pm}} \qquad (S70)$$

The value of the prey capture rate is anchored to that of the heliozoan prey capture model but is adjusted to account for a lower capture rate in the smaller cells by multiplying the v_{cap} with $\frac{V_{pred}}{V_{large}}$. Here, V_{large} is set to 1000 μm^3, which is the volume for which the prey capture rates were determined, and the predator cell volume, V_{pred}, is set to 10, 100, and 1000 μm^3. The base prey capture rates used are 0.002, 0.007, and 0.02 prey s^{-1}, which are within the range calculated for the heliozoan in this work. Results are shown in Figure 4A, 4B, and S3.

Methods S12: Prey escape from the ancestral state, related to Figure S4

Conceivably, engulfment will help keep the prey cells attached to the phagocytic predator where they might have escaped the non-phagocytic ancestor. We consider an escape as a reduction in the prey capture rate, replacing v_{capadj} with $f_{ret} v_{capadj}$ everywhere in Eq. 46 except for the capture costs (δ_2). Here, f_{ret} is the fraction of prey that is retained. The same substitution is made in the area occupancy equation, Eq. 30, but again with the exception of the area occupancy of prey capture, $f_{occ,cap}$. Cellular performance is calculated with this modified ancestral cell division time and fractional occupancy. When the f_{ret} is set to 0.9, the gain in cellular performance of the phagocytoser is approximately 10% (Figure S4A and S4B).

Methods S13: Increased efficiency of digestion in the phagocytoser, related to Figure S4

The phagocytoser has the ability to control the pH of the food vacuole and thereby it could improve the efficiency with which it digests the prey cell. This effect is introduced into the model by reducing the efficiency in the ancestor relative to the phagocytoser. This is accomplished by modifying Eq. 46 such that:

$$\delta_3 = N_{AA,prey} f_{eff} f_{mod} P_{nut} v_{capadj} \qquad (S71)$$

and

$$\varepsilon_2 = N_{AA,prey} f_{eff} f_{mod} P_{nut} v_{capadj} \frac{t_{dig}}{6} \qquad (S46e)$$

Here, f_{eff} is the fractional efficiency which is set to 0.8. This means that only 80% of nutrients in the prey are available to the ancestor compared to 100% in the phagocytoser. The modifier, f_{mod}, is set to 1. Cellular performance calculated with this modified cell division time equation reveals an approximately 20% increase in cellular performance for the phagocytoser (Figure S4C and S4D).

Methods S14: Protease turnover numbers, related to Figure 2

Protease turnover numbers were obtained from the BRENDA database, limiting the data to protein or peptide substrates. Turnover numbers vary by orders of magnitude and are listed in Table S3.

Table S3: Protease turnover numbers

Enzyme	Organism	Substrate	Turnover Number (s^{-1})	Reference
**Cytosol nonspecific dipeptidase**	Mus musculus	Ala-Gly	43.3	[26]
**Cytosol nonspecific dipeptidase**	Platyrrhini	Ala-Leu	1190	[27]
**Cytosol nonspecific dipeptidase**	Platyrrhini	Ala-Leu	6510	[27]
**Cytosol nonspecific dipeptidase**	Platyrrhini	Gly-Val	7450	[27]
**Cytosol nonspecific dipeptidase**	Platyrrhini	Gly-Phe	8190	[27]
**Cytosol nonspecific dipeptidase**	Platyrrhini	Gly-Ile	10700	[27]
**Cytosol nonspecific dipeptidase**	Platyrrhini	Gly-Leu	10900	[27]
**Cytosol nonspecific dipeptidase**	Platyrrhini	Gly-Phe	20400	[27]
**Cytosol nonspecific dipeptidase**	Platyrrhini	Gly-Val	21400	[27]
**Cytosol nonspecific dipeptidase**	Platyrrhini	Gly-Ile	23000	[27]
**Cytosol nonspecific dipeptidase**	Platyrrhini	Gly-Leu	23100	[27]
**Cytosol nonspecific dipeptidase**	Mus musculus	Ala-Ile	33300	[26]
**Endopeptidase Clp**	Escherichia coli	Oxidized insulin B-chain	0.5	[28]
**Endopeptidase Clp**	Escherichia coli	Insulin chain B	0.35	[29]
**Endopeptidase Clp**	Escherichia coli	Glucagon	0.5	[28]
**Endopeptidase Clp**	Escherichia coli	Phe-Ala-Pro-His-Met-Ala-Leu-Val-Pro-Val	13.3	[28]
**Endopeptidase Lon**	Escherichia coli	Casein	0.0333	[30]
**Endopeptidase Lon**	Escherichia coli	Oxidized insulin B-chain	0.333	[30]
**Endopeptidase Lon**	Escherichia coli	Beta-galactosidase fragment 3-93	0.84	[31]

**Endopeptidase Lon**	Escherichia coli	Bacteriophage lambda N-protein	1	[30]
**Leucyl aminopeptidase**	Bos taurus	Cys-Gly	2.6	[32]
**Leucyl aminopeptidase**	Salmonella enterica	Asp-Leu-Lys	13	[33]
**Leucyl aminopeptidase**	Bos taurus	Ser-Gly	17	[32]
**Leucyl aminopeptidase**	Salmonella enterica	Asp-Ile-Gly-Gly	26	[33]
**Leucyl aminopeptidase**	Bos taurus	Ser-Gly	42	[32]
**Leucyl aminopeptidase**	Bos taurus	Cys-Gly	56.66	[32]
**Leucyl aminopeptidase**	Bos taurus	Cys-Gly	57	[32]
**Leucyl aminopeptidase**	Bos taurus	Cys-Gly	67	[32]
**Leucyl aminopeptidase**	Bos taurus	Cys-Gly	100	[32]
**Leucyl aminopeptidase**	Bos taurus	Cys-Gly	118	[32]
**Leucyl aminopeptidase**	Bos taurus	Cys-Gly	118.3	[32]
**Leucyl aminopeptidase**	Bos taurus	Met-Gly	135.8	[32]
**Leucyl aminopeptidase**	Bos taurus	Met-Gly	136	[32]
**Leucyl aminopeptidase**	Bos taurus	Leu-Gly	191.7	[32]
**Leucyl aminopeptidase**	Bos taurus	Leu-Gly	192	[32]
**Leucyl aminopeptidase**	Salmonella enterica	Leu-Tyr	351	[33]
**Leucyl aminopeptidase**	Bos taurus	Leu-Gly	400	[32]
**Leucyl aminopeptidase**	Salmonella enterica	Asp-His	418	[33]
**Leucyl aminopeptidase**	Bos taurus	Met-Gly	468	[32]
**Leucyl aminopeptidase**	Bos taurus	Met-Gly	486.3	[32]
**Leucyl aminopeptidase**	Salmonella enterica	Asp-Phe	500	[33]
**Leucyl aminopeptidase**	Salmonella enterica	Asp-Tyr	500	[33]
**Leucyl aminopeptidase**	Salmonella enterica	Asp-Leu	666	[33]
**Leucyl aminopeptidase**	Bos taurus	Leu-Gly	675	[32]
**Leucyl aminopeptidase**	Salmonella enterica	Asp-Gln	761	[33]
**Leucyl aminopeptidase**	Salmonella enterica	Glu-Leu	866	[33]
**Leucyl aminopeptidase**	Salmonella enterica	Leu-Leu	918	[33]
**Leucyl aminopeptidase**	Salmonella enterica	Asp-Leu-Gly	950	[33]
**Leucyl aminopeptidase**	Bos taurus	Met-Gly	988	[32]
**Leucyl aminopeptidase**	Bos taurus	Met-Gly	988.3	[32]
**Leucyl aminopeptidase**	Solanum lycopersicum	Arg-Gly-Asp	3781	[34]
**Leucyl aminopeptidase**	Solanum lycopersicum	Gly-Leu	5140	[34]
**Leucyl aminopeptidase**	Solanum lycopersicum	Gly-Leu	5141	[34]
**Leucyl aminopeptidase**	Solanum lycopersicum	Arg-Gly-Phe	6070	[34]
**Leucyl aminopeptidase**	Solanum lycopersicum	Arg-Gly-Phe	6073	[34]
**Leucyl aminopeptidase**	Solanum lycopersicum	Gly-Gly	10860	[34]
**Leucyl aminopeptidase**	Solanum lycopersicum	Gly-Gly	10900	[34]
**Leucyl aminopeptidase**	Solanum lycopersicum	Arg-Gly	13500	[34]

**Leucyl aminopeptidase**	Solanum lycopersicum	Arg-Gly	13520	[34]
**Leucyl aminopeptidase**	Solanum lycopersicum	Leu-Gly-Gly	13960	[34]
**Leucyl aminopeptidase**	Solanum lycopersicum	Leu-Gly-Gly	14000	[34]
**Leucyl aminopeptidase**	Solanum lycopersicum	Leu-Gly	14100	[34]
**Leucyl aminopeptidase**	Solanum lycopersicum	Leu-Gly	14400	[34]
**Leucyl aminopeptidase**	Solanum lycopersicum	Leu-Gly	14440	[34]
**Leucyl aminopeptidase**	Solanum lycopersicum	Arg-Leu	19380	[34]
**Leucyl aminopeptidase**	Solanum lycopersicum	Arg-Leu	19400	[34]
**Leucyl aminopeptidase**	Solanum lycopersicum	Leu-Leu	34490	[34]
**Leucyl aminopeptidase**	Solanum lycopersicum	Leu-Leu	34500	[34]
**PepB aminopeptidase**	Salmonella enterica	Leu-Leu	918	[33]
**PepB aminopeptidase**	Salmonella enterica	Asp-Leu-Gly	950	[33]
**PepB aminopeptidase**	Salmonella enterica	Glu-Leu	866	[33]
**Tripeptide aminopeptidase**	Homo sapiens	Arg-Ser-Arg	0.56	[35]
**Tripeptide aminopeptidase**	Homo sapiens	Arg-Ala-Arg	5.3	[35]
**Tripeptide aminopeptidase**	Sus scrofa	Gly-Gly-Gly	2030	[36]

Methods S15: Parameters, related to Figure 1-4

The various parameters used in the phagocytosis model and the supporting simulations are described in the supplementary information and a subset is listed in Table S4.

Table S4: Parameter values.

Parameter	Value	Unit	References and Comments
N_{nutV}	2×10^9	N/A	[37], p. 223
V_{pred}	1000	μm^3	Typical volume of a eukaryote[38].
V_{prey}	1	μm^3	Typical volume of a prokaryote[38].
c_{prey}	0.0001	μm^{-3}	Prey concentration.
$N_{AA,prey}$	2×10^9	N/A	[37], p. 223
$N_{AA,enz}$	350	N/A	Typical protein length.
C_{AA}	29	ATP	Energetic cost of an average amino acid[25].
P_{nut}	0.88	N/A	Probability for nutrients of reaching the predator. This work.
P_{enz}	0.067	N/A	Probability for enzymes of reaching a single prey cell. This work.
A_{phago}	1.67	μm^2	Area occupancy of phagocytic cup at maximum extent for a prey volume of 1 μm^3. This work.
C_{axo}	1.83×10^{11}	ATP	Energy cost of an individual axopod. This work.
r_{axo}	0.14	μm	Radius of an axopod. This work, see Table S1 and Figure S1A.

A_{mp}	10^{-5}	μm^2	Area occupancy of individual Sec translocases and nutrient transporters.
k_{prot}	100	s^{-1}	Amino acid production rate by enzymes. See Table S3.
k_{trans}	10	s^{-1}	Nutrient transporter rate. Value unclear. Various sugar transporters have a range of 20-300 molecules s^{-1} (p. 224 in ref[37]). An in vitro estimate for aspartate transport[39] is 0.01 s^{-1}.
t_{ins}	20	s	Time of enzyme secretion by a Sec translocase[1,40].
$f_{memprot}$	0.4	N/A	Fraction of membrane area that is occupied by transmembrane proteins[41].
f_{budget}	1.5	N/A	Growth modifier to the cell budget. This work.
C_{area}	1.45x10^9	ATP μm^{-2}	Energy cost of membrane per unit area (includes lipids and proteins)[11].

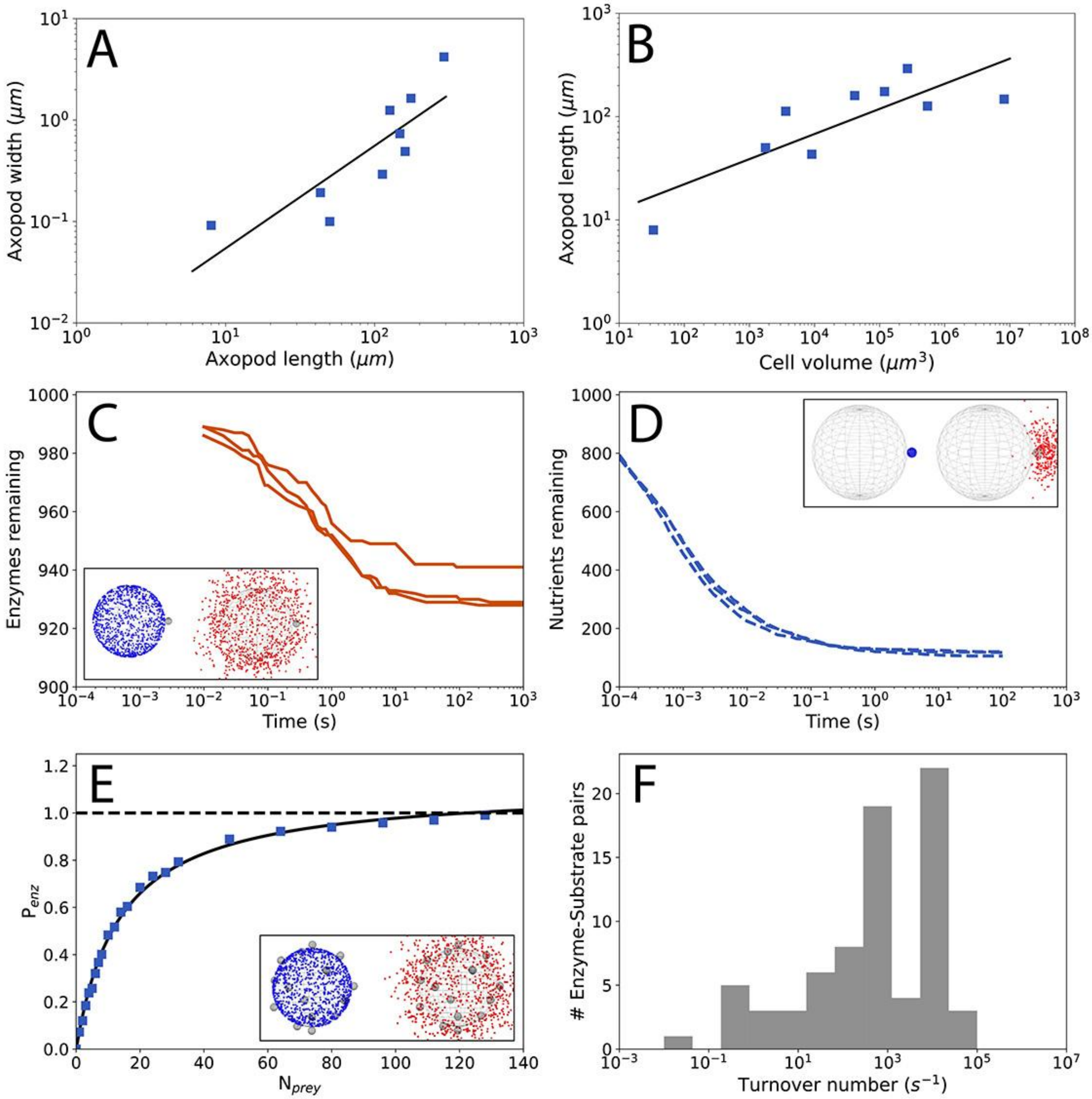

Figure S1: Axopod properties, enzyme and nutrient capture by cells, and enzyme turnover numbers. Related to Figure 1. A) Axopod lengths and widths for heliozoans[3–9]. Equation for the regression: $y = 0.0053\ x^{1.00}$. B) Heliozoan cell volumes and axopod lengths[3–9]. Equation for the regression: $y = 7.24\ x^{0.24}$. C) Smoldyn simulation results of enzyme finding prey, leading to a probability of finding prey, $P_{enz} = 0.067$. Inset: simulation setup with predator surface-attached enzymes in blue on the left, and free enzymes in red on the right. D) Smoldyn simulation results of nutrient finding the predator, leading to a probability of finding the predator, $P_{nut} = 0.88$. Inset: simulation setup with prey surface-attached nutrients in blue on the left, and free nutrients in red on the right. E) Smoldyn simulation results of enzyme finding prey, with varying prey number. Inset: simulation setup with predator surface-attached enzymes in blue on the left, and free enzymes in red on the right. F) Distribution of protease turnover numbers of enzyme-substrate pairs (Table S3).

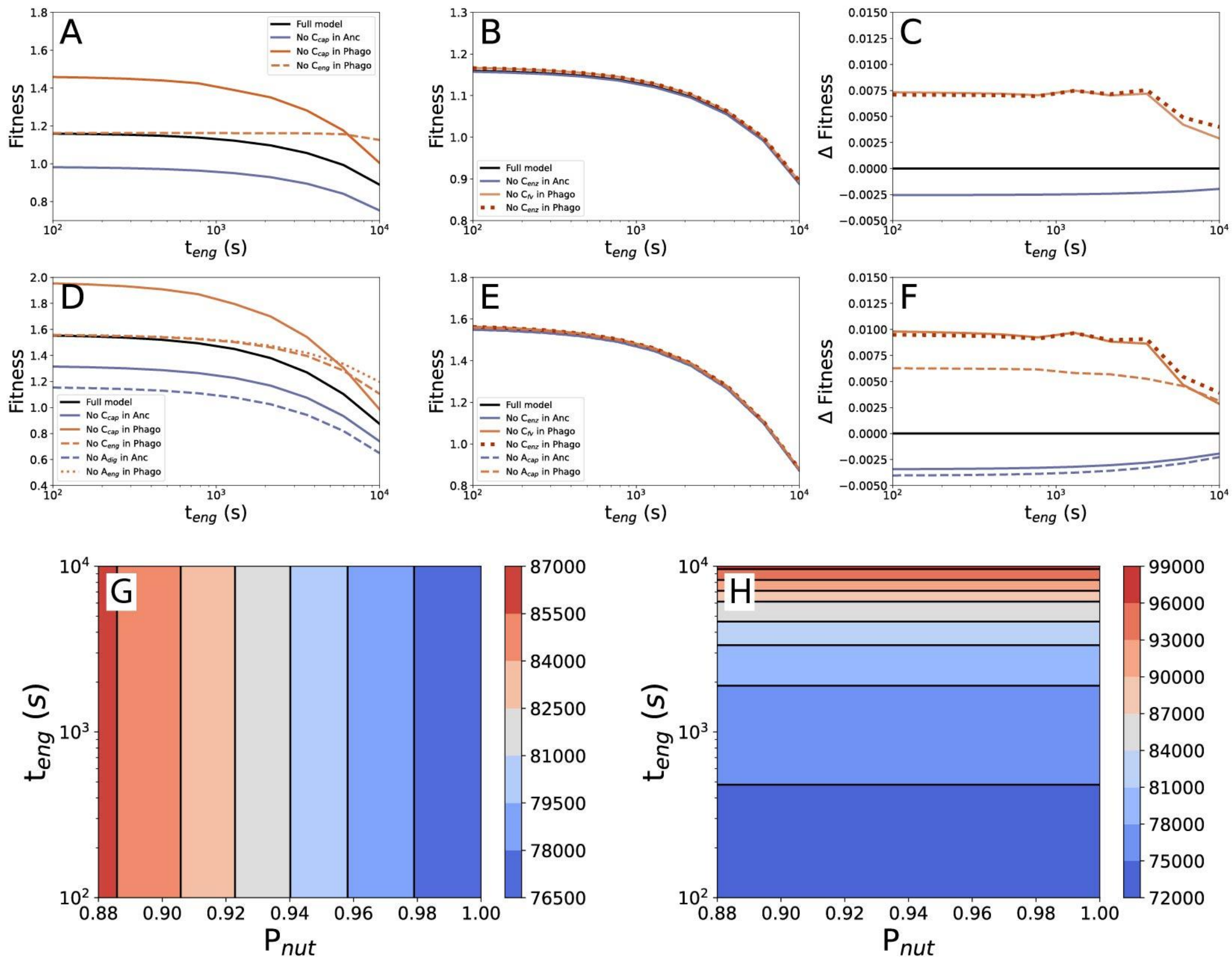

Figure S2: Analysis of cellular performance penalties associated with energy and surface area occupancy costs. Related to Figure 2. A-C) Cellular performance calculated using cell division times only. A) The full model compared to models lacking one of the larger cellular performance penalties. B) Comparison of full model to models lacking the smaller cellular performance penalties. C) Cellular performance difference by subtracting the full model cellular performance from the cellular performance of the models lacking the smaller cellular performance penalties (legend as in B). D-F) Cellular performance calculated using cell division times and surface area occupancies. D) The full model compared to models lacking one of the larger cellular performance penalties. E) Comparison of full model to models lacking one of the smaller cellular performance penalties. F) Cellular performance difference by subtracting the full model cellular performance from the cellular performance of the models lacking the smaller cellular performance penalties (legend as in E). G-H) Cell division times (in seconds) of the ancestor (G) and phagocytoser (H). Related to the cellular performance calculations in Figure 2F and 2G.

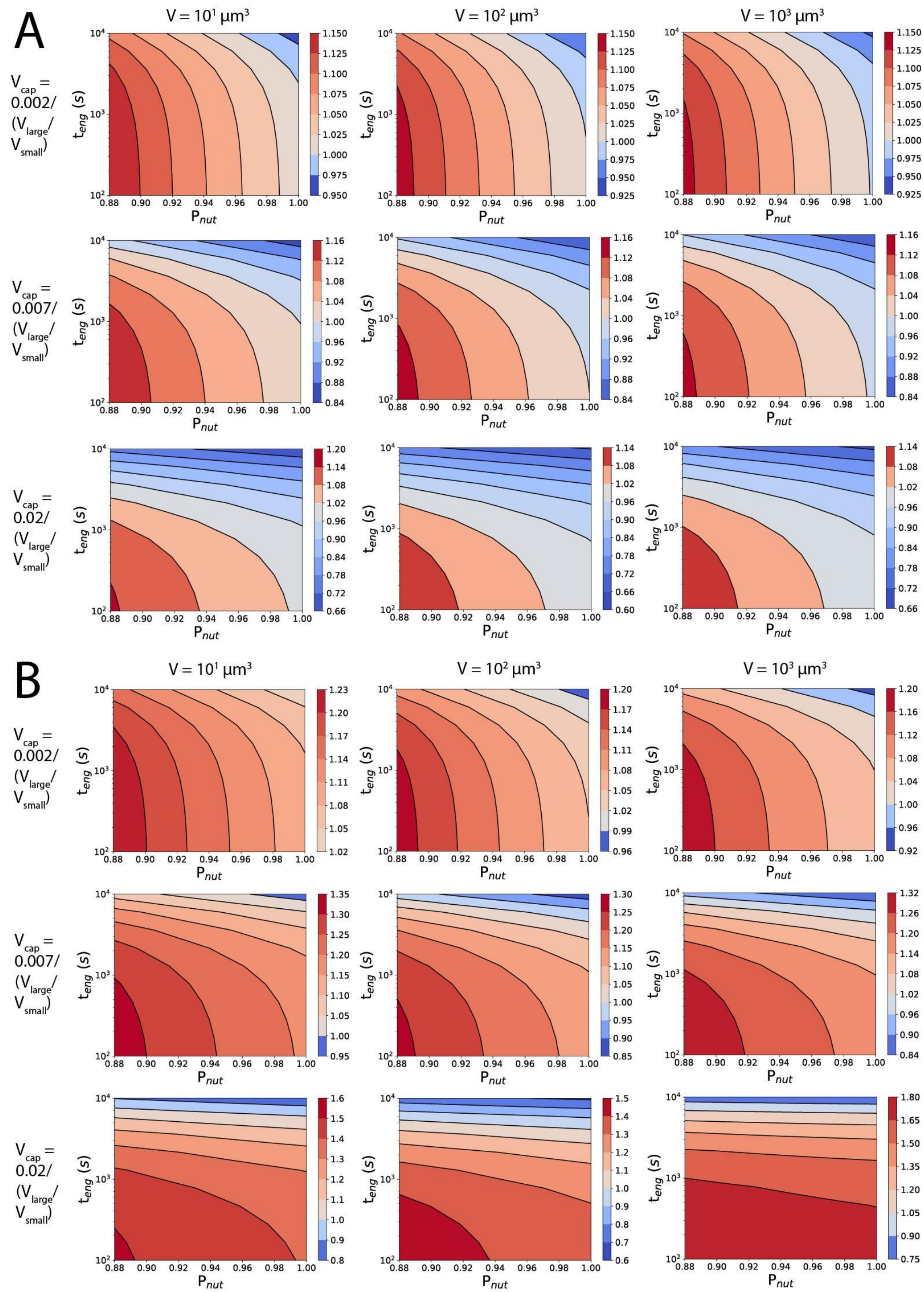

Figure S3: Cellular performance calculation for predators with a range of cell volumes. Related to Figure 4.

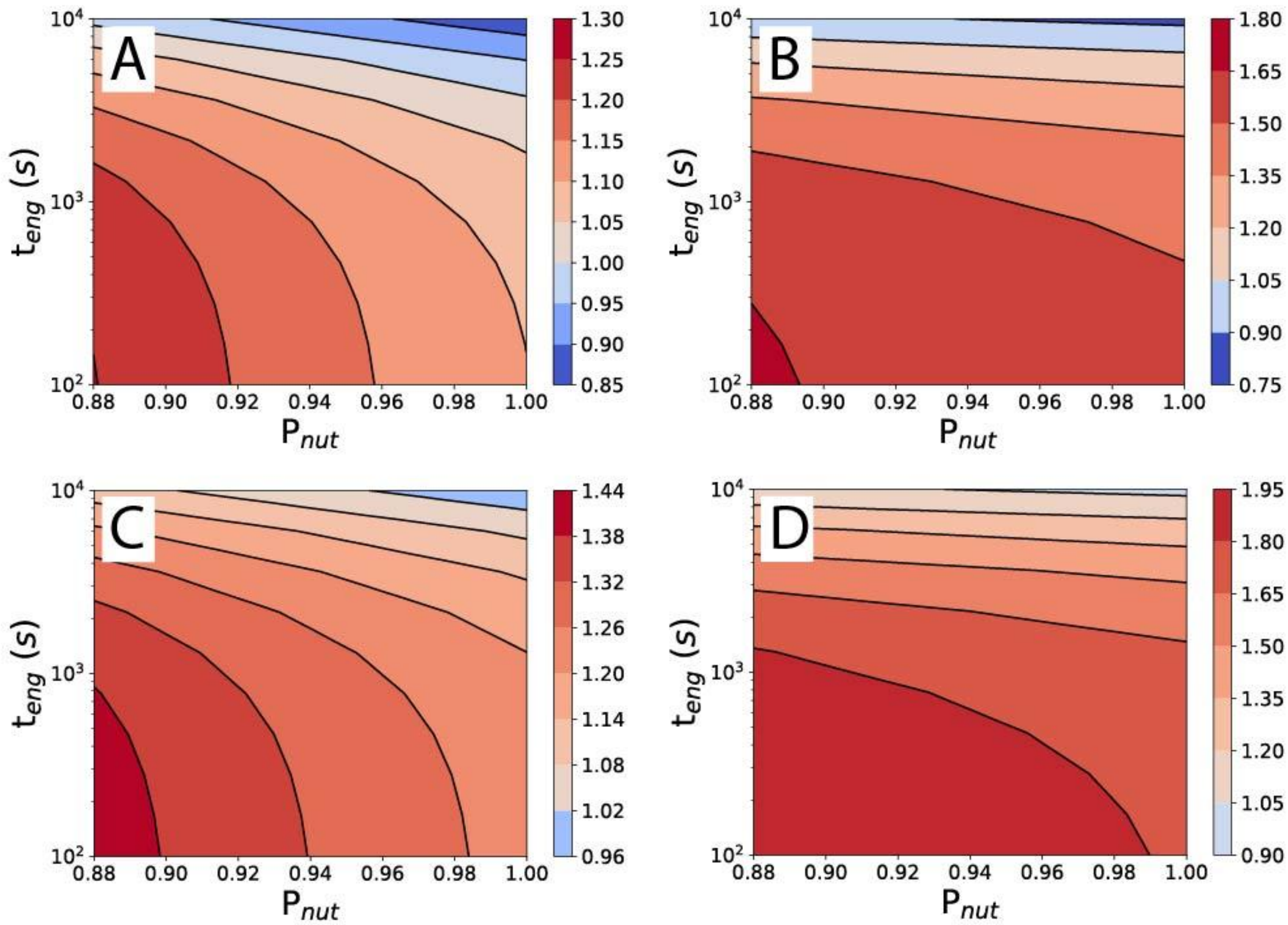

Figure S4: Cellular performance calculations for prey escape and digestion inefficiency. Related to Figure 4. A-B) Cellular performance when prey can escape the ancestor but not the phagocytoser. Cell division time only using Eq. 3 (A), and cell division time plus surface area constraints using Eq. 4 (B). C-D) Cellular performance when the ancestor is less efficient in extracting nutrients from prey than the phagocytoser. Cell division time only using Eq. 3 (C), and cell division time plus surface area constraints using Eq. 4 (D).

References

1. Schavemaker, P. E. & Lynch, M. Quantifying the evolutionary paths to endomembranes. *Cell Rep* **44**, (2025).

2. Andrews, S. S., Addy, N. J., Brent, R. & Arkin, A. P. Detailed simulations of cell biology with Smoldyn 2.1. *PLoS Comput Biol* **6**, (2010).

3. Yabuki, A., Chao, E. E., Ishida, K.-I. & Cavalier-Smith, T. Microheliella maris (Microhelida ord. n.), an ultrastructurally highly distinctive new axopodial protist species and genus, and the unity of phylum Heliozoa. *Protist* **163**, 356–388 (2012).

4. Davidson, L. A. Ultrastructure, behavior, and algal flagellate affinities of the helioflagellate Ciliophrys marina, and the classification of the helioflagellates (Protista, Actinopoda, Heliozoea) 1. *J Protozool* **29**, 19–29 (1982).

5. Khan, S. M. M. K. *et al.* Axopodial contraction in the heliozoon Raphidiophrys contractilis requires extracellular Ca2. *Zoolog Sci* **20**, 1367–1372 (2003).

6. Bardele, C. F. Cell cycle, morphogenesis, and ultrastructure in the pseudoheliozoan Clathrulina elegans. *Zeitschrift für Zellforschung und Mikroskopische Anatomie* **130**, 219–242 (1972).

7. Kinoshita, E., Shigenaka, Y. & Suzaki, T. The ultrastructure of contractile tubules in the heliozoon Actinophrys sol and their possible involvement in rapid axopodial contraction. *Journal of Eukaryotic Microbiology* **48**, 519–526 (2001).

8. Mikrjukov, K. A. & Patterson, D. J. Taxonomy and phylogeny of Heliozoa. III. Actinophryids. *Acta Protozool* **40**, 3–26 (2001).

9. Jones, W. C. The ultrastructure of Gymnosphaera albida Sassaki, a marine axopodiate protozoon. *Philosophical Transactions of the Royal Society of London. B, Biological Sciences* **275**, 349–384 (1976).

10. Berg, H. C. *Random Walks in Biology*. (Princeton University Press, 1993).

11. Schavemaker, P. E. & Lynch, M. Flagellar energy costs across the tree of life. *Elife* **11**, e77266 (2022).

12. Tilney, L. G. & Porter, K. R. Studies on the microtubules in Heliozoa II. The effect of low temperature on these structures in the formation and maintenance of the axopodia. *Journal of Cell Biology* **34**, 327–343 (1967).

13. Gast, R. J. Centrohelida and other heliozoan-like protists. *Handbook of the Protists* **2**, 955–971 (2017).

14. Schavemaker, P. E., Boersma, A. J. & Poolman, B. How important is protein diffusion in prokaryotes? *Front Mol Biosci* **5**, (2018).

15. Medalia, O. *et al.* Organization of actin networks in intact filopodia. *Current biology* **17**, 79–84 (2007).

16. Abraham, V. C., Krishnamurthi, V., Taylor, D. L. & Lanni, F. The actin-based nanomachine at the leading edge of migrating cells. *Biophys J* **77**, 1721–1732 (1999).

17. Mund, M. *et al.* Systematic nanoscale analysis of endocytosis links efficient vesicle formation to patterned actin nucleation. *Cell* **174**, 884–896 (2018).

18. Kasza, K. E. *et al.* Actin filament length tunes elasticity of flexibly cross-linked actin networks. *Biophys J* **99**, 1091–1100 (2010).

19. Hanson, J. & Lowy, J. The structure of F-actin and of actin filaments isolated from muscle. *J Mol Biol* **6**, 46–60 (1963).

20. Müller, J. B. *et al.* The proteome landscape of the kingdoms of life. *Nature* **582**, 592–596 (2020).

21. Eichinger, L. *et al.* The genome of the social amoeba Dictyostelium discoideum. *Nature* **435**, 43–57 (2005).

22. Sirotkin, V., Berro, J., Macmillan, K., Zhao, L. & Pollard, T. D. Quantitative analysis of the mechanism of endocytic actin patch assembly and disassembly in fission yeast. *Mol Biol Cell* **21**, 2894–2904 (2010).

23. Leadbeater, B. S. C. & Morton, C. A microscopical study of a marine species of Codosiga James-Clark (Choanoflagellata) with special reference to the ingestion of bacteria. *Biological Journal of the Linnean Society* **6**, 337–347 (1974).

24. Vorselen, D. *et al.* Phagocytic 'teeth'and myosin-II 'jaw'power target constriction during phagocytosis. *Elife* **10**, e68627 (2021).

25. Lynch, M. & Marinov, G. K. The bioenergetic costs of a gene. *Proceedings of the National Academy of Sciences* **112**, 15690–15695 (2015).

26. Hayman, S. & Patterson, E. K. Purification and properties of a mouse ascites tumor dipeptidase, a metalloenzyme. *Journal of Biological Chemistry* **246**, 660–669 (1971).

27. Das, M. & Radhakrishnan, A. N. Glycyl-L-leucine hydrolase, a versatile 'master'dipeptidase from monkey small intestine. *Biochemical Journal* **135**, 609–615 (1973).

28. Maurizi, M. R., Thompson, M. W., Singh, S. K. & Kim, S.-H. [23] Endopeptidase Clp: ATP-dependent Clp protease from Escherichia coli. in *Methods in enzymology* vol. 244 314–331 (Elsevier, 1994).

29. Jennings, L. D., Bohon, J., Chance, M. R. & Licht, S. The ClpP N-terminus coordinates substrate access with protease active site reactivity. *Biochemistry* **47**, 11031–11040 (2008).

30. Maurizi, M. R. Degradation in vitro of bacteriophage lambda N protein by Lon protease from Escherichia coli. *Journal of Biological Chemistry* **262**, 2696–2703 (1987).

31. Gur, E. & Sauer, R. T. Recognition of misfolded proteins by Lon, a AAA+ protease. *Genes Dev* **22**, 2267–2277 (2008).

32. Cappiello, M. *et al.* New role for leucyl aminopeptidase in glutathione turnover. *Biochemical Journal* **378**, 35–44 (2004).

33. Mathew, Z., Knox, T. M. & Miller, C. G. Salmonella enterica serovar Typhimurium peptidase B is a leucyl aminopeptidase with specificity for acidic amino acids. *J Bacteriol* **182**, 3383–3393 (2000).

34. Gu, Y.-Q. & Walling, L. L. Specificity of the wound-induced leucine aminopeptidase (LAP-A) of tomato: Activity on dipeptide and tripeptide substrates. *Eur J Biochem* **267**, 1178–1187 (2000).

35. Tholander, F. & Haeggström, J. Z. Assay for rapid analysis of the tri-peptidase activity of LTA4 hydrolase. *Proteins: Structure, Function, and Bioinformatics* **67**, 1113–1118 (2007).

36. Chenoweth, D., Mitchel, R. E. J. & Smith, E. L. Aminotripeptidase of Swine Kidney: I. Isolation and characterization of three different forms; utility of the enzyme in sequence work. *Journal of Biological Chemistry* **248**, 1672–1683 (1973).

37. Milo, R. & Phillips, R. *Cell Biology by the Numbers*. (Garland Science, 2015).

38. Schavemaker, P. E. & Muñoz-Gómez, S. A. The role of mitochondrial energetics in the origin and diversification of eukaryotes. *Nat Ecol Evol* **6**, 1307–1317 (2022).

39. Akyuz, N. *et al.* Transport domain unlocking sets the uptake rate of an aspartate transporter. *Nature* **518**, 68–73 (2015).

40. Schavemaker, P. E. & Poolman, B. (Membrane) Protein Production in Context. *Trends Biochem Sci* **43**, 858–868 (2018).

41. Lindén, M., Sens, P. & Phillips, R. Entropic Tension in Crowded Membranes. *PLoS Comput Biol* **8**, 1–10 (2012).

42. Lynch, M., Trickovic, B. & Kempes, C. P. Evolutionary scaling of maximum growth rate with organism size. *Sci Rep* **12**, 22586 (2022).
